# Comparative Transcriptional Profiling of *Bacillus cereus* Sensu Lato Strains during Growth in CO_2_-Bicarbonate and Aerobic Atmospheres

**DOI:** 10.1371/journal.pone.0004904

**Published:** 2009-03-19

**Authors:** Karla D. Passalacqua, Anjana Varadarajan, Benjamin Byrd, Nicholas H. Bergman

**Affiliations:** 1 School of Biology, Georgia Institute of Technology, Atlanta, Georgia, United States of America; 2 Electro-Optical Systems Laboratory, Georgia Tech Research Institute, Atlanta, Georgia, United States of America; BMSI-A*STAR, Singapore

## Abstract

**Background:**

*Bacillus* species are spore-forming bacteria that are ubiquitous in the environment and display a range of virulent and avirulent phenotypes. This range is particularly evident in the *Bacillus cereus* sensu lato group; where closely related strains cause anthrax, food-borne illnesses, and pneumonia, but can also be non-pathogenic. Although much of this phenotypic range can be attributed to the presence or absence of a few key virulence factors, there are other virulence-associated loci that are conserved throughout the *B. cereus* group, and we hypothesized that these genes may be regulated differently in pathogenic and non-pathogenic strains.

**Methodology/Principal Findings:**

Here we report transcriptional profiles of three closely related but phenotypically unique members of the *Bacillus cereus* group—a pneumonia-causing *B. cereus* strain (G9241), an attenuated strain of *B. anthracis* (Sterne 34F_2_), and an avirulent *B. cereus* strain (10987)—during exponential growth in two distinct atmospheric environments: 14% CO_2_/bicarbonate and ambient air. We show that the disease-causing *Bacillus* strains undergo more distinctive transcriptional changes between the two environments, and that the expression of plasmid-encoded virulence genes was increased exclusively in the CO_2_ environment. We observed a core of conserved metabolic genes that were differentially expressed in all three strains in both conditions. Additionally, the expression profiles of putative virulence genes in G9241 suggest that this strain, unlike *Bacillus anthracis*, may regulate gene expression with both PlcR and AtxA transcriptional regulators, each acting in a different environment.

**Conclusions/Significance:**

We have shown that homologous and even identical genes within the genomes of three closely related members of the *B. cereus* sensu lato group are in some instances regulated very differently, and that these differences can have important implications for virulence. This study provides insights into the evolution of the *B. cereus* group, and highlights the importance of looking beyond differences in gene content in comparative genomics studies.

## Introduction

*Bacillus* species are a highly diverse, spore-forming group of bacteria that can be found ubiquitously in the environment. These microbes range from being beneficial or benign to causing diseases such as anthrax, pneumonia, periodontal disease, and food-borne illness [1]–[3]. Much effort has been put into elucidating the pathogenic potential of one of the more notorious *Bacillus* species, *B. anthracis*, the causative agent of anthrax. However, in 1994, a *B. cereus* strain (G9241) was isolated from a welder suffering from severe pneumonia that was similar in many ways to inhalational anthrax, and other similar cases of anthrax-like disease caused by *B. cereus* have since been observed [4], [5]. Significantly, although the genomes of *B. cereus* G9241 and *B. anthracis* share many similarities [6], [7], the two species carry different plasmids, and they are phenotypically quite different (e.g., *B. cereus* G9241 is motile and hemolytic, and *B. anthracis* is neither). Given all of this, the relationship between *B. anthracis*, *B. cereus* G9241 and other related strains of *B. cereus* provides a unique perspective from which to study both the evolutionary origins and the genetic basis for phenotypic diversity in the *B. cereus* group.

Large-scale genomic variation amongst species is a primary source of bacterial identity, but phenotypic differences are also manifested by smaller differences that drive selective expression of gene repertoires. These subtle differences between strains of *B. cereus* and *B. anthracis* have not yet been explored. The global transcriptional behavior of *B. anthracis* has recently been investigated under a variety of conditions [8]–[12], and several studies have demonstrated that the presence of CO_2_/bicarbonate, either in the host or in vitro, induces the expression of genes encoding the anthrax toxin components [13], [14]. CO_2_/bicarbonate is the main pH buffering system in the body, and the relative levels of these molecules affect many cellular, biochemical and physiological processes [15]. Bicarbonate is a small and labile biomolecule that converts to CO_2_ readily in various conditions, both spontaneously and when catalyzed by carbonic anyhydrase enzymes, and pH levels are influenced as interconversion occurs [15]. It seems intuitive, therefore, that bacteria interacting with mammalian hosts (particularly pathogens) might have developed ways of responding to and thriving within a bicarbonate-rich environment that is most likely different from typical conditions encountered during growth in rich media in vitro. Indeed, both CO_2_ and bicarbonate have been shown to be important in the interaction between the host and a variety of bacteria, including *B. anthracis* [16], [17]. The full genome of the pathogenic *B. cereus* strain G9241 has been sequenced [6], but the transcriptional behavior of this microbe has not been elucidated.

Given the connection between CO_2_/bicarbonate and pathogenesis in *B. anthracis*, and the fact that the CO_2_/O_2_ balance is known to affect *B. anthracis* gene expression in a profound way, we felt that a global study of gene expression in varying atmospheric growth conditions would provide meaningful insights into the functional and transcriptional differences between strains of *B. cereus* and *B. anthracis*. With this in mind, in this study we characterized the relative transcriptional profiles of three closely related but phenotypically unique *Bacillus* strains between CO_2_/bicarbonate-rich and highly oxygenated atmospheres in a semi-defined growth medium. We assayed the growth characteristics of two pathogenic *Bacilli* (*B. cereus* G9241 and the attenuated *B. anthracis* strain Sterne 34F_2_) [6], [18], [19] as well as an avirulent laboratory strain, (*B. cereus* 10987) that is closely related to *B. anthracis* [5], [6], [20] in each condition, then generated relative mRNA profiles using Nimblegen gene expression microarrays. The data show that the two pathogens are, in terms of transcriptional regulation, more sensitive to atmospheric conditions than avirulent strain 10987, and that their plasmid gene expression is almost universally induced in the CO_2_/bicarbonate background. We identified 27 genes sharing >90% protein identity in all 3 species that were similarly regulated in the two growth conditions in all 3 strains, revealing a core repertoire of transcriptional changes for *Bacilli* between CO_2_ and O_2_. We also observed that groups of genes putatively involved in cellular characteristics such as motility, cell morphology, and pathogenesis were transcribed differentially between the strains. Of special note, we observed that many genes putatively regulated by the transcriptional regulators PlcR and AtxA were differentially expressed in strain G9241 in O_2_ and CO_2_, respectively. Therefore, unlike *B. anthracis*, strain G9241 may utilize both PlcR and AtxA transcriptional regulators, but in exclusive environments. This study refines our knowledge of *Bacillus* genome architecture and regulation and has highlighted specific functional aspects of two pathogenic species that should aid in focusing future research into the physiological aspects of *Bacillus* behavior.

## Results and Discussion

### Growth in Modified G Medium: CO_2_ versus O_2_

**Figure 1:**
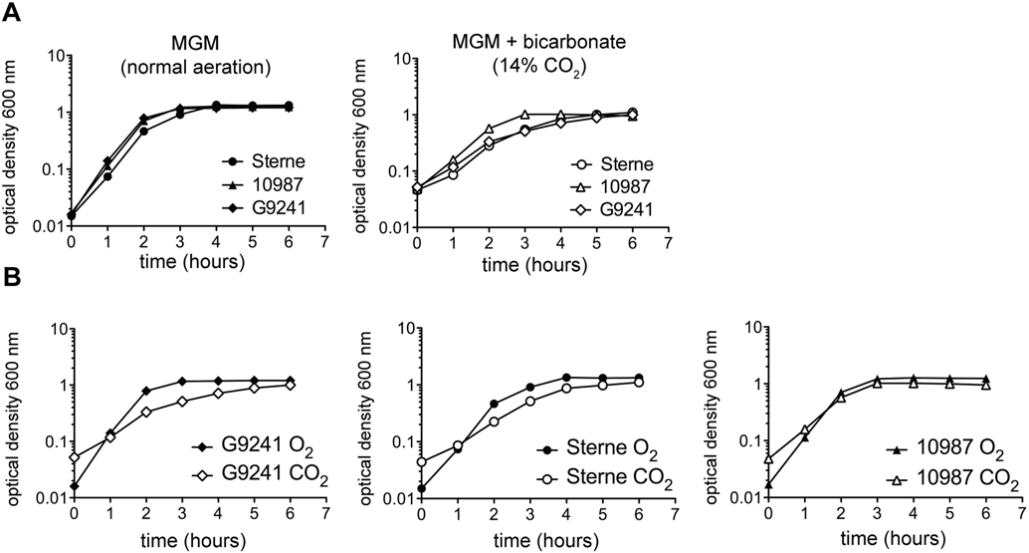
Growth curves of strains *B. cereus* G9241, *B. anthracis* Sterne 34F_2_, and *B. cereus* 10987 in MGM with normal aeration and with 14% CO_2_+0.8% bicarbonate. (A) The three *Bacillus* strains plotted against each other in each condition, exhibiting similar growth rates. (B) The three *Bacillus* species plotted against themselves in each condition, showing slightly slower growth rates for G9241 and *B. anthracis* Sterne in the CO_2_ environment. Curves are representative examples of 5 experiments with similar trends.

For characterization of the global transcriptional patterns of *B. cereus* G9241, *B. anthracis* Sterne 34F_2_, and *B. cereus* 10987 (hereafter referred to as G9241, *B. anthracis*, and 10987, respectively), we first determined the growth characteristics of these three strains in a semi-defined medium under both high aeration and 14% CO_2_+0.8% bicarbonate conditions (hereafter referred to as O_2_ and CO_2_). The medium used in this study, Modified G Medium (MGM), is typically used to produce high levels of *B. anthracis* spores (see Methods). This medium was used in earlier transcriptional profiling studies of *B. anthracis*, and its use here provides for continuity with those studies [8], [9]. Figure 1 illustrates the growth characteristics of the three *Bacilli* in MGM in O_2_ and CO_2_ (1 experiment representative of 5). Overall, the three strains grew at a slightly slower rate in CO_2_ than in O_2_, which is unsurprising, since energy production is generally more efficient in aerobic atmospheres. Depending on the time points used to calculate doubling times, rates of doubling in O_2_ ranged from 24 to 44 minutes, whereas doubling times in CO_2_ ranged from 31–55 minutes. However, within each growth condition, the strains displayed similar growth kinetics (Figure 1A), and they all reached stationary phase within 6 hours.

After observing that growth for each strain in each condition was robust, we sought to: (i) ascertain the major differences in the transcriptional profiles of each individual strain between growth in two very different atmospheres; and then (ii) make an inter-strain comparison of these overall transcriptional trends to ascertain differences and similarities between three closely-related but phenotypically unique *Bacilli*. Hence, because the strains grow at a slower rate in the CO_2_ atmosphere, we harvested RNA for transcriptome comparisons at equivalent population densities as measured by OD_600_ (∼0.4), which falls within the range of exponential growth for all strains. Microscopic examination of the strains in each condition did not indicate any overt morphological differences in either condition.

### Overview of gene expression in CO_2_ v O_2_ - chromosomes & plasmids

Microarrays are powerful tools for global gene expression studies, but they are limited by the fact that they require sequenced genomes and can only measure what is contained on the chip. Although the genome sequence for strain G9241 has been reported [6], it has not yet been entirely assembled or annotated, and as a consequence, the custom microarray made for us by Nimblegen has several features to be noted here. First, G9241 contains several extrachromosomal features; a cryptic phage (pBC_Clin29) and two large plasmids (pBCXO1 and pBC218). At the time of our microarray design, only a portion of the pBCXO1 plasmid sequence was available to us for probe selection; so of the 177 putative pBCXO1 genes, 111 (62%) are included on our array. Also note that plasmid pBC218 was originally reported in Hoffmaster et al. [6] as being ∼218 kb, but a subsequent study of *B. cereus* plasmids revealed that it is more likely ∼210 kb ([21], and Dave Rasko, personal communication). Hence, this genetic element has been reported as both pBC218 and pBC210 in the literature. Here, we refer to it by its original name, pBC218, since locus tag numbers in the public databases still refer to it as such.

**Figure 2:**
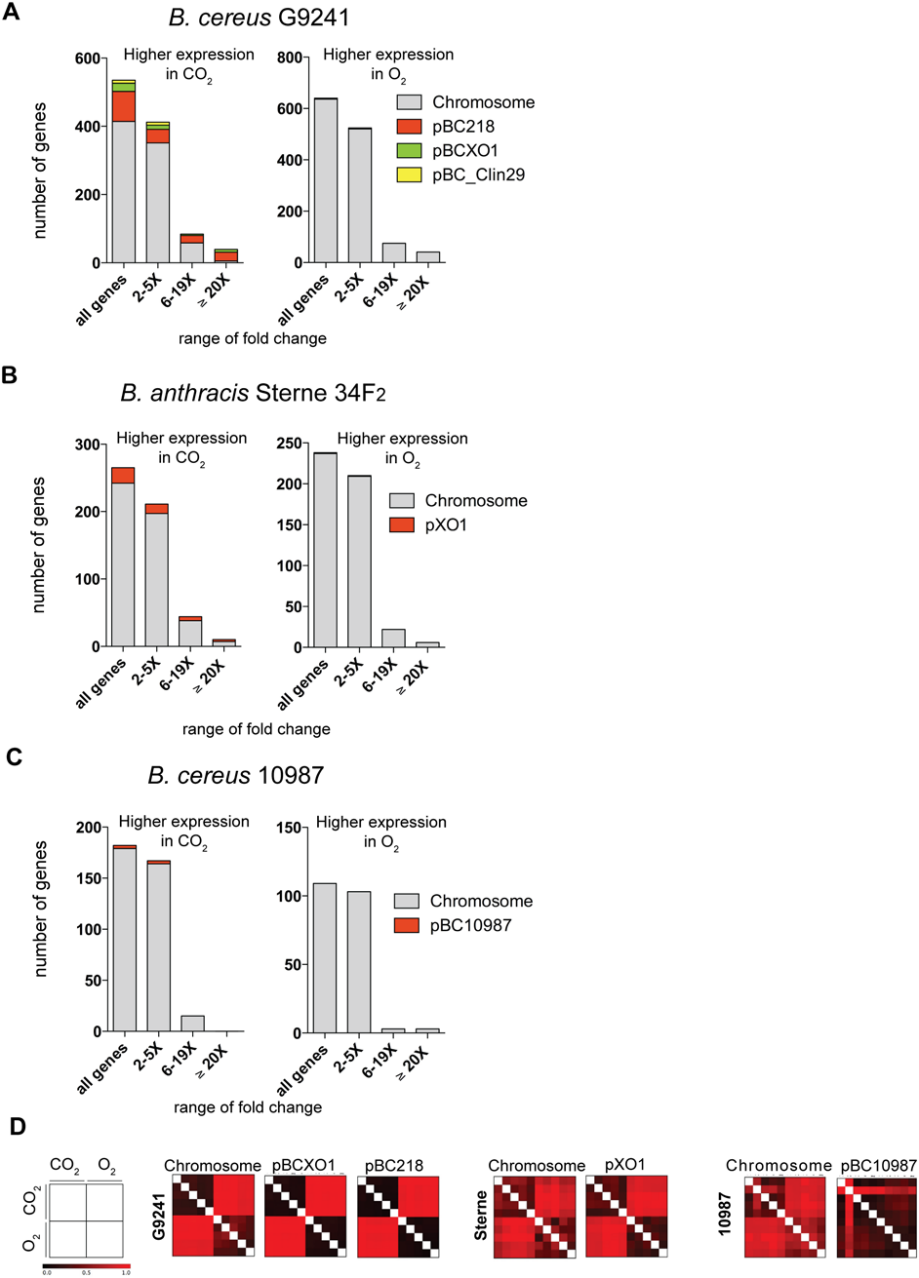
Global differences in transcription between CO_2_ and O_2_ growth conditions for three *Bacillus* strains. (A–C) Histograms show numbers of genes more highly expressed in CO_2_ and O_2_ conditions (per SAM analysis), displaying total number of genes, number of genes differentially expressed by ranges of Fold Difference, and numbers of genes from various genetic elements (chromosomes and plasmids). (D) Sample Density Matrices (SDM) for microarray samples comparing levels of similarity between CO_2_ and O_2_ conditions (black representing high similarity and red low similarity). Each quadrant is representative of 5 microarray experiments in each growth condition. See text for details on SDM.

Figure 2 and Table 1 outline the general gene expression patterns in terms of numbers of genes differentially expressed between growth in CO_2_ versus O_2_ for each of the three strains. In this primary analysis, we included all genes that were differentially expressed as assessed by SAM (Significance Analysis of Microarrays - see Methods) with differences in expression level >2-fold (5 microarrays each strain in each condition: 4 biological replicates plus 1 technical replicate - see Methods). Note that SAM analyses result in fold-difference comparisons that are reported relative to the sample that is used as the initial query. Hence, in our comparisons, genes with decreased expression in CO_2_ were viewed as having increased expression in O_2_, and are referred to as such for clarity.

**Table 1:** Numbers of genes differentially expressed for 3 *Bacillus* strains during log-phase growth in MGM in CO_2_+bicarbonate versus O_2_.

*B. cereus* G9241								
	Total	Total	2–5 fold	2–5 fold	6–19 fold	6–19 fold	>20 fold	>20 fold
	higher in CO_2_	higher in O_2_	higher in CO_2_	higher in O_2_	higher in CO_2_	higher in O_2_	higher in CO_2_	higher in O_2_
**All genes**	535 (9%)*	640 (11%)*	412	524	84	75	39	41
**Chromosome**	414	636	351	520	58	75	5	41
**pBC218**	88	0	40	0	22	0	26	0
**pBCXO1**	24	2	12	2	4	0	8	0
**pBC Clin29**	9	2	9	2	0	0	0	0

*B. anthracis* Sterne 34F_2_								
	Total	Total	2–5 fold	2–5 fold	6–19 fold	6–19 fold	>20 fold	>20 fold
	higher in CO_2_	higher in O_2_	higher in CO_2_	higher in O_2_	higher in CO_2_	higher in O_2_	higher in CO_2_	higher in O_2_
**All genes**	265 (5%)*	238 (4%)*	211	210	44	22	10	6
**Chromosome**	242	237	197	209	38	22	7	6
**pXO1**	23	1	14	1	6	0	3	0

*B. cereus* 10987								
	Total	Total	2–5 fold	2–5 fold	6–19 fold	6–19 fold	>20 fold	>20 fold
	higher in CO_2_	higher in O_2_	higher in CO_2_	higher in O_2_	higher in CO_2_	higher in O_2_	higher in CO_2_	higher in O_2_
**All genes**	182 (3%)*	109 (2%)*	167	103	15	3	0	3
**Chromosome**	179	109	164	103	15	3	0	3
**pBC 10987**	3	0	3	0	0	0	0	0

* Percentages in parentheses for “All genes” represent the percentage of genes relative to the entire expression array.

Strain G9241 showed the greatest overall difference in expression profile between the two growth atmospheres (535 genes up in CO_2_ v 640 up in O_2_), followed by *B. anthracis* (265 up in CO_2_ v 238 up in O_2_), with 10987 showing the smallest difference in gene expression between the two conditions (182 up in CO_2_ v 109 up in O_2_) (Table 1). Given that G9241 is a recent clinical isolate, and has not been passaged for years under laboratory conditions as have the commonly used *B. anthracis* Sterne and *B. cereus* 10987 strains, the more robust differences in transcriptome profiles are perhaps not surprising. Looking more closely at these data, we noted that G9241 also induced genes at significantly higher levels (6–19 fold and >20 fold) in each condition than either *B. anthracis* or 10987 (Fig. 2A–C, Table 1). More interestingly, we observed that plasmid genes for G9241 and *B. anthracis* are almost exclusively expressed more highly in CO_2_ (Fig. 2A and B, Table 1). For G9241, 39 genes were expressed >20 fold higher in CO_2_, and 34 of them (∼87%) were plasmid-encoded. No genes from pBC218 were expressed higher in O_2_ at any level. For *B. anthracis*, 23 genes on pXO1 were more highly expressed in CO_2_, but only 9 of these were at levels >6 fold. (Fig. 2B, Table 1).

Global differences between transcriptional profiles in CO_2_ and O_2_ environments can be more clearly seen in sample distance matrices (SDM), which allowed us to directly visualize the overall similarity/dissimilarity between global expression profiles. Figure 2D shows SDMs for each chromosome and plasmid carried by the three strains, with the 5 separate microarray datasets arranged from top to bottom and left to right in the same order. The diagonal represents self-self comparisons and is left blank, while all other squares are colored based on the similarity between the two datasets that are represented by the row and column that intersect there (with black indicating high similarity and red indicating low similarity). Thus, the upper left and lower right quadrants show comparisons of the 5 replicate datasets collected in the same atmospheric condition, (i.e., CO_2_ v CO_2_ and O_2_ v O_2_); whereas the upper right and lower left quadrants show comparisons between the gene expression profiles observed under different atmospheric conditions (i.e., CO_2_ v O_2_). The SDM show that all three G9241 genetic elements had a striking change in global expression profile between CO_2_ and O_2_, as was suggested in the numbers of genes differentially expressed (Fig. 2, Table 1). In other words, in terms of overall expression, G9241 shows very different expression patterns in each of the two conditions. *B. anthracis* also showed a significantly different expression profile between the two conditions for both chromosomal and plasmid-encoded genes, but to a slightly lesser degree than G9241 (Fig. 2D). Strain 10987 showed more subtle differences in chromosomal gene expression between CO_2_ and O_2_, but expression from the pBC10987 plasmid appears to be quite similar in all samples. This plasmid is derived from the pXO1/pBCXO1 group [20], [21], but at least in terms of gene expression in CO_2_, it appears to have diverged considerably from its pathogenic relatives.

Whereas 88 genes on G9241's pBC218 plasmid showed increased expression in CO_2_ (out of 188 putative ORFs, ∼47%), and pBCXO1 had 24 genes upregulated (24 out of 111, ∼22%), we see that only a select region of the *B. anthracis* pXO1 plasmid showed an increase in expression (23 out of 204 ORFs, ∼11%) in CO_2_. Table 2 lists all plasmid genes for pBC218 (G92410), and Table 3 lists all genes for pBCXO1 (G9241) and pXO1 (*B. anthracis*) plasmids that had increased expression in CO_2_ >6-fold, and we noted several salient observations. First, genetic loci 0059–0073 from G9241's pBC218 represent a putative gene cluster for capsule biosynthesis [6], [22], and each of these showed substantially increased expression in CO_2_ (9–77 fold) (Table 2 footnote *b*). Sue et al. [22] observed (but did not quantify) capsule production in G9241 in both the presence and absence of CO_2_, in a variety of growth media, and our transcriptional data extend that finding by suggesting that CO_2_ may cause an increase in capsule production. More interestingly, we noted an extraordinary increase in expression in G9241 of three genes on the pBCXO1 plasmid (loci pBCXO1_0108-0110; up 123–226 fold) that belong to a family of transferases with undefined function (Table 3). Considering the dynamic range of measurements made by microarrays, fold-differences this high are rarely observed and indicate extreme differences in relative transcript abundance. Interestingly, one of these genes - *galU* (pBCXO1_0109) - shares 100% protein identity with the *B. anthracis* pXO1 *galU* gene (pXO1_0129), which was the only *B. anthracis* plasmid gene that showed higher expression in O_2_ (2.8 fold). Although the G9241 pBCXO1 and *B. anthracis* pXO1 plasmids differ slightly in terms of sequence [6], they appear to have diverged much more in terms of transcriptional regulation, at least as can be seen between O_2_ and CO_2_ growth (Table 3). This may be linked to the acquisition by *B. anthracis* of the pXO2 virulence plasmid, which contains genes that encode the enzymes necessary for the synthesis of a very different capsule [23] and its regulators [24]. It is tempting to speculate that these highly expressed pBCXO1 genes in G9241 might possibly be involved in augmenting pBC218 encoded capsule production for this strain, [5], and it may be interesting to explore the possibility of a synergistic relationship between pBCXO1 and pBC218 in capsule formation. Some genes located on G9241's pBC218 plasmid also displayed what can be considered extreme differences in transcript abundance in CO_2_ (>50 fold) (Table 2), including several hypothetical proteins, a non-hemolytic enterotoxin component (see section “Pathogenesis genes”), and an *arsR* transcriptional regulator (locus 0045). Overall, these data show that the pathogenic *Bacillus* strains have evolved ways to adapt gene expression in a more profound and extensive way in different atmospheric conditions. Avirulent strain 10987, on the other hand, stands in stark counterpoint to the pathogens, where relative transcriptional activity between CO_2_ and O_2_ appears to be only slightly regulated.

**Table 2:** Genes with increased expression (≥6-fold) for the *B. cereus* G9241 pBC218 plasmid in CO_2_+0.8% bicarbonate.

*B. cereus* G9241 - pBC218		
apBC218 locus tag	gene name	fold difference
0008	conserved hypothetical protein	8.62
0010	hypothetical protein	47.05
0011	hypothetical protein	50.77
0012	*pXO2-42*; S-layer_protein / peptidoglycan endo-beta-N- acetylglucosaminidase	28.56
0013	periplasmic component of efflux system	11.27
0024	protease HhoA	7.46
0025	hypothetical protein	53.30
0026	protective antigen	40.13
0027	lethal factor precursor	30.70
0034	pVS1 resolvase	6.11
0039	enterotoxin B	42.88
0040	non-hemolytic enterotoxin lytic component L1	75.72
0042	non-hemolytic enterotoxin lytic component L2 putative	24.70
0043	S-layer homology domain protein	41.00
0045	*pXO1-109*; transcriptional regulator arsR family	54.40
0046	membrane protein putative	77.91
0047	probable electron transfer protein Rv1937 putative	89.28
0048	*cysK*; cysteine synthase A	50.40
0055	*yngK-1*; YngK protein	6.87
0057	pXO1 ORF14-like protein	25.96
0059^b^	polysaccharide translocase protein putative	39.13
0060^b^	CMP-sialic acid synthetase putative	40.68
0061^b^	UDP-N-acetylglucosamine 2-epimerase putative	44.33
0062^b^	sialic acid synthase putative	18.34
0063^b^	glycosyl transferase putative	77.14
0064^b^	*galU*; UTP-glucose-1-phosphate uridylyltransferase	42.26
0065^b^	exopolysaccharide biosynthesis protein	57.93
0066^b^	*wzz*; chain length determinant protein	39.44
0067^b^	transcriptional regulator LytR family	21.08
0068^b^	hypothetical protein	8.71
0069^b^	polysaccharide capsule synthesis protein	22.30
0070^b^	hypothetical protein	13.21
0071^b^	glycosyl transferase WecB/TagA/CpsF family	11.79
0072^b^	*wzy*; polysaccharide polymerase	9.07
0073^b^	glycosyl transferase group 2 family protein	15.82
0075	first of two overlapping orfs with similarity to IS3 genes	10.51
0076	transposase	10.37
0079	transposase (IS4 family)	7.00
0081	*dnaX*; DNA polymerase III gamma and tau subunits	7.30
0082	*tnpB*; transposase all7245	11.06
0104	geranylgeranyl hydrogenase BchP putative	7.68
0107	hypothetical protein	6.12
0119	lipoprotein NLP/P60 family	28.54
0120	membrane protein putative	22.39
0121	hypothetical protein	6.17
0135	*pXO1-42*; conjugation protein TraG/TraD family	11.36
0136	hypothetical protein	7.68
0174	conserved hypothetical protein	16.54

a Locus tag numbers are as follows for the pBC218 plasmid of *B. cereus* G9241: BCE_G9241_pBC218_XXXX.

b pBC218 loci 0059-0073 are genes putatively responsible for capsule production in G9241 [6].

**Table 3:** Genes with increased expression in CO_2_+0.8% bicarbonate (≥6-fold) for the *B. cereus* G9241 pBCXO1 plasmid and the *B. anthracis* pXO1 plasmid.

*B. cereus* G9241 - pBCXO1 plasmid		
apBCXO1 locus tag	gene name	fold difference
0105	S-layer homology domain protein (99% protein i.d. w/ *B. anthracis* pXO1 0124)	12.59
0106	PAP2 superfamily domain protein (100% protein i.d. w/ *B. anthracis* pXO1 0125)	9.54
0108	glycosyl transferase group 2 family protein domain	172.58
0109^b^	*galU*; UTP-glucose-1-phosphate uridylyltransferase	226.82
0110	UDP-glucose/GDP-mannose dehydrogenase family NAD binding domain family	123.46
0115	conserved hypothetical protein	8.68
0116	conserved hypothetical protein	25.80
0117	conserved hypothetical protein	53.08
0118	conserved hypothetical protein	40.10
0119	*cya*; calmodulin-sensitive adenylate cyclase	10.58
0120	*apt*; adenine phosphoribosyltransferase	27.10
0121	*apt*; adenine phosphoribosyltransferase	26.53
0126^d^	conserved hypothetical protein (100% identical at nucleotide level to *B. anthracis* pXO1 0146 *AtxA*)	5.6

*B. anthracis* - pXO1 plasmid		
apXO1 locus tag	gene name	fold difference
0123	hypothetical protein	40.58
0124^c^	S-layer protein, (pxo1-90)	35.13
0125	phosphatase, PAP2 family, (pxo1-91)	12.03
0137	hypothetical protein	48.25
0146^d^	transcriptional activator *atxa*, (pxo1-119)	5.24
0153	hypothetical protein	9.53
0164	protective antigen	11.80
0165	hypothetical protein	11.85
0166	transcriptional repressor *pagr*, (pxo1-109)	15.07
0171	ribonuclease domain protein	6.05
0172^d^	lethal factor	5.34

a Locus tag numbers are as follows for the plasmids listed above. For *B. cereus* G9241, (i) BCE_G9241_pBCXO1_XXXX; and (ii) for *B. anthracis*, the pXO1 sequence is from the Ames Ancestor genome, and the full locus tags read as GBAA_pXO1_XXXX.

b Note that this gene shares 100% protein identity with the *B. anthracis* gene pXO1_0129, which is the only pXO1 gene that was more highly expressed in O_2_ (2.8 fold).

c Referred to as *bslA* in [36].

d Although pBCXO1_0126, and the *B. anthracis* loci for *atxA* and lethal factor are slightly below a 6-fold difference, we include them here due to their importance in *B. anthracis* gene expression and virulence. Also note that the third toxin component, *cyaA* (pXO1_0142) was expressed more highly in CO_2_ by only 2-fold compared to the other toxin components, protective antigen and lethal factor.

### Core *Bacillus* gene expression

**Figure 3:**
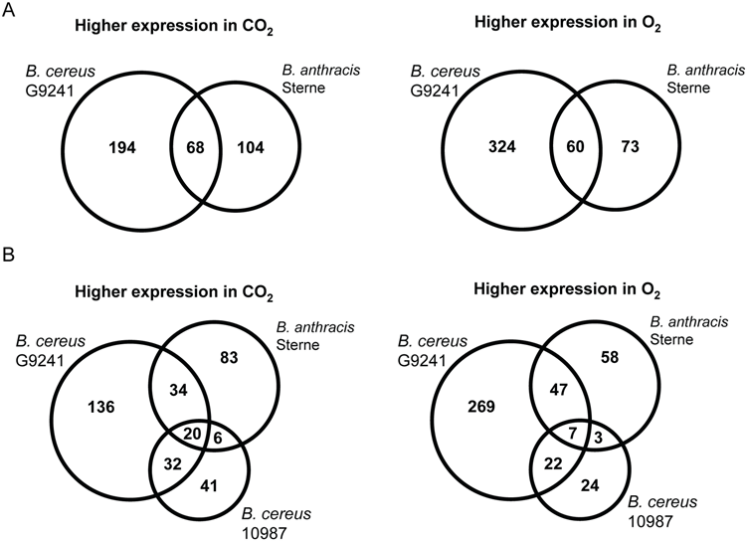
Venn diagrams representing numbers of genes differentially expressed in CO_2_ and O_2_ that share >90% protein identity in the *Bacillus* strains indicated. (A) Comparisons of the two pathogenic strains, *B. cereus* G9241 and *B. anthracis* (Sterne 34F_2_) from a list of 3,076 putatively common genes. (B) Comparisons of all three *Bacillus* strains (including *B. cereus* 10987) from a list of 2,624 putatively common genes.

Comparing transcriptomes of phenotypically unique but closely related bacteria [25] is not a common approach for microarray studies, but here it provides a perspective on core expression programs that are used by related strains in adapting to environmental stimuli. Therefore, with expression data for three unique microbes in hand, we asked what core similarities exist amongst these three related but unique strains that could indicate a highly conserved set of genes important for adaptation to varying atmospheric conditions for the *Bacillus cereus* group. First, we performed genome-wide BLASTp analysis and generated 2 lists: (i) all genes that share >90% protein identity between the two pathogens G9241 and *B. anthracis* (n = 3,076 genes); and (ii) a list of genes that share >90% protein identity in all 3 strains (n = 2,624) (see Methods). We then compared these lists to each other and identified the genes that were both conserved and differentially expressed in each strain in CO_2_ and O_2_. Finally, we compared the conserved and expressed gene lists to each other to identify common genes that were differentially expressed in CO_2_ and O_2_ in all 3 strains. Figure 3 shows four Venn diagrams outlining these results, and Tables 4, S1 and S2 list specific genes with fold-differences.

**Table 4:** List of conserved genes (>90% protein identity) that are differentially expressed in CO_2_ and O_2_ environments in all 3 *Bacillus* strains. (>2-fold).

Gene expression in CO_2_ relative to O_2_						
Gene name	*B. cereus* G9241		*B. anthracis*		*B. cereus* 10987	
	alocus tag	bFD	alocus tag	bFD	alocus tag	bFD
cation-transporting Atpase, e1-e2 family	0415	**8.04**	0405	**8.06**	0519	**8.46**
hypothetical protein	0776	**5.99**	0789	**3.37**	0880	**4.68**
hypothetical protein	1031	**39.89**	1006	**4.13**	1107	**3.13**
cacyl-CoA synthase	1092	**2.48**	1091	**7.24**	1193	**5.42**
hypothetical protein	1351	**15.25**	1354	**6.18**	1453	**4.27**
dmenaquinol-cytochrome c reductase, cytochrome b/c subunit	1544	**4.62**	1546	**2.50**	1651	**2.40**
egermination protein gerN	1647	**5.26**	1639	**11.70**	1729	**14.02**
f2-hydroxy-3-oxopropionate reductase	2318	**2.45**	2353	**2.15**	2381	**3.19**
methylmalonic acid semialdehyde dehydrogenase	2319	**3.31**	2354	**2.21**	2382	**2.50**
hypothetical protein	2788	**3.67**	2839	**9.82**	2869	**7.76**
hypothetical protein	2789	**2.82**	2840	**10.30**	2870	**6.83**
anaerobic ribonucleoside triphosphate reductase	3555	**8.06**	3663	**11.83**	3622	**8.40**
gsulfatase	3736	**8.40**	3895	**7.85**	3797	**4.01**
cytochrome c oxidase, subunit iii	3931	**3.72**	4152	**2.41**	3989	**4.07**
cytochrome c oxidase, subunit i	3932	**3.77**	4153	**2.27**	3990	**3.34**
cytochrome c oxidase, subunit ii	3933	**3.06**	4154	**2.03**	3991	**2.86**
acetyl-Coa synthetase, putative	4729	**4.67**	4896	**2.98**	4781	**3.41**
hypothetical protein	4923	**4.13**	5071	**2.90**	4973	**2.32**
acyl-Coa dehydrogenase	5102	**7.44**	5246	**2.44**	5142	**12.47**
3-hydroxyacyl-Coa dehydrogenase/enoyl-coa hydratase/isomerase family protein	5105	**12.11**	5249	**2.09**	5144	**11.77**

Gene expression in O_2_ relative to CO_2_						
Gene name	*B. cereus* G9241		*B. anthracis*		*B. cereus* 10987	
	alocus tag	bFD	alocus tag	bFD	alocus tag	bFD
transcriptional regulator, MarR family	0645	**8.47**	0662	**4.02**	0730	**3.23**
transcriptional regulator, GntR family	1313	**2.97**	1314	**2.49**	1415	**2.09**
hypothetical protein	2128	**6.73**	2118	**4.39**	2201	**3.09**
oxalate∶formate antiporter, putative	2332	**19.88**	2367	**77.66**	2396	**78.18**
hgamma-glutamyl kinase	2947	**2.47**	2993	**3.29**	3029	**3.16**
5′-nucleotidase, putative	3096	**2.41**	3162	**3.56**	3157	**2.37**
endonuclease/exonuclease/phosphatase family	5186	**6.57**	5318	**3.46**	5215	**2.70**

a Locus tag numbers only from *B. cereus* G9241 (BCE_G9241_XXXX), *B. anthracis* (GBAAXXXX) Ames Ancestor, and *B. cereus* 10987 (BCE_XXXX).

b Fold Difference as assessed by SAM (see Methods).

c Named “long-chain-fatty-acid–CoA ligase” in G9241 annotation.

d Named “cytochrome b–c complex, cytochrome b subunit, putative” in G9241 annotation.

e Named “Na+/H+ antiporter” in G9241 annotation.

f Named “3-hydroxyisobutyrate dehydrogenase” in G9241 annotation.

g Named “phosphoglycerol transferase” in G9241 annotation.

h Named “glutamate 5-kinase” in G9241 annotation.

In comparing the two pathogenic strains, G9241 and *B. anthracis* (Fig. 3A), 68 common genes were more induced in CO_2_, and 60 common genes were more induced in O_2_ (Tables S1 and S2). Of note are several genes that were substantially (>20 fold) induced in the two conditions. Two ATP-binding transporters (*cydC* and *cydD*) were substantially up-regulated in CO_2_ in these strains (29–65 fold), suggesting that as in *B. subtilis*, the cytochrome bd complex may be active under CO_2_ conditions [26]. Also, our data suggest that L-lysine catabolism is important for growth in CO_2_ in MGM for G9241 and *B. anthracis*, since the *kamA* transcript (L-lysine 2,3-aminomutase) is >23-fold more abundant in this atmosphere [27] (Table S1). In the O_2_ environment, the most conspicuous genes conserved and differentially expressed in the two pathogens were two genes putatively involved in trehalose transport and metabolism (*treB* and *treC*) (Table S2), revealing another way in which the two pathogens have adopted modes of gene regulation not observed in strain 10987.

For genes that are conserved in all three strains, 20 were commonly induced in CO_2_, while 7 were common in O_2_ (Fig. 3B, Table 4). These data suggest that the various cytochrome c oxidases, reductases, CoA synthetase and dehydrogenase genes are metabolic proteins most important to *Bacilli* in a CO_2_/bicarbonate atmosphere. Two specific genes jump out as perhaps being of utmost importance for growth in each conditions: (i) an anaerobic ribonucleoside triphosphate reductase in CO_2_, and (ii) an oxalate∶formate antiporter in O_2_.

The biochemical properties of anaerobic ribonucleoside triphosphate reductase have been studied extensively in *E. coli* [28]–[31], and this enzyme is needed for the making of deoxyribonucleoside triphosphates in anaerobic conditions for this microbe. Considering the substantial upregulation of this gene in CO_2_ (8–11 fold difference) in all three strains, it is likely of critical importance for *Bacillus* replication in a CO_2_/bicarbonate environment. Previously, the ribonucleotide reductase gene *NrdF* of *B. anthracis* was characterized and suggested as a possible antimicrobial target [32]. However, because the CO_2_/bicarbonate environment is considered to be somewhat analogous to conditions within the host [16], this other nucleic acid gene/enzyme may be another candidate as a possible target for antimicrobial reagents for all pathogenic *Bacilli*. Regarding the putative oxalate∶formate antiporter more highly expressed in O_2_ in all three strains, special note should be taken of the label “putative”, since the COG term for this gene is listed as “nitrate/nitrite antiporter”, and the InterPro and Pfam descriptions are simply listed as “Major Facilitator Superfamily”. Hence, it is difficult to say whether or not this transporter specifically moves oxalate/formate, but the conserved expression in an O_2_ environment suggests that it is an important transporter in an aerobic environment. However, because this type of growth is not considered to be analogous to the host, the conserved expression of this gene is mainly illustrative of the general metabolic capabilities and gene regulation in the *Bacilli*.

### Relative transcriptional profiles in CO_2_ versus O_2_ (≥6-Fold Difference)

The previous global analyses outlined differential gene expression profiles for the three *Bacillus* strains in a broad and global view, and included all genes with fold differences >2 between CO_2_ and O_2_. We list in Tables 5, 6, 7, 8, 9 those genes with >6-fold difference between the two growth states (CO_2_ and O_2_) by putative biological & cellular function for each strain. They are organized first by broad putative functional family (gene families include Energy & Metabolism, Spore Function, Cellular & Enzymatic Activity, Amino Acids, Nucleic Acids, Transport, Regulation, Motility & Chemotaxis, and Pathogenesis & Toxins), then by *Bacillus* strain, and then by more specific COG, GO or Pfam categories (Genes of “Unknown Function” and “Hypothetical Proteins” expressed >6-fold are listed in Tables S3 and S4). The >6-fold cutoff was chosen with the goal of highlighting those genes to be of the most biological significance, and the complete list of differentially expressed genes >2-fold are listed in Supplemental Tables S5, S6, S7, S8, S9, S10 by locus tag number. Several trends of note, including cellular attributes such as motility, cell structure, and genes putatively involved in pathogenic potential, are discussed.

**Table 5:** Chromosomal genes with increased expression (≥6-fold) for three *Bacillus* strains in MGM+CO_2_+0.8% bicarbonate for functional families: Energy & Metabolism; Spore Function; and Cellular & Enzymatic Activity.

Gene name	*locus #	COG, GO, Pfam	Fold Difference
**ENERGY & METABOLISM**			
***B. cereus*** ** G9241**			
PTS system IIA component	0778	PTS System	6.19
*fabH*; 3-oxoacyl-(acyl-carrier-protein) synthase III subfamily	1828	Fatty acid and phospholipid metabolism	6.89
*appC*; cytochrome d ubiquinol oxidase subunit I	1951	oxidoreductase activity and electron transport	13.42
*cydB*; cytochrome d ubiquinol oxidase subunit_II	1952	oxidoreductase activity and electron transport	17.33
acetyltransferase	2556	acetyltransferase activity	7.27
streptogramin A acetyl transferase	2581	acetyltransferase activity	6.27
*acd-7*; acyl-CoA dehydrogenase	5102	Fatty acid and phospholipid metabolism	7.44
*atoB-4*; acetyl-CoA acetyltransferase	5104	acetyl-CoA C-acetyltransferase activity	9.05
***B. anthracis*** ** Sterne 34F_2_**			
acyl-CoA synthase	1091	fatty acid metabolism	7.24
glycolate oxidase, iron-sulfur subunit, putative	1308	carbohydrate metabolism	9.21
*glcD*; glycolate oxidase, subunit glcd	1309	carbohydrate metabolism	9.72
*cydB-1*; cytochrome d ubiquinol oxidase, subunit ii	1944	oxidoreductase activity and electron transport	36.62
alcohol dehydrogenase	2267	fermentation	7.62
acetyltransferase, gnat family	2534	acetyltransferase activity	9.53
***B. cereus*** ** 10987**			
cytochrome d ubiquinol oxidase, subunit II	4950	oxidoreductase activity and electron transport	7.47
acyl-CoA dehydrogenase	5142	oxidoreductase activity and electron transport	12.47
acetyl-CoA acetyltransferase	5143	acetyltransferase activity	10.16
3-hydroxyacyl-CoA dehydrogenase/enoyl-CoA hydratase/isomerase family protein	5144	oxidoreductase activity and electron transport	11.77
**SPORE FUNCTION**			
***B. anthracis*** ** Sterne 34F_2_**			
germination protein gerN	1639	antiporter activity, sporulation (sensu Bacteria)	11.70
spore coat protein, putative	2536	sporulation (sensu Bacteria)	6.62
***B. cereus*** ** 10987**			
germination protein gerN	1729	antiporter activity, sporulation (sensu Bacteria)	14.02
**CELLULAR & ENZYMATIC ACTIVITY**			
***B. cereus*** ** G9241**			
*arcB*; ornithine cyclodeaminase	0906	catalytic activity	6.08
penicillin-binding protein	2201	beta-lactamase activity	28.62
yersiniabactin synthetase salycilate ligase component	2335	siderophore biosynthesis	11.63
reticulocyte binding protein	2337	Biosynthesis of cofactors, prosthetic groups, and carriers	6.86
*ribH*; 6, 7-dimethyl-8-ribityllumazine synthase	4120	Biosynthesis of cofactors, prosthetic groups, and carriers	6.71
transposase X	4400	transposase activity (homolog on pXO1)	7.66
*hbd-1*; putative 3-hydroxyacyl-CoA dehydrogenase FadB	5105	catalytic activity	12.11
***B. anthracis*** ** Sterne 34F_2_**			
*eag*: S-layer protein ea1	0887	S-layer	21.25
S-layer protein, putative	1130	S-layer	6.54
acetoacetyl-coa synthase, putative	2553	ligase activity, forming carbon-sulfur bonds	6.73
copper-ion-binding protein	3860	copper ion binding	10.70
sulfatase	3895	sulfuric ester hydrolase activity	7.85

* Locus tag numbers are from the *B. cereus* G9241 (BCE_G9241_XXXX), *B. anthracis* Ames Ancestor (GBAAXXXX) and the *B. cereus* 10987 (BCE_XXXX) genomes.

**Table 6:** Chromosomal genes with increased expression (≥6-fold) for three *Bacillus* strains in MGM+CO_2_+0.8% bicarbonate for functional families: Amino Acids; Nucleic Acids; and Transport.

Gene name	*locus #	COG, GO, Pfam	Fold Difference
**AMINO ACIDS**			
***B. cereus*** ** G9241**			
*kamA*; L-lysine 2,3-aminomutase	2268	L-lysine catabolism	26.24
*hutI*; imidazolonepropionase	3605	histidine catabolism	7.65
*hutU*; urocanate hydratase	3606	histidine catabolism	6.26
***B. anthracis*** ** Sterne 34F_2_**			
*kamA*; l-lysine 2,3-aminomutase	2300	L-lysine catabolism	23.71
***B. cereus*** ** 10987**			
formimidoylglutamase	3677	histidine catabolism	6.40
**NUCLEIC ACIDS**			
***B. cereus*** ** G9241**			
*nrdG*; anaerobic_ribonucleoside-triphosphate reductase activating protein	3554	ribonucleoside-triphosphate reductase activity	10.01
anaerobic ribonucleoside-triphosphate reductase,	3555	ribonucleoside-triphosphate reductase	8.06
***B. anthracis*** ** Sterne 34F_2_**			
anaerobic ribonucleoside triphosphate reductase	3663	ribonucleoside-triphosphate reductase	11.83
***B. cereus*** ** 10987**			
anaerobic ribonucleoside triphosphate reductase	3622	ribonucleoside-triphosphate reductase	8.40
carbamoyl-phosphate synthase large subunit	3931	pyrimidine ribonucleotide biosynthesis	6.47
carbamoyl-phosphate synthase small subunit	3932	pyrimidine ribonucleotide biosynthesis	6.13
dihydroorotase	3933	pyrimidine ribonucleotide biosynthesis	6.38
**TRANSPORT (General)**			
***B. cereus*** ** G9241**			
cation-transporting ATPase E1-E2 family	0415	Cation transport	8.04
ABC transporter ATP-binding protein CydD	1953	ATPase activity, coupled to transmembrane movement of substances	29.09
ABC transporter ATP-binding protein CydC	1954	ATPase activity, coupled to transmembrane movement of substances	45.53
drug transporter, putative	2339	multidrug transport	8.91
sulfate permease family protein VCA0077	2987	multidrug transport	8.76
*pacS*; cation-transporting ATPase P-type	3696	Cation transport	6.80
phosphoglycerate transporter protein	5423	phosphoglycerate transport	7.31
***B. anthracis*** ** Sterne 34F_2_**			
oligopeptide abc transporter, oligopeptide-binding protein, putative	0231	oligopeptide transport	6.89
cation-transporting atpase, e1-e2 family	0405	cation transport	8.06
phosphate abc transporter, phosphate-binding protein, putative	0715	phosphate transport	16.41
phosphate abc transporter, permease protein, putative	0716	phosphate transport	9.92
phosphate abc transporter, permease protein, putative	0717	phosphate transport	9.96
formate/nitrite transporter family protein	1321	Nitrite/formate transport	6.91
transport atp-binding protein cydc	1945	ATPase activity, coupled to transmembrane movement of substances	65.50
transport atp-binding protein cydd	1946	ATPase activity, coupled to transmembrane movement of substances	24.51
heavy metal-transporting atpase	3859	Cation transport	6.55
*pstB*; phosphate abc transporter, atp-binding protein	4493	phosphate transport	13.04
*pstA*; phosphate abc transporter, permease protein	4494	phosphate transport	18.28
*pstC*; phosphate abc transporter, permease protein	4495	phosphate transport	18.35
*phoX*; phosphate abc transporter, phosphate-binding protein	4496	phosphate transport	24.10
***B. cereus*** ** 10987**			
cation-transporting ATPase, E1-E2 family	0519	Cation transport	8.46
citrate cation symporter family	0641	Cation transport	8.28

* Locus tag numbers are from the *B. cereus* G9241 (BCE_G9241_XXXX), *B. anthracis* Ames Ancestor (GBAAXXXX) and the *B. cereus* 10987 (BCE_XXXX) genomes.

**Table 7:** Chromosomal genes with increased expression (≥6-fold) for three *Bacillus* strains in MGM with normal aeration (O_2_) for functional families: Energy & Metabolism and Cellular & Enzymatic Activity.

Gene name	*locus #	COG, GO, Pfam	Fold Difference
**ENERGY & METABOLISM**			
***B. cereus*** ** G9241**			
*treP*; PTS system trehalose-specific IIBC component	0608	trehalose transport	84.83
trehalose-6-phosphate hydrolase	0609	trehalose transport	47.16
*glpT*; glycerol-3-phosphate transporter	0644	glycerol transport	26.69
NADP-dependent glyceraldehyde-3-phosphate dehydrogenase	0851	energy metabolism	6.05
*glpD*; aerobic glycerol-3-phosphate dehydrogenase	1045	glycerol transport	9.80
*malS*; malate oxidoreductase VC1188	1806	malate metabolism	15.88
*fruA*; PTS system fructose-specific family IIABC components	3681	PTS System	33.25
*fruB*; 1-phosphofructokinase	3682	glycolysis	18.73
*ptsG*; PTS system glucose-specific IIABC component	4046	PTS System	8.34
acetyltransferase CysE/LacA/LpxA/NodL family	4749	acetyltransferase	6.59
*ndh*; NADH dehydrogenase	5020	oxidoreductase activity	6.03
6-phospho-beta-glucosidase	5345	carbohydrate catabolism	29.65
PTS system IIA component	5346	PTS System	8.81
*celA*; PTS system IIB component	5348	PTS System	16.48
glycerol uptake facilitator protein	CNI_0291	glycerol transport	21.31
*glpK*; glycerol kinase	CNI_0292	glycerol metabolism	11.05
***B. anthracis*** ** Sterne 34F_2_**			
acetyltransferase, gnat family	0587	N-acetyltransferase activity	8.85
*treB*; pts system, trehalose-specific iibc component	0631	trehalose transport	20.61
*treC*; trehalose-6-phosphate hydrolase	0632	trehalose catabolism	26.81
*glpT*; glycerol-3-phosphate transporter	0661	glycerol-3-phosphate transport	16.60
*glpF*; glycerol uptake facilitator protein	1025	glycerol transport	26.23
*glpK*; glycerol kinase	1026	glycerol metabolism	17.44
*glpD*; glycerol-3-phosphate dehydrogenase, aerobic	1027	glycerol metabolism	64.69
malate dehydrogenase, putative	3145	malate metabolism	18.64
***B. cereus*** ** 10987**			
glycolate oxidase, iron-sulfur subunit, putative	1409	oxidoreductase activity	15.08
glycolate oxidase, subunit GlcD	1410	oxidoreductase activity	21.41
**CELLULAR & ENZYMATIC ACTIVITY**			
***B. cereus*** ** G9241**			
microbial collagenase	0535	proteolysis and peptidolysis	63.51
proteinase VCA0223	0654	metalloendopeptidase activity	212.16
S-layer homology domain	0996	Cell envelope	28.32
membrane protein putative	1875	Cell envelope	9.61
MutT/nudix family protein putative	2994	catalytic activity	10.85
glycerophosphoryl diester phosphodiesterase	3442	phospholipid catabolism	6.15
phosphatidylinositol-specific phospholipase C X domain protein	3732	phospholipid catabolism	31.51
microbial collagenase	3733	proteolysis and peptidolysis	16.06
S-layer homology domain	4903	S-Layer	9.51
endonuclease/exonuclease/phosphatase family protein putative	5186	catalytic activity	6.57
LrgA family protein	5628	peptidoglycan catabolism	43.01
proteinase VCA0223	CNI_0273	metalloendopeptidase activity	31.63
***B. anthracis*** ** Sterne 34F_2_**			
glycerophosphoryl diester phosphodiesterase, putative	3560	phospholipid catabolism	40.91
***B. cereus*** ** 10987**			
murein hydrolase regulator LrgA	5572	peptidoglycan catabolism	13.05

* Locus tag numbers are from the *B. cereus* G9241 (BCE_G9241_XXXX), *B. anthracis* Ames Ancestor (GBAAXXXX) and the *B. cereus* 10987 (BCE_XXXX) genomes.

**Table 8:** Chromosomal genes with increased expression (≥6-fold) for three *Bacillus* strains in MGM with normal aeration (O_2_) for functional families: Amino Acids; Nucleic Acids; and Transport.

Gene name	*locus #	COG, GO, Pfam	Fold Difference
**AMINO ACIDS**			
***B. cereus*** ** G9241**			
*gltT*; proton/sodium-glutamate_ ymport protein	1803	L-glutamate transport	46.21
*aspA*; aspartate ammonia-lyase	1804	amino acid metabolism	24.83
*aspA*; aspartate ammonia-lyase	1805	amino acid metabolism	8.88
*ilvE*; branched-chain amino acid aminotransferase	1847	amino acid metabolism	25.27
*ilvB*; acetolactate synthase large subunit biosynthetic type	1848	amino acid metabolism	22.92
*ilvN*; acetolactate synthase III small chain VC2482	1849	amino acid metabolism	20.06
*ilvC*; ketol-acid reductoisomerase	1850	amino acid metabolism	23.77
*ilv*D;dihydroxy-acid dehydratase	1851	amino acid metabolism	20.56
*ilvA*; threonine dehydratase	1852	amino acid metabolism	7.02
*hisC*; histidinol-phosphate aminotransferase	2916	amino acid biosynthesis	7.11
*aspA*; aspartate ammonia-lyase	3066	amino acid metabolism	13.22
*argG*; argininosuccinate synthase	4713	amino acid biosynthesis	9.10
***B. anthracis*** ** Sterne 34F_2_**			
*trpE*; anthranilate synthase component I	1248	tryptophan biosynthesis	6.34
proton/sodium-glutamate symporter	1799	L-glutamate transport	13.04
*aspA-2*; aspartate ammonia-lyase	1800	amino acid metabolism	7.03
*aspA-3*; aspartate ammonia-lyase	3136	amino acid metabolism	6.85
*brnQ-5*; branched-chain amino acid transport system ii carrier protein	3142	branched-chain aliphatic amino acid transport	9.35
*proC*; pyrroline-5-carboxylate reductase	3143	proline biosynthesis	14.23
*glsA-2*; glutaminase	3155	amino acid and derivative metabolism	16.03
*brnQ-6*; branched-chain amino acid transport system ii carrier protein	4790	branched-chain aliphatic amino acid transport	10.55
sodium/alanine symporter family protein	5301	L-alanine transport	12.98
**NUCLEIC ACIDS**			
***B. cereus*** ** G9241**			
extracellular ribonuclease	3260	RNA catabolism	12.99
pyrimidine nucleoside trans	5169	nucleoside transport	57.48
nupC family protein	5398	nucleoside transport	7.33
***B. cereus*** ** 10987**			
nucleoside transporter, NupC family	5354	nucleoside transport	14.05
**TRANSPORT (General)**			
***B. cereus*** ** G9241**			
daunorubicin resistance ATP-binding protein drrA	0485	Transport and binding proteins	6.30
di-/tripeptide transporter	0670	Transport and binding proteins	6.50
transporter LysE family	1880	Transport and binding proteins	19.00
oxalate/formate antiporter putative	2332	formate transporter activity	19.88
ABC transporter ATP-binding protein	2998	Transport and binding proteins	6.50
sodium∶alanine symporter family protein	3091	Transport and binding proteins	8.13
*sugE*; SugE protein	4239	Transport and binding proteins	10.06
***B. anthracis*** ** Sterne 34F_2_**			
oxalate∶formate antiporter, putative	2367	formate transporter activity	77.66
major facilitator family transporter	3020	Transport and binding proteins	6.81
***B. cereus*** ** 10987**			
oxalate∶formate antiporter, putative	2396	formate transporter activity	78.18

* Locus tag numbers are from the *B. cereus* G9241 (BCE_G9241_XXXX), *B. anthracis* Ames Ancestor (GBAAXXXX) and the *B. cereus* 10987 (BCE_XXXX) genomes.

**Table 9:** Chromosomal genes with increased expression (≥6-fold) for three *Bacillus* strains in MGM with normal aeration (O_2_) for functional families: Regulation; Motility & Chemotaxis; and Pathogenesis & Toxins.

Gene name	*locus #	COG, GO, Pfam	Fold Difference
**REGULATION**			
***B. cereus*** ** G9241**			
transcriptional activator NprR	0573	Regulatory functions	6.23
transcriptional regulator MarR family	0645	Regulatory functions	8.47
*cheA*; histidine kinase (cheA)	1671	Signal transduction	6.42
sensor histidine kinase putative	1807	Signal transduction	16.00
response regulator putative	1808	Signal transduction	10.42
*fruR*; transcriptional regulator DeoR family	3683	Regulatory functions	13.14
response regulator putative	5603	Signal transduction	6.74
***B. anthracis*** ** Sterne 34F_2_**			
transcription antiterminator, lytr family	3647	transcription antiterminator activity	6.79
rna polymerase sigma-70 factor, ecf subfamily	3649	sigma factor activity	9.15
**MOTILITY & CHEMOTAXIS**			
***B. cereus*** ** G9241**			
*flgB*; flagellar basal-body rod protein flgB	1684	motility	12.05
*flgC*; flagellar basal-body rod protein flgC	1685	motility	9.78
flagellar hook-basal body complex protein *fliE*	1686	motility	15.92
flagellar M-ring protein putative	1687	motility	12.91
*fliG*; flagellar motor switch protein fliG	1688	motility	6.45
Basal-body rod modification protein flgD	1693	motility	6.29
flagellar hook protein flgE putative	1694	motility	9.79
flagellin	1701	motility	7.19
flagellin	1702	motility	7.47
flagellin	1703	motility	9.02
flagellar motor switch protein fliN VC2125 putative	1705	motility	6.41
flagellar motor switch protein (fliM) putative	1706	motility	6.41
flagellar motor switch protein fliN	1708	motility	7.29
*fliP*; flagellar biosynthetic protein fliP	1709	motility	6.48
flagellar biosynthetic protein fliR putative	1711	motility	7.96
*flhB*; flagellar biosynthesis protein flhB	1712	motility	7.11
flagellar biosynthetic protein fliR putative	CNI_0304	motility	8.58
*cheY*; chemotaxis response regulator	1670	motility	6.32
**PATHOGENESIS & TOXINS**			
***B. cereus*** ** G9241**			
**phospholipase c precursor	0658	hemolysis	200.54
** *AA1-330*; phospholipase c precursor	0659	hemolysis	150.93
**non-hemolytic enterotoxin lytic component L2	1876	toxin	39.16
**enterotoxin A	1877	toxin	61.32
**non-hemolytic enterotoxin lytic component L1	1878	toxin	28.18
**non-expressed enterotoxin C	1879	toxin	58.60
**bacillolysin	2676	proteolysis and peptidolysis	86.91
hemolysin BL binding component precursor	3074	hemolysis	28.70
**Hbl B protein	3075	hemolysis	129.93
**hemolysin BL lytic component L1	3076	hemolysis	52.52
**hemolysin BL lytic component L2	3077	hemolysis	33.91
**perfringolysin O precursor	3245	cytolysin	110.36

* Locus tag numbers are from the *B. cereus* G9241 (BCE_G9241_XXXX), *B. anthracis* Ames Ancestor (GBAAXXXX) and the *B. cereus* 10987 (BCE_XXXX) genomes.

** Toxin Homologs >90% exist in *B. cereus* 14579 and were shown to be members of the PlcR regulon [49].

**Figure 4:**
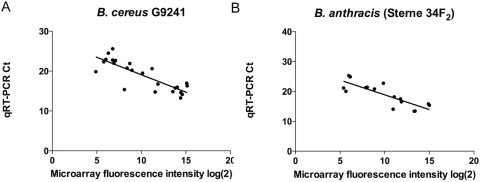
Spearman correlations comparing microarray and SYBR-Green qRT-PCR gene expression results for select genes in two conditions - CO_2_ and O_2_. (A) *B. cereus* G9241; expression for 12 genes in 2 conditions (5 chromosomal, 4 from pBC218, and 3 from pBCXO1); Spearman r = −0.7797; p<0.0001; and (B) *B. anthracis* Sterne 34F_2_; 8 genes in two conditions (5 chromosomal, 3 from pXO1); Spearman r = −0.7735; p = 0.0004. Genes and primer sequences listed in Tables S11 and S12.

Because our array data showed that the two pathogens, *B. anthracis* and G9241, exhibited such pronounced differential expression patterns between the two conditions (often >9–200 fold difference), we performed qRT-PCR on a select set of genes for these two strains to verify that the trends we had observed were reproducible by a complementary approach. The genes assayed were from both chromosomal and plasmid loci, and represent both up- and down-regulated genes between the two growth conditions (see Tables S11 and S12 for gene names and primer sequences). These results are shown in Figure 4, and clearly display good correlation between the two assays (Pearson r = −0.7735 for *B. anthracis* and −0.7757 for G9241, p<0.0004 for both).

### Motility genes

*B. anthracis* is non-motile, but retains a set of motility genes, some of which contain disrupting mutations [18], [33]. *B. cereus* strains exhibit a range of motility behavior [3], and both G9241 and 10987 displayed observable motility by microscopy when grown in rich broth. G9241 showed conspicuously increased expression of motility genes during growth in O_2_ (Table 9), including those encoding flagellar components and chemotaxis sensory genes (18 genes >6-fold; 19 genes between 2–5 fold (Table S13 summarizes differentially expressed motility genes for G9241 and *B. anthracis*)). This substantial trend suggested that motility may be reduced in MGM in the CO_2_ environment for G9241, and we confirmed this trait via microscopy of cells from liquid cultures. Because microscopic examination is qualitative, we also used *B. subtilis* as a baseline model for robust motility. Both *B. cereus* 10987 and *B. subtilis* 168 maintained motility in the CO_2_/bicarbonate environment as observed by microscopy, but were qualitatively more motile in the O_2_ non-bicarbonate environment. G9241 did not appear motile in CO_2_, but was quite motile in MGM in full aeration, as well as in rich LB medium.

For *B. anthracis*, 7 methyl-accepting chemotaxis protein (MCP) genes and 1 putative flagellar motor hook gene were increased in expression in O_2_ between 2–5 fold (Table S13). Of these 8 genes, 5 are included in the list of genes conserved and differentially expressed commonly with G9241 (Table S2). Therefore, even though *B. anthracis* has not been reported to be motile in any previous study (and was negative for observable motility in all media and atmospheres tested in this study) we see that various genes implicated in motility have been maintained and are expressed in a similar fashion to a fully motile relative, perhaps suggesting that these chemo-sensors play roles in traits other than motility. One of these MCP genes that is present in both *B. anthracis* and G9241 (GBAA5317 homologous to G9241_5185) may be expressed as part of a two-gene operon, since their homologous upstream neighbors (GBAA5318 and G9241_5186: putative endonuclease/exonuclease/phosphatase family protein) were also more highly expressed in O_2_ for both strains, and there is a very small (25 nt) intergenic region. Transcriptome sequencing data from a different project support this observation (Passalacqua et al., unpublished), where these two genes for *B. anthracis* are apparently transcribed on a contiguous mRNA unit. BLASTp analysis of this putative endonuclease in the JGI IMG (http://img.jgi.doe.gov/cgi-bin/pub/main.cgi) shows homologs present in the pathogenic *Bacillus cereus* sensu lato group >90% (*B. anthracis*, *B. cereus*, *B. thuringiensis*), but identities quickly drop off for microbes in the rest of this database, implicating a specific role for these genes in this group of bacteria.

### S-Layer genes

S-layers are protective cell coats that are involved in a variety of bacterial characteristics, such as cell adhesion, permeability layers, and protein scaffolds, and multiple genes in many prokaryotic genomes are implicated in S-layer structure and affiliation (see Review [34]). In *B. anthracis*, the chromosomal genes for the S-layer proteins EA1 and Sap have been previously characterized and were shown to be the main components of the *B. anthracis* S-layer [35]. Also, a pXO1-encoded S-layer protein (*BslA*) was recently shown to play a role in the ability of *B. anthracis* vegetative cells to adhere to host cells [36]. We observed that the *B. anthracis* chromosomal EA1 (GBAA0887) gene and the pXO1 encoded gene *BslA* (pXO1_0124) were both significantly more highly expressed in CO_2_ (21 and 35 fold respectively, Tables 5 and 3) (all putative S-layer genes differentially expressed in CO_2_ and O_2_ for all three *Bacillus* strains are listed together in Table S14). However, no transcriptional differences were observed in *B. anthracis* for Sap, and both microarray fluorescence intensities and transcriptome sequencing data suggest that this gene is constitutively expressed at a substantial level in both conditions (Passalacqua et al., unpublished). Of 11 other putative S-layer proteins on the *B. anthracis* chromosome, one had higher expression in CO_2_ (GBAA1130, 6-fold) and three were more highly expressed in O_2_ (GBAA0981, 1926, and 2315, up 3, 5 and 2.4-fold in O_2_ respectively; Table S14). These data suggest that in addition to the important Sap component of the *B. anthracis* S-layer, this microbe expresses other S-layer components differentially, likely due to growth condition-specific needs.

The differential expression of the G9241 putative S-layer genes showed both similarities to and differences from the pattern in *B. anthracis*. First, the G9241 homolog of *BslA*, located on the pBCXO1 plasmid (pBCXO1_0105), was, as in *B. anthracis*, increased substantially in CO_2_ (12 fold) (Tables 3 and S14). However, for the two G9241 chromosomal S-layer homologs of EA1 and Sap, the G9241 EA1 gene showed no difference in transcript abundance in CO_2_ and Sap was ∼3 fold more highly expressed in O_2_. The G9241 S-layer gene locus 1115 (homolog to *B. anthracis* locus 1130) was increased in CO_2_ as in *B. anthracis* (∼2.5 fold each - 96% protein identity) (Table S14). Also, the chromosomal homolog to one of the *B. anthracis* genes more highly expressed in O_2_ showed increased expression in G9241, but to a substantially larger degree (Tables 7 and S14) (G9241 locus 0996 up 28 fold versus 3 fold for *B. anthracis* locus 0981 (95% protein identity)). One last chromosomal S-layer gene for G9241 showed higher expression in O_2_ (locus 4903 up 9.5 fold) (Table 7), and this gene shares ∼96% protein identity with the *B. anthracis* locus 5054 and the 10987 locus 4952. The 10987 homolog was the only putative S-layer protein to be differentially expressed in this strain (up ∼2 fold in O_2_), whereas the *B. anthracis* version did not show differential expression.

Two putative S-layer genes on the pBC218 plasmid of G9241 were highly upregulated in CO_2_, by 28 and 41 fold (pBC218_0012 and 0043, respectively) (Tables 2 and S14). The former gene is a homolog to the *B. anthracis* pXO2-encoded S-layer protein, which would not be detected in these experiments for *B. anthracis* due to the lack of pXO2 in the Sterne strain. The latter gene does not have a closely related homolog in either *B. anthracis* or in 10987, and may represent a unique S-layer component in G9241, since a protein BLAST analysis of this gene with a threshold of >90% homology against all microbial genomes in the JGI IMG database resulted in no hits, not even in the numerous other sequenced *Bacillus* genomes. The differential expression patterns of S-layer genes between CO_2_ and O_2_ conditions suggest that *B. anthracis* evolved a regulatory pattern for the EA1 S-layer component unique from both *B. cereus* strains and that the pathogenic *Bacillus* strains may adapt unique S-layer structures differentially in response to various growth conditions, contributing to the phenotypic uniqueness of each strain.

### Pathogenesis genes

Anthrax toxin has been an active topic of research, and control of toxin gene expression in *B. anthracis* in CO_2_/bicarbonate by the important regulator AtxA has been well-studied [11], [24], [37]–[39]. The anthrax toxin genes and the AtxA regulator are encoded on the *B. anthracis* pXO1 virulence plasmid, but this regulator also controls the expression of various chromosomal genes [11]. Regarding toxin expression profiles for *B. anthracis* in this study, we note here mainly that our results match previous work in showing increased expression of the key genes involved in toxin production in CO_2_/bicarbonate growth (*lef*, *pagA*, *cyaA*, and *atxA*; pXO1_0172, 0164, 0142, and 0146 respectively) (Table 3 and Table S7). Recently, it has been shown that a particular bicarbonate transporter (*B. anthracis* Sterne loci BAS2712-14, orthologous to Ames Ancestor GBAA2920-22) is important for *B. anthracis* virulence [16]. However, these transporter genes were not differentially expressed between CO_2_ and O_2_; perhaps they are constitutive, which seems consistent with a critical role in rapid assimilation to a bicarbonate-rich environment.

Outside of the well-characterized determinants in *B. anthracis* (i.e., toxins), what is considered a virulence factor in *B. anthracis* is a matter of interpretation. We simply note that the scope of this study for *B. anthracis* was to attain a global view of transcriptional activity between CO_2_/bicarbonate and O_2_, and so we direct the reader to the various tables for more gene-specific details. Regarding G9241, however, a primary goal of this study was to elucidate this unusual pathogen's transcriptional behavior in both a global and specific sense, and in terms of putative virulence determinants, this microorganism displays a high level of CO_2_ versus O_2_ specific transcriptional regulation.

*B. anthracis* and G9241 harbor two operons putatively encoding siderophore biosynthetic genes for iron acquisition: the petrobactin operon (*B. anthracis* loci GBAA1981-86; G9241 loci BCE_G9241_1997-2002), and the *dhb* operon (*B. anthracis* loci GBAA2368-72; G9241 2333-37). Strain 10987 only harbors the *dhb* operon, which is highly conserved in *Bacilli* (BCE_2398-2402) [40]. The petrobactin operon has been implicated in the virulence of *B. anthracis* [41], and we have observed that expression of this operon for *B. anthracis* during exponential growth is constitutive (KDP, unpublished), whereas the *dhb* operon of *B. anthracis* is tightly regulated by iron (KDP, unpublished), akin to that seen in *B. subtilis* [42]. For G9241, several genes from both operons were more highly transcribed in the CO_2_ environment (Table S5): *dhb* genes up ∼3–11 fold; petrobactin genes up ∼3–5 fold). Regarding the petrobactin operon, it does not appear that iron itself is the key regulatory factor for this operon in *B. anthracis*, and thus, we only note that the 100 bp upstream intergenic sequences where putative regulatory sequences most likely are located are slightly divergent between G9241 and *B. anthracis* (4 differences of 1–2 nt each), and may be contributing to the small differential expression for G9241. More interestingly, the 100 bp upstream nucleotide sequences of the *dhb* operon for *B. anthracis* and 10987 differ by only 2 single nucleotides, with exact matching Fur iron regulatory protein consensus sequences located 65 base pairs upstream of the translational start sites [43], whereas the canonical Fur binding sequence in G9241 is located 75 bp upstream of the translational start site. Because changes in regulatory regions are a major force in the evolution of genetic regulation [44], further analysis of these important sequences should shed much light on the evolution of gene regulation in bacteria.

*B. cereus* strains are very diverse in the specific toxin genes that they harbor, and these toxins have been implicated in different types of food-borne illnesses [45]–[48]. Here, G9241 displayed very strong induction of multiple chromosomal enteroxin, hemolysin, and phospholipase genes in O_2_ (33–200 fold differences), the homologs of which have been shown previously to be regulated by the transcriptional regulator PlcR in *B. cereus* strain 14579 during stationary phase [49] (19 out of 27 G9241 genes sharing >85% protein identity with strain 14589 plcR regulated genes in [49]) (Table 9 - Pathogenesis & Toxins, PlcR regulated homologs marked with **). Hence, in our study, it seems that putative PlcR-regulated virulence determinants were strongly correlated with growth in an aerobic environment. This is in agreement with previous observations that cereulide toxin production in *B. cereus* is influenced by the presence of atmospheric O_2_ [50]. The G9241 *plcR* gene itself (locus 5525, ∼72% protein identity to *B. cereus* 14579 PlcR) is ∼2.4 fold more highly expressed in O_2_ as well (Table S6). Homologs of many of these toxin genes exist in the genomes of *B. anthracis* and 10987, but do not show differential expression in O_2_ for these strains. Interestingly, three additional, putative enterotoxin genes are found on G9241's pBC218 plasmid (pBC218_0039, 0040 and 0042) (Table 2) and, unlike the chromosomally encoded toxins, they are substantially more highly expressed in CO_2_ (42, 75 and 24 fold difference, respectively). These genes do not share protein identity >41% with any bacteria in the JGI IMG database, so it appears that G9241 has evolved various regulatory strategies for unique toxin expression in each growth condition. The differential expression of various enterotoxin genes has been observed in different *B. cereus* under a variety of conditions, including anaerobic growth and availability of specific nutrients [51], [52]. Given, however, that G9241 has only been seen to cause a pneumonia-like disease [5], and has not been demonstrated to cause food-borne illness like many of the other *B. cereus* strains, it is not clear what role enterotoxins or their differential regulation may play in the pathogenesis of this strain.

In *B. anthracis*, the *plcR* gene contains a nonsense mutation that prevents the protein from being fully translated [53]. However, as mentioned, *B. anthracis* has the AtxA regulator, whose gene is located on the pXO1 plasmid, and it controls both pXO1 virulence determinants and chromosomal genes [11]. We observed that the *atxA* gene and the toxin genes it regulates in *B. anthracis* were more highly expressed in CO_2_ for this bacterium (Table 3). It has been proposed that a certain incompatibility may exist between PlcR and AtxA regulons [54], particularly in regard to sporulation. In G9241, however, the situation appears to be somewhat complex, where two homologs to the *B. anthracis atxA* gene exist and an active PlcR regulator appears to function (as inferred from the upregulation of various toxin genes; see previous). In looking at this situation in more detail, we noted that gene locus pBCXO1_0126 in G9241 is currently annotated as a “conserved hypothetical protein”. However, the nucleotide sequence of this gene (1428 bp) is 100% identical to the *B. anthracis* pXO1_0146 *atxA* gene, indicating an error in the pBCXO1 annotation. As in *B. anthracis*, this G9241 gene that we now consider to be the *atxA* gene showed higher expression in CO_2_ by ∼5.6 fold (Table 3). A less conserved gene on the pBC218 plasmid (locus 0050) is annotated as a “trans-acting activator”, and it shares ∼79% protein identity with *B. anthracis atxA*, but it was not differentially expressed between CO_2_ and O_2_. Lastly a small gene on G9241's pBC218 plasmid (locus 0049) is annotated as a “trans-acting positive regulator AtxA”; however, it is only 420 bp compared to 1428 bp full length *B. anthracis atxA* gene, and it actually shares ∼81% protein identity with the *B. anthracis* pXO1_0148 hypothetical protein; therefore, it is unclear what relation it bears to the *B. anthracis* AtxA regulator, if any. The G9241 pBCXO1 loci 0105 and 0106 are increased in expression in CO_2_ (∼13 and 10-fold, respectively), and these genes are homologous to the *B. anthracis* pXO1 loci 0124 and 0125 which have been shown to be strongly induced by AtxA [11] (Table 3). Therefore, upon closer inspection, we see that G9241 likely has both an active PlcR-like regulator and an AtxA-like regulator, which are themselves differentially expressed between O_2_ and CO_2_. This suggests that any incompatibility between the two regulators may be avoided in G9241 by this microbe's specific utilization of each regulator during unique growth environments.

### Conclusions and future directions

Global transcriptional analyses via microarrays provide wonderful genome-scale views of gene expression, and inevitably supply multiple avenues for more detailed, gene-specific studies. Here, we chose to explore global gene expression patterns in three closely related but phenotypically unique *Bacillus* strains in two very different atmospheric growth conditions (CO_2_/bicarbonate versus O_2_). The data presented highlight both conservation and divergence amongst the three *Bacilli*, and in particular, revealed a high level of atmosphere-dependent genetic regulation for the two pathogenic strains (*B. cereus* G9241 and *B. anthracis*). A set of genes involved in general metabolic processes showed a conserved pattern of differential expression in all three strains between the two growth conditions, and mutagenesis experiments might reveal how vital these genes are for the genus *Bacillus* as a whole. Conversely, genes involved in motility, S-layer structures, and pathogenesis appeared to be expressed in a regulated manner only in the pathogens, suggesting that these microbes are able to modify their metabolic and structural features in a more profound way in response to the environment. In particular, transcription of genes located on virulence plasmids for G9241 and *B. anthracis* only showed increased expression in the CO_2_ environment, while the one plasmid of *B. cereus* 10987, despite being ancestrally derived from the pXO1 plasmid family, did not show any regulatory, transcriptional changes between the two conditions. Interestingly, G9241 did not display motility when grown planktonically in CO_2_/bicarbonate, but rather appeared to be upregulating capsule production in this putatively host-like environment. The most unusual finding was that, unlike *B. anthracis*, G9241 may utilize both PlcR and AtxA regulators, but each in a different growth condition. Gene-specific mutagenesis and biochemical strategies will surely continue to elucidate the complex regulatory repertoires of these interesting bacteria.

## Materials and Methods

### Bacterial strains and culture conditions for RNA isolation

Strains used in this study are *Bacillus anthracis* Sterne (34F_2_); *Bacillus cereus* G9241 (acquired from the Centers for Disease Control (CDC), Atlanta, GA); and *Bacillus cereus* 10987 (acquired from the American Type Culture Collection (ATCC)). Overnight cultures were started by picking a fresh colony from a TSA blood agar plate (*B. anthracis* was negative for hemolysis, and *B. cereus* strains showed positive hemolysis) and inoculating 5 ml of Luria-Bertani (LB) medium and grown at 37°C with shaking at 150 rpm. Overnight cultures were back-diluted in the morning to an optical density (600 nm) (OD_600_) of 0.1 in 5 ml of LB medium and allowed to recover to OD_600_ 0.3. These cultures were then used to inoculate 60 ml of Modified G Medium (MGM) (recipe available at http://bergmanlab.biology.gatech.edu/), or 60 ml of MGM+0.8% sodium bicarbonate (NaHCO_3_^−^), to an OD_600_ of 0.01, in 500 ml volume erlenmeyer flasks. Cells were grown to mid-exponential phase (OD_600_ 0.4–0.5) at 37°C with shaking at 250 rpm. Cells were grown in either ambient air (MGM only) or in the presence of 14–15% CO_2_ (MGM+0.8% NaHCO_3_^−^). All flasks were sealed with “Bugstopper” vented plugs (Fisher Scientific) to allow gas exchange. All experiments were performed such that 4 biological replicates were collected for each strain in each condition.

### RNA Isolation and cDNA Synthesis

RNA collection was done essentially as described previously [9], [55]. Briefly, when cells reached the target OD_600_, they were filtered in sterile 150 ml Nalgene 0.2 µm filter flasks, immediately resuspended in 13 ml RNase/DNase-free sterile water and moved to a sterile 50 ml RNase/DNase-free centrifuge tubes (Corning 4558). For *B. cereus* G9241 grown in the presence of CO_2_, cells could not be filtered due to capsule expression (filters were clogged quickly), so they were spun by centrifugation at top speed for 3 minutes, after which supernatants were poured off. Boiling lysis buffer (6.5 ml total: 0.2% SDS, 16 mM EDTA, 200 mM NaCl) was added and the tubes were incubated at 100°C for 5 minutes. The time between cell filtering, centrifugation and suspension in boiling lysis buffer was <5 minutes. Note that in previous studies we directly compared mRNA collected by centrifugation and by filtration and found no differences in the two methods (N. Bergman and E. Anderson, unpublished). We also found no differences in cell morphology, CFU, or RNA yield in any of the strains or conditions examined in this study.

For RNA extraction, samples were extracted three times with 20 ml of 65°C phenol (Sigma P4682), 20 ml of Phenol∶Chloroform∶Isoamyl Alcohol (25∶24∶1) (Sigma P3803), and finally, 20 ml of Chloroform∶Isoamyl Alcohol (Sigma C0549), after which the final aqueous layer was removed to a fresh centrifuge tube and precipitated with 2.5 volumes of 100% ethanol (Sigma E7023). After washing the pellet with 25 ml 70% ethanol, the RNA was resuspended in 200 µl sterile RNase/DNase-free water and stored at −20°C. For small RNA, tRNA, and genomic DNA removal, the Qiagen RNEasy Mini-Kit RNA Cleanup protocol was used per the manufacturer's instructions for 100 µg of each raw RNA extraction with on-column DNase digestion. All samples were assayed for RNA integrity on the Bio-Rad Experion Automated Electrophoresis Station with Prokaryotic RNA StdSens chips.

After RNA cleanup, 10 µg of each RNA sample were used to make cDNA as follows: all RNA used had a 260/280 ratio≥1.7, and a 260/230 ratio≥1.5. (Following protocol based on Nimblegen cDNA synthesis procedures). The Invitrogen SuperScript II Double Stranded cDNA synthesis kit (Cat. no. 11917) was used per standard protocols (full detailed protocol available at http://bergmanlab.biology.gatech.edu/), with 200 ng Invitrogen Random hexamer primers for first-strand synthesis. All RNA and cDNA concentrations were checked on a Nanodrop 1000 spectrophotometer.

### Growth Curves

Strains *B. anthracis* Sterne 34F_2_, *B. cereus* 10987, and *B. cereus* G9241 were grown on TSA blood agar plates. Colonies were picked to inoculate 3 ml of LB medium and grown at 37°C to OD_600_∼0.4–0.5. These cultures were used to inoculate 30 ml of MGM or MGM+0.8% Sodium Bicarbonate to an OD_600_ of ∼0.01. Cultures were shaken at 250 rpm at 37°C for 6 hours (bicarbonate flasks incubated in 14–15% CO_2_ conditions) and OD_600_ was recorded every hour. Growth was plotted in Prism 5 for Mac OSX. Doubling times were calculated per the method of Moat, Foster & Spector [56] using OD_600_.

### Microarray Expression Analysis

cDNA prepared as stated above was sent to Roche Nimblegen to be run on Gene Expression Microarrays: *B. anthracis* Ames Ancestor chip design TI261594 (Catalog design for *Bacillus anthracis* str. ‘Ames Ancestor’ covering NC_007322, NC_007323, NC_007530. Probes selected for 5617/5617 sequences. Median number of probes/sequence is 17 with an average of 17.00.); *B. cereus* 10987 chip design TI222523 (Catalog design for *Bacillus cereus* ATCC 10987 covering NC_003909, NC_005707. Probes selected for 5844/5844 sequences. Median number of probes/sequence is 13 with an average of 13.00.); and a custom *B. cereus* G9241 chip designed by Nimblegen using files provided by Timothy Read, containing 6147 sequences, resulting in an array with 14 probes per gene and 5 copies of each probe. In total, 30 expression arrays were run as follows: for each of the 3 *Bacillus* species, 5 arrays were run for each condition (ambient air versus CO_2_ for 10 arrays/species), representing 4 biological replicates and one additional replicate representing cDNA made from a mix of RNA from each of 4 biological replicates. Raw expression data were normalized using RMA [57]. Fluorescence intensities for each of the probe replicates were averaged, log_2_-transformed, and used for further analysis. Expression analyses were done on Mev 4.0 (TIGR software) for Mac OSX. Significance Analysis of Microarrays (SAM) analyses were done with a false discovery rate (FDR)<0.001 and a fold-change cutoff of 2.0. Sample Density Matrices were also generated in Mev 4.0. Functional family analyses were done using the EASE algorithm [58] and TIGRFAM and GO tables compiled from the TIGR Comprehensive Microbial Resource (http://cmr.jcvi.org/tigr-scripts/CMR/CmrHomePage.cgi). Note that for *B. cereus* G9241, a functional family list was created to correspond to its closest hits in the *B. anthracis* genome as follows. BLAST was performed for all G9241 genes against a formatted database consisting of genes from the *B. anthracis* Ames Ancestor strain. The e-value cutoff was 1e-20. In cases of genes for which more than one homolog were found, only the best BLAST hit was considered and the others were ignored. The genes found in G9241 were mapped to the families of their corresponding homologs found in the *B. anthracis* Ames Ancestor list. The genes from G9241 that had no homologs in *B. anthracis* were classified as “unique” genes and were stored in a separate file.

### Microarray Data

Microarray data are available in the EBI ArrayExpress database under accession number E-MEXP-2036.

### SYBR-Green quantitative RT-PCR of select genes

SYBR Green quantitative RT-PCR (qRT-PCR) was used to confirm differential transcript expression between samples. For *B. anthracis* 34F_2_, 8 genes were chosen for analysis: 5 located on the chromosome and 3 located on the pXO1 plasmid. For *B. cereus* G9241, 12 genes were chosen for analysis: 5 located on the chromosome; 4 located on the pBC218 plasmid, and 3 putatively located on the pBCXO1 plasmid (see Supplemental Tables S11 and S12 for gene names and primer sequences). The *fusA* gene (*B. anthracis* GBAA 0107), which has been previously shown to be expressed constitutively in a wide variety of growth conditions [9], [55] and its closest homolog in *B. cereus* G9241 (BCE_G9241_0105) were used for relative quantitation of transcripts. Primers were designed to amplify sequences within the open reading frames and to result in PCR products between 150–200 base pairs. All primer pairs were tested with genomic DNA to confirm that only one amplification product was produced at the optimal annealing temperature. Experiments were performed using Applied Biosystems (ABI) Power RNA-to-Ct SYBR Mix 1-Step (ABI 4389986) with 3 experimental replicates, 2 no RT controls, and 1 no RNA control (all negative). 20 µl reactions were mixed in 96-well ABI Optical Reaction Plates as follows: 10 µl 2× Reaction Mix, 2 µl forward primer and 2 µl reverse primer (100 nM final), 0.16 µl RT mix, 1 µl RNA (70 ng total) and 4.84 µl H_2_O. Each primer pair was used for a separate assay using RNA from CO_2_ and from O_2_ samples. Plates were analyzed on an ABI Prism 7000 with the following protocol: 1× 48°C 30 min, 1× 95°C 10 min, 40× 95°C 15 s, 60°C 60 s. Data were analyzed using the ABI Prism 7000 SDS Software with the Auto Ct as threshold. Microarray fluorescence intensities (the average of 5 biological replicates - log_2_) and qRT-PCR Ct values (averages of 3 technical replicates) were plotted in Prism 5 for Mac OSX and analyzed by a Spearman Rank Correlation test.

### Motility Assays

Planktonic cells of strains *B. cereus* G9241, *B. cereus* 10987, *B. anthracis* Sterne 34F_2_, and *B. subtilis* 168 (obtained from ATCC) were mounted on glass cover slips and hanging drop slides and observed under 40× magnification via phase contrast microscopy for motility. Bacteria were grown as follows: fresh colonies from a TSA blood-agar plate were picked to inoculate 15 ml of LB medium, MGM, and MGM+0.8% sodium bicarbonate, and grown at 37°C with normal aeration or with 10% CO_2_ for bicarbonate cultures, with shaking at 200 rpm to mid log phase (OD_600_∼0.4–0.5). Flasks were sealed with “Bug-Stopper” vented plugs for gas exchange.

### Bioinformatic analyses for Venn Diagram

FASTA protein sequences from *Bacillus anthracis* Ames Ancestor (AA), *Bacillus cereus* G9241, and *Bacillus cereus* 10987 genomes were compared to each other using a local installation of BLAST as follows: AA versus G9241, G9241 versus 10987, and 10987 versus AA. Then, 2 lists were compiled as follows. List one comprised genes between AA and G9241 that shared >90% protein identity at the amino acid level (total = 3,076). List two comprised genes that shared >90% identity at the amino acid level across all three genomes (total = 2,624). If more than one gene shared identity at >90%, the higher value was kept. For Venn diagrams, all genes that were differentially expressed in each species per SAM analysis were compared to the aforementioned 90% lists, and those contained on the list(s) were separated into a new “conserved & differentially expressed” list. Then, these conserved expression lists were compared to each other for Venn diagram counts.

### Nucleotide sequence alignments for intergenic sequences

Nucleotide sequence alignments for the 150 base pair regions for *B. anthracis*, *B. cereus* G9241 and *B. cereus* 10987 directly upstream of the petrobactin and *dhb* biosynthetic operons were done on CLC DNA Workbench version 4.1.2 for Mac OSX, on the slow (very accurate) setting with Gap Open and Gap Extension Costs of 10 (default).

## Supporting Information

Table S1 Genes more highly expressed in CO_2_+bicarbonate for B. cereus G9241 and B. anthracis Sterne 34F2 that share >90% protein identity
(0.13 MB PDF)

Table S2 Genes more highly expressed in O_2_ for B. cereus G9241 and B. anthracis Sterne 34F2 that share >90% protein identity
(0.12 MB PDF)

Table S3 Hypothetical and Unknown Function Chromosomal genes with increased expression (≥6-fold) in CO_2_+0.8% bicarbonate
(0.11 MB PDF)

Table S4 Hypothetical and Unknown Function Chromosomal genes with increased expression (≥6-fold) in O_2_
(0.10 MB PDF)

Table S5 Genes with increased expression in B. cereus G9241 in CO_2_ (MGM+0.8% bicarbonate)
(0.27 MB PDF)

Table S6 Genes with increased expression in B. cereus G9241 in MGM in O_2_
(0.30 MB PDF)

Table S7 Genes with increased expression in B. anthracis Sterne (34F2) in CO_2_ (MGM+0.8% bicarbonate)
(0.17 MB PDF)

Table S8 Genes with increased expression in B. anthracis Sterne (34F2) in MGM in O_2_
(0.16 MB PDF)

Table S9 Genes with increased expression in B. cereus 10987 in CO_2_ (MGM+0.8% bicarbonate)
(0.12 MB PDF)

Table S10 Genes with increased expression in B. cereus 10987 in MGM in O_2_
(0.10 MB PDF)

Table S11 Primers for SYBR-Green qRT-PCR for B. anthracis Sterne 34F2
(0.08 MB PDF)

Table S12 Primers for SYBR-Green qRT-PCR for B. cereus G9241
(0.08 MB PDF)

Table S13 Putative motility genes more highly expressed in O_2_ for B. cereus G9241 and B. anthracis Sterne (34F2)
(0.08 MB PDF)

Table S14 Putative S-Layer genes differentially expressed in CO_2_ and O_2_ in three Bacilli.
(0.08 MB PDF)
