# Supplementary material for: Comparative Transcriptional Profiling of Bacillus cereus Sensu Lato Strains during Growth in CO2-Bicarbonate and Aerobic Atmospheres
Source: PLoS One. 2009 Mar 19;4(3):e4904. doi: 10.1371/journal.pone.0004904 (PMC2654142; doi:10.1371/journal.pone.0004904)
Supplement: Table S1 — Genes more highly expressed in CO2+bicarbonate for B. cereus G9241 and B. anthracis Sterne 34F2 that share >90% protein identity (0.13 MB PDF) [file pone.0004904.s001.pdf]

**Table S1. Genes more highly expressed in CO_2 + bicarbonate for *B. cereus* G9241 and *B. anthracis* Sterne 34F_2 that share > 90% protein identity**					
B. anthracis			***B. cereus* G9241**		
[a]**gene name**	[b]**locus tag #**	[c]**FD**	[a]**gene name**	[b]**locus tag #**	[c]**FD**
S-layer protein, (pxo1-90)	pXO1_0124	35.13	S-layer homology domain protein	pBCXO1_0105	12.59
phosphatase, pap2 family, (pxo1-91)	pXO1_0125	12.03	PAP2 superfamily domain protein	pBCXO1_0106	9.54
hypothetical protein	pXO1_0138	2.71	conserved hypothetical protein	pBCXO1_0116	25.80
hypothetical protein	pXO1_0139	2.67	conserved hypothetical protein	pBCXO1_0117	53.08
hypothetical protein	pXO1_0140	2.28	conserved hypothetical protein	pBCXO1_0118	40.10
calmodulin-sensitive adenylate cyclase	pXO1_0142	2.08	*cya*; calmodulin-sensitive adenylate cyclase	pBCXO1_0119	10.58
transcriptional activator atxa, (pxo1-119)	pXO1_0146	5.24	conserved hypothetical protein	pBCXO1_0126	5.61
cation-transporting atpase, e1-e2 family	0405	8.06	cation-transporting ATPase E1-E2 family	0415	8.04
penicillin-binding domain protein	0543	2.10	*mrcA*; penicillin-binding protein 1A	0499	4.03
hypothetical protein	0789	3.37	outer surface protein	0776	5.99
transcription antiterminator, bglg family	0790	2.29	transcription regulator probable	0777	2.08
abc transporter, permease protein, putative	0797	2.34	*ybjZ*; ABC transporter ATP-binding protein	0785	5.11
n-acetylmuramoyl-l-alanine amidase, family 2	0872	2.28	N-acetylmuramoyl-L-alanine amidase	0874	3.08
sulfate permease family protein	0878	2.62	sulfate transporter	0882	2.06
ornithine cyclodeaminase	0902	2.32	*arcB*; ornithine cyclodeaminase	0906	6.08
hypothetical protein	1005	6.70	conserved hypothetical protein	1030	3.70
hypothetical protein	1006	4.13	conserved hypothetical protein	1031	39.89
hypothetical protein	1007	4.06	conserved hypothetical protein	1032	15.37
acyl-CoA synthase	1091	7.24	*lfadD-7*; long-chain-fatty-acid--CoA ligase	1092	2.48
S-layer protein, putative	1130	6.54	S-layer homology domain	1115	2.52
hypothetical protein	1143	2.16	hypothetical protein cytosolic	1129	2.02
fabF; 3-oxoacyl-(acyl carrier protein) synthase	1185	2.03	beta-ketoacyl synthase N-terminal domain protein	1175	3.33
hypothetical protein	1354	6.18	conserved hypothetical protein	1351	15.25
hisF; imidazole glycerol phosphate synthase subunit HisF	1430	2.02	*hisF*; imidazoleglycerol phosphate synthase cyclase subunit	1426	2.72
hypothetical protein	1470	3.98	hypothetical protein membrane Spanning protein	1468	2.29
gpsA; NAD(P)H-dependent glycerol-3-phosphate dehydrogenase	1526	3.05	*gpsA*; NAD-dependent glycerol-3-phosphate dehydrogenase superfamily	1525	2.67
qcrC; menaquinol-cytochrome c reductase, cytochrome b/c subunit	1546	2.50	cytochrome b-c complex cytochrome b subunit putative	1544	4.62
panC; pantoate--beta-alanine ligase	1563	4.52	*panC*; pantoate--beta-alanine ligase	1561	2.18
germination protein gern	1639	11.70	Na+/H+ antiporter	1647	5.26
3-oxoacyl-(acyl carrier protein) synthase	1826	3.03	*fabH*; 3-oxoacyl-(acyl-carrier-protein) synthase III subfamily	1828	6.89
bnr repeat domain protein	1900	7.66	BNR/Asp-box repeat domain protein	1899	7.49
cydB-1; cytochrome d ubiquinol oxidase, subunit ii	1944	36.62	*cydB*; cytochrome d ubiquinol oxidase subunit II	1952	17.33

**Table S1. Genes more highly expressed in CO_2 + bicarbonate for *B. cereus* G9241 and *B. anthracis* Sterne $34F_2$ that share > 90% protein identity**					
B. anthracis			***B. cereus* G9241**		
[a]**gene name**	[b]**locus tag #**	[c]**FD**	[a]**gene name**	[b]**locus tag #**	[c]**FD**
transport atp-binding protein cydc	1945	65.50	ABC transporter ATP-binding protein CydD	1953	29.09
transport atp-binding protein cydd	1946	24.51	ABC transporter ATP-binding protein CydC	1954	45.53
dpS; general stress protein	2013	2.65	*dps*; non-specific DNA-binding protein Dps / ron- binding ferritin-like antioxidant protein / ferroxidase	2031	5.02
precorrin-2 dehydrogenase	2142	3.63	siroheme synthase putative	2150	2.03
cbix domain protein	2143	3.65	transcriptional regulator NirR putative	2151	2.84
sco1/senc family lipoprotein	2249	2.97	cytochrome c oxidase Cu(A) center assembly protein	2214	2.42
alcohol dehydrogenase	2267	7.62	*adhP*; alcohol dehydrogenase propanol-preferring	2229	4.33
kamA; l-lysine 2,3-aminomutase	2300	23.71	*kamA*; L-lysine 2,3-aminomutase	2268	26.24
hypothetical protein	2301	34.72	YokU	2269	11.83
sporulation-control protein spo0m, putative	2308	4.49	sporulation-control protein	2277	3.90
garR; 2-hydroxy-3-oxopropionate reductase	2353	2.15	3-hydroxyisobutyrate dehydrogenase	2318	2.45
mmsA-1; methylmalonic acid semialdehyde dehydrogenase	2354	2.21	*mmsA*; methylmalonate-semialdehyde dehydrogenase	2319	3.31
hypothetical protein	2839	9.82	conserved hypothetical protein	2788	3.67
hypothetical protein	2840	10.30	conserved hypothetical protein	2789	2.82
metallo-beta-lactamase family protein	3118	3.81	metal-dependent hydrolase	3060	2.02
hypothetical protein	3239	2.04	putative phosphohydrolases Icc family	3187	2.53
hypothetical protein	3359	2.28	SWIM zinc finger family	3249	2.26
hypothetical protein	3420	3.48	conserved hypothetical protein	3317	2.05
dhaS; aldehyde dehydrogenase	3609	4.08	*dhaS*; aldehyde dehydrogenase family protein	3500	4.28
anaerobic ribonucleoside triphosphate reductase	3663	11.83	anaerobic ribonucleoside-triphosphate reductase putative	3555	8.06
heavy metal-transporting atpase	3859	6.55	copper-translocating P-type ATPase	3695	4.61
copper-ion-binding protein	3860	10.70	*pacS*; cation-transporting ATPase P-type	3696	6.80
ftsY; signal recognition particle-docking protein ftsy	3895	2.23	phosphoglycerol transferase	3736	8.40
ctaE; cytochrome c oxidase, subunit iii	4152	2.41	cytochrome c oxidase subunit III	3931	3.72
ctaD; cytochrome c oxidase, subunit i	4153	2.27	*qoxA*; quinol oxidase subunit I	3932	3.77
ctaC; cytochrome c oxidase, subunit ii	4154	2.03	cytochrome c oxidase polypeptide II	3933	3.06
ctaA; cytochrome aa3 controlling protein	4156	2.34	*ctaA*; cytochrome oxidase assembly protein	3935	2.02
metE; 5-methyltetrahydropteroyltriglutamate--homocysteine methyltransferase	4218	2.37	*metE*; 5-methyltetrahydropteroyltriglutamate--homocysteine S-methyltransferase	3996	3.11
hypothetical protein	4224	17.83	conserved hypothetical protein	4002	9.65
universal stress protein family	4875	4.90	universal stress protein family	4709	5.84

**Table S1. Genes more highly expressed in CO_2 + bicarbonate for *B. cereus* G9241 and *B. anthracis* Sterne $34F_2$ that share > 90% protein identity**					
B. anthracis			***B. cereus* G9241**		
[a]**gene name**	[b]**locus tag #**	[c]**FD**	[a]**gene name**	[b]**locus tag #**	[c]**FD**
acetyl-coa synthetase, putative	4896	2.98	*acs-6*; acetyl-CoA synthetase	4729	4.67
sodium/hydrogen exchanger family protein	4993	2.72	Na+/H+ antiporter putative	4836	5.99
trka domain protein	4994	2.32	TrkA C-terminal domain protein	4837	3.23
hypothetical protein	5071	2.90	conserved hypothetical protein	4923	4.13
acyl-coa dehydrogenase	5246	2.44	*acd-7*; acyl-CoA dehydrogenase	5102	7.44
3-hydroxyacyl-coa dehydrogenase/enoyl-coa hydratase/isomerase family protein	5249	2.09	*hbd-1*; putative 3-hydroxyacyl-CoA dehydrogenase FadB	5105	12.11

[a]Gene names are listed for both species because they are slightly different as listed in the annotations.
[b]Locus tag numbers are from the *B. anthracis* Ames Ancestor (GBAAXXXX) and the *B. cereus* G9241 (BCE_G9241_XXXX) genomes.
[c]Fold Differences between two conditions as assessed by SAM (see Methods).
