# Supplementary material for: Comparative Transcriptional Profiling of Bacillus cereus Sensu Lato Strains during Growth in CO2-Bicarbonate and Aerobic Atmospheres
Source: PLoS One. 2009 Mar 19;4(3):e4904. doi: 10.1371/journal.pone.0004904 (PMC2654142; doi:10.1371/journal.pone.0004904)
Supplement: Table S2 — Genes more highly expressed in O2 for B. cereus G9241 and B. anthracis Sterne 34F2 that share >90% protein identity (0.12 MB PDF) [file pone.0004904.s002.pdf]

**Table S2. Genes more highly expressed in O_2 for *B. cereus* G9241 and *B. anthracis* Sterne $34F_2$ that share > 90% protein identity**					
B. anthracis			***B. cereus* G9241**		
**[a]gene name**	**[b]locus tag #**	**[c]FD**	**[a]gene name**	**[b]locus tag #**	**[c]FD**
acpS; holo-(acyl-carrier-protein) synthase	0250	2.43	*acpS*; holo-(acyl-carrier-protein) synthase	0240	4.21
lipoprotein, putative	0251	2.56	conserved hypothetical protein	0241	2.56
abc transporter, atp-binding protein	0532	2.27	daunorubicin resistance ATP-binding protein drrA	0485	6.30
abc transporter, permease protein, putative	0533	2.20	membrane protein putative	0486	4.83
abc transporter, permease protein, putative	0534	2.05	ABC transporter permease protein	0487	3.24
potassium channel protein, putative	0535	2.14	LCTB protein	0488	4.30
methyl-accepting chemotaxis protein	0558	2.44	*mcpB*; methyl-accepting chemotaxis protein	0538	2.53
acetyltransferase, gnat family	0587	8.85	acetyltransferase GNAT family putative	0563	3.54
treB; pts system, trehalose-specific iibc component	0631	20.61	*treP*; PTS system trehalose-specific IIBC component	0608	84.83
treC; trehalose-6-phosphate hydrolase	0632	26.81	trehalose-6-phosphate hydrolase	0609	47.16
glpT; glycerol-3-phosphate transporter	0661	16.60	*glpT*; glycerol-3-phosphate transporter	0644	26.69
transcriptional regulator, marr family	0662	4.02	transcriptional regulator MarR family	0645	8.47
rbsR; ribose operon repressor	0664	2.05	ribose operon repressor RbsR	0647	4.23
rbsK; ribokinase	0665	2.46	*rbsK*; ribokinase	0648	3.97
methyl-accepting chemotaxis protein	0684	2.44	methyl-accepting chemotaxis protein putative	0665	4.43
S-layer protein, putative	0981	2.78	S-layer homology domain	0996	28.32
glpD; glycerol-3-phosphate dehydrogenase, aerobic	1027	64.69	*glpD*; aerobic glycerol-3-phosphate dehydrogenase	1045	9.80
transcriptional regulator, gntr family	1314	2.49	transcriptional regulator GntR family putative	1313	2.97
abc transporter, atp-binding protein	1374	2.02	ABC transporter ATP-binding protein (yhcG)	1370	2.36
cspB-1; cold shock protein cspb	1629	2.22	cold shock protein-related protein	1637	3.32
flagellar motor switch protein	1662	2.14	chemotaxis protein cheC	1672	4.21
proton/sodium-glutamate symporter	1799	13.04	*gltT*; proton/sodium-glutamate symport protein	1803	46.21
ykwA; malate oxidoreductase	1801	5.46	*malS*; malate oxidoreductase VC1188	1806	15.88
sensor histidine kinase	1802	4.17	sensor histidine kinase putative	1807	16.00
response regulator	1803	3.91	response regulator putative	1808	10.42
acetyl-coa hydrolase/transferase family protein	1862	4.36	*abfT-1*; acetyl-CoA hydrolase/transferase family protein	1861	2.22
hypothetical protein	2011	2.55	conserved hypothetical protein	2029	2.46
hypothetical protein	2118	4.39	conserved hypothetical protein	2128	6.73
oxalate:formate antiporter, putative	2367	77.66	oxalate/formate antiporter putative	2332	19.88
5'-methylthioadenosine/S-adenosylhomocysteine nucleosidase	2564	2.92	*mtn*; S-adenosylhomocysteine nucleosidase	2518	2.92
acetyltransferase, gnat family	2744	3.21	MW2053	2693	2.56

Table S2. Genes more highly expressed in O_2 for *B. cereus* G9241 and *B. anthracis* Sterne $34F_2$ that share > 90% protein identity					
B. anthracis			***B. cereus* G9241**		
**[a]gene name**	**[b]locus tag #**	**[c]FD**	**[a]gene name**	**[b]locus tag #**	**[c]FD**
hisC-2; putative aminotransferase	2955	2.71	*hisC*; histidinol-phosphate aminotransferase	2916	7.11
aroF-2; chorismate synthase	2956	2.51	*aroC*; chorismate synthase	2917	5.46
3-deoxy-7-phosphoheptulonate synthase	2958	2.16	MW1680	2918	3.68
proB; gamma-glutamyl kinase	2993	3.29	*proB*; glutamate 5-kinase	2947	2.47
hypothetical protein	3077	2.07	HesB-like protein	3020	2.68
aspA-3; aspartate ammonia-lyase	3136	6.85	*aspA*; aspartate ammonia-lyase	3066	13.22
ansA-2; l-asparaginase	3137	3.04	L-asparaginase I putative	3067	2.67
amino acid permease family protein	3141	5.95	amino acid permease	3082	2.01
proC; pyrroline-5-carboxylate reductase	3143	14.23	*proC*; pyrroline-5-carboxylate reductase	3083	2.22
glsA-2; glutaminase	3155	16.03	glutaminase A	3090	3.62
5'-nucleotidase, putative	3162	3.56	*ushA*; 5'-nucleotidase	3096	2.41
hypothetical protein	3482	2.57	hypothetical protein membrane Associated	3368	2.63
glycerophosphoryl diester phosphodiesterase, putative	3560	40.91	glycerophosphoryl diester phosphodiesterase	3442	6.15
hypothetical protein	3629	2.03	hypothetical protein cytosolic	3519	3.85
als operon regulatory protein alsr, putative	3729	2.28	transcriptional regulator LysR family	3631	2.75
hypothetical protein	4321	2.01	conserved hypothetical protein	4104	2.29
cpdB; 2',3'-cyclic nucleotide 2'-phosphodiesterase/3'-nucleotidase bifunctional periplasmic precursor protein	4346	3.39	*cpdB*; 2'3'-cyclic-nucleotide 2'-phosphodiesterase	4131	4.75
drug resistance transporter, emrb/qaca family	4961	3.99	drug resistance transporter EmrB/QacA family protein	4804	5.89
abc transporter, atp-binding protein	5217	2.23	*sufC*; FeS assembly ATPase sufC	5079	3.77
conserved hypothetical protein tigr00106	5255	2.32	conserved hypothetical protein TIGR00106	5112	2.04
methyl-accepting chemotaxis protein	5256	5.15	methyl-accepting chemotaxis transducer putative	5113	5.64
amino acid permease family protein	5261	5.25	amino acid permease	5119	2.19
methyl-accepting chemotaxis protein	5317	2.85	methyl-accepting chemotaxis protein putative	5185	3.43
endonuclease/exonuclease/phosphatase family	5318	3.46	endonuclease/exonuclease/phosphatase family protein putative	5186	6.57
abc transporter, permease protein	5649	2.25	bacitracin transport permease protein BCRB	5583	2.44
abc transporter, atp-binding protein	5650	2.94	bacitracin transport ATP-binding protein bcrA	5584	2.63
lipase/acylhydrolase, putative	5651	2.59	lipase/acylhydrolase putative	5585	5.14
response regulator, putative	5667	2.50	response regulator putative	5603	6.74
ATP-dependent RNA helicase, dead/deah box family	5703	2.33	*deaD*; ATP-dependent RNA helicase DEAD/DEAH box family	5643	2.85

[a]Gene names are listed for both species because they are slightly different as listed in the annotations.
[b]Locus tag numbers are from the *B. anthracis* Ames Ancestor (GBAAXXXX) and the *B. cereus* G9241 (BCE_G9241_XXXX) genomes.
[c]Fold Differences between two conditions as assessed by SAM (see Methods).
