# Supplementary material for: Comparative Transcriptional Profiling of Bacillus cereus Sensu Lato Strains during Growth in CO2-Bicarbonate and Aerobic Atmospheres
Source: PLoS One. 2009 Mar 19;4(3):e4904. doi: 10.1371/journal.pone.0004904 (PMC2654142; doi:10.1371/journal.pone.0004904)
Supplement: Table S3 — Hypothetical and Unknown Function Chromosomal genes with increased expression (≥6-fold) in CO2+0.8% bicarbonate (0.11 MB PDF) [file pone.0004904.s003.pdf]

**Table S3: Hypothetical and Unknown Function Chromosomal genes with increased expression (≥ 6-fold) in CO_2 + 0.8% bicarbonate**		
**Gene name**	**locus #***	**Fold Difference (SAM)**
**UNKNOWN FUNCTION**		
***B. cereus* G9241**		
outer surface protein	BCE_G9241_0782	**12.24**
maoC; enoyl-CoA hydratase R-specific	BCE_G9241_1324	**14.54**
PhaP protein	BCE_G9241_1325	**11.38**
transcriptional regulator PadR family, putative	BCE_G9241_1327	**8.68**
phage shock protein A, putative	BCE_G9241_1452	**9.83**
BNR/Asp-box repeat domain protein	BCE_G9241_1899	**7.49**
YokU	BCE_G9241_2269	**11.83**
omt; caffeoyl-CoA O-methyltransferase	BCE_G9241_2420	**6.84**
putative esterase family	BCE_G9241_2657	**7.09**
chitin-binding protein, putative	BCE_G9241_2741	**18.66**
phnA; alkylphosphonate utilization operon protein PhnA	BCE_G9241_2986	**6.06**
CsbD-like family	BCE_G9241_3589	**12.74**
phosphoglycerol transferase	BCE_G9241_3736	**8.40**
***B. anthracis* Sterne $34F_2$**		
bnr repeat domain protein	GBAA1900	**7.66**
metallo-beta-lactamase/rhodanese-like domain protein	GBAA2538	**8.77**
carboxyl transferase domain protein	GBAA2552	**6.68**
bnr repeat domain protein	GBAA1900	**7.66**
***B. cereus* 10987**		
PAP2 family protein	BCE5133	**6.25**
**HYPOTHETICAL PROTEINS**		
***B. cereus* G9241**		
hypothetical protein	BCE_G9241_0189	**10.41**
hypothetical protein, cytosolic protein	BCE_G9241_0635	**6.67**
conserved hypothetical protein	BCE_G9241_0781	**7.70**
hypothetical protein	BCE_G9241_0907	**9.57**
conserved hypothetical protein	BCE_G9241_1031	**39.89**
conserved hypothetical protein	BCE_G9241_1032	**15.37**
hypothetical protein	BCE_G9241_1326	**9.77**
conserved hypothetical protein	BCE_G9241_1351	**15.25**
conserved hypothetical protein	BCE_G9241_1391	**6.87**
conserved hypothetical protein	BCE_G9241_1451	**6.40**
hypothetical protein	BCE_G9241_1593	**8.14**
hypothetical protein	BCE_G9241_2740	**17.84**
hypothetical protein	BCE_G9241_2857	**6.04**
hypothetical protein	BCE_G9241_2871	**16.98**
conserved hypothetical protein	BCE_G9241_4002	**9.65**
conserved hypothetical protein	BCE_G9241_4043	**6.54**
conserved hypothetical protein	BCE_G9241_4250	**9.61**
hypothetical protein	BCE_G9241_4531	**6.45**
conserved hypothetical protein	BCE_G9241_4532	**7.75**
conserved hypothetical protein	BCE_G9241_4913	**6.49**
hypothetical protein	BCE_G9241_5103	**9.48**
hypothetical protein	BCE_G9241_5106	**7.34**

**Table S3: Hypothetical and Unknown Function Chromosomal genes with increased expression (≥ 6-fold) in CO_2 + 0.8% bicarbonate**		
**Gene name**	**locus #***	**Fold Difference (SAM)**
***B. anthracis* Sterne 34F_2**		
hypothetical protein	GBAA0167	**10.17**
hypothetical protein	GBAA1005	**6.70**
hypothetical protein	GBAA1354	**6.18**
hypothetical protein	GBAA1947	**7.14**
hypothetical protein	GBAA2301	**34.72**
hypothetical protein	GBAA2535	**7.23**
hypothetical protein	GBAA2537	**7.96**
hypothetical protein	GBAA2693	**7.60**
hypothetical protein	GBAA2695	**9.14**
hypothetical protein	GBAA2839	**9.82**
hypothetical protein	GBAA2840	**10.30**
hypothetical protein	GBAA4224	**17.83**
hypothetical protein	GBAA4799	**6.56**
hypothetical protein	GBAA4800	**9.44**
***B. cereus* 10987**		
hypothetical protein	BCE2869	7.76
hypothetical protein	BCE2870	6.83

*Locus tag numbers from *B. cereus* G9241 (BCE_G9241), *B. anthracis* Ames Ancestor (GBAA) and *B. cereus* 10987 (BCE) genomes.
