# Supplementary material for: Comparative Transcriptional Profiling of Bacillus cereus Sensu Lato Strains during Growth in CO2-Bicarbonate and Aerobic Atmospheres
Source: PLoS One. 2009 Mar 19;4(3):e4904. doi: 10.1371/journal.pone.0004904 (PMC2654142; doi:10.1371/journal.pone.0004904)
Supplement: Table S4 — Hypothetical and Unknown Function Chromosomal genes with increased expression (≥6-fold) in O2 (0.10 MB PDF) [file pone.0004904.s004.pdf]

**Table S4: Hypothetical and Unknown Function Chromosomal genes with increased expression (≥ 6-fold) in O_2**		
**Gene name**	**locus #***	**Fold Difference (SAM)**
**UNKNOWN FUNCTION**		
***B. cereus* G9241**		
limonene cyclase	BCE_G9241_0911	**6.31**
Sb42 putative	BCE_G9241_0912	**9.07**
putative peptidoglycan binding domain protein	BCE_G9241_0920	**9.91**
putative peptidoglycan binding domain protein	BCE_G9241_0931	**18.56**
leukotoxin putative	BCE_G9241_1095	**29.98**
cysteine-rich domain family	BCE_G9241_1314	**9.14**
prolyl 4-hydroxylase alpha subunit	BCE_G9241_4265	**7.24**
protein erfK/srfK precursor	BCE_G9241_5135	**7.90**
PapR-related protein	BCE_G9241_5524	**13.97**
LrgB family protein	BCE_G9241_5626	**19.80**
***B. cereus* 10987**		
antiholin-like protein LrgB	BCE5571	**44.70**
**HYPOTHETICAL PROTEINS**		
***B. cereus* G9241**		
hypothetical protein	BCE_G9241_0726	**6.64**
hypothetical protein	BCE_G9241_0910	**10.95**
hypothetical protein	BCE_G9241_0919	**37.65**
hypothetical protein membrane Spanning protein	BCE_G9241_1010	**9.27**
conserved hypothetical protein	BCE_G9241_1678	**8.35**
conserved hypothetical protein	BCE_G9241_1792	**6.01**
conserved hypothetical protein	BCE_G9241_2128	**6.73**
conserved hypothetical protein	BCE_G9241_2993	**7.31**
hypothetical protein	BCE_G9241_3468	**43.50**
hypothetical protein	BCE_G9241_3784	**69.98**
hypothetical protein	BCE_G9241_3785	**6.98**
conserved hypothetical protein	BCE_G9241_4703	**7.20**
conserved hypothetical protein	BCE_G9241_4872	**9.80**
hypothetical protein	BCE_G9241_5053	**40.85**
conserved hypothetical protein	BCE_G9241_5090	**20.37**
conserved_hypothetical_protein_protein	BCE_G9241_5134	**35.82**
conserved hypothetical protein	BCE_G9241_5532	**8.25**
hypothetical protein	BCE_G9241_5627	**26.63**
hypothetical protein	BCE_G9241_CNI_0290	**69.42**
***B. anthracis* Sterne 34F_2**		
hypothetical protein	GBAA3144	**18.75**
hypothetical protein	GBAA3146	**6.87**
hypothetical protein	GBAA3147	**17.49**
hypothetical protein	GBAA3648	**8.66**
hypothetical protein	GBAA3845	**10.12**
hypothetical protein	GBAA5262	**8.52**

*Locus tag numbers from *B. cereus* G9241 (BCE_G9241), *B. anthracis* Ames Ancestor (GBAA) and *B. cereus* 10987 (BCE) genomes.
