# Supplementary material for: Comparative Transcriptional Profiling of Bacillus cereus Sensu Lato Strains during Growth in CO2-Bicarbonate and Aerobic Atmospheres
Source: PLoS One. 2009 Mar 19;4(3):e4904. doi: 10.1371/journal.pone.0004904 (PMC2654142; doi:10.1371/journal.pone.0004904)
Supplement: Table S5 — Genes with increased expression in B. cereus G9241 in CO2 (MGM+0.8% bicarbonate) (0.27 MB PDF) [file pone.0004904.s005.pdf]

Table S5. Genes with increased expression in *B. cereus* G9241 in CO_2 (MGM + 0.8% bicarbonate)		
**SEQUENCE ID**	**GENE INFO**	**Fold difference**
BCE_G9241_0136	*gerD*; spore_germination_protein_GerD	**3.83**
BCE_G9241_0146	*glmS*; glucosamine--fructose-6-phosphate_aminotransferase_isomerizing	**2.40**
BCE_G9241_0189	hypothetical_protein_protein	**10.41**
BCE_G9241_0195	hypothetical_protein_protein	**5.58**
BCE_G9241_0198	conserved_hypothetical_protein_protein_subfamily	**2.05**
BCE_G9241_0231	*hpcE-1*; 2-hydroxyhepta-24-diene-17-dioate_isomerase	**2.07**
BCE_G9241_0232	*hmgA*; homogentisate_12-dioxygenase	**2.59**
BCE_G9241_0347	ferrichrome_binding_protein	**2.47**
BCE_G9241_0348	ferrichrome_transport_permease_fhuB	**4.64**
BCE_G9241_0368	fatty_acid_desaturase	**3.91**
BCE_G9241_0370	amino_acid_ABC_transporter_periplasmic_amino_acid-binding_protein	**2.14**
BCE_G9241_0372	*glnQ*; glutamine_ABC_transporter_ATP-binding_protein	**2.14**
BCE_G9241_0407	surface_protein	**2.05**
BCE_G9241_0415	cation-transporting_ATPase_E1-E2_family	**8.04**
BCE_G9241_0466	dihydroorotase	**3.39**
BCE_G9241_0467	conserved_hypothetical_protein_protein_TIGR01777	**3.06**
BCE_G9241_0499	*mrcA*; penicillin-binding_protein_1A	**4.03**
BCE_G9241_0594	ubiquinone/menaquinone_biosynthesis_methyltransferase_UBIE	**3.23**
BCE_G9241_0635	hypothetical_protein_cytosolic_protein	**6.67**
BCE_G9241_0703	acriflavin_resistance_plasma_membrane_protein	**2.68**
BCE_G9241_0755	nitroreductase_family_protein_putative	**4.20**
BCE_G9241_0776	outer_surface_protein	**5.99**
BCE_G9241_0777	transcription_regulator_probable_putative	**2.08**
BCE_G9241_0778	PTS_system_IIA_component	**6.19**
BCE_G9241_0779	PTS_system_IIB_component	**5.97**
BCE_G9241_0780	*celB*; PTS_system_IIC_component	**5.80**
BCE_G9241_0781	conserved_hypothetical_protein_protein	**7.70**
BCE_G9241_0782	outer_surface_protein	**12.24**
BCE_G9241_0785	*ybjZ*; ABC_transporter_ATP-binding_protein	**5.11**
BCE_G9241_0786	ABC_transporter_ATP-binding_protein	**5.32**
BCE_G9241_0787	periplasmic_component_of_efflux_system	**4.33**
BCE_G9241_0788	hypothetical_protein_protein	**3.26**
BCE_G9241_0791	peptide_ABC_transporter_permease_protein_putative	**2.03**
BCE_G9241_0863	putative_transcriptional_regulator	**2.04**
BCE_G9241_0874	N-acetylmuramoyl-L-alanine_amidase	**3.08**
BCE_G9241_0882	sulfate_transporter	**2.06**
BCE_G9241_0883	hypothetical_protein_protein	**2.06**
BCE_G9241_0905	proline_racemase_putative	**2.45**
BCE_G9241_0906	*arcB*; ornithine_cyclodeaminase	**6.08**
BCE_G9241_0907	hypothetical_protein_protein	**9.57**
BCE_G9241_1030	conserved_hypothetical_protein_protein	**3.70**
BCE_G9241_1031	conserved_hypothetical_protein_protein	**39.89**

Table S5. Genes with increased expression in *B. cereus* G9241 in CO_2 (MGM + 0.8% bicarbonate)		
**SEQUENCE ID**	**GENE INFO**	**Fold difference**
BCE_G9241_1032	conserved_hypothetical_protein_protein	**15.37**
BCE_G9241_1037	glyoxalase_family_protein	**2.35**
BCE_G9241_1038	glyoxylase_family_protein	**2.25**
BCE_G9241_1052	*rep*; ATP-dependent_DNA_helicase_rep	**2.44**
BCE_G9241_1053	ATP-dependent_DNA_helicase_rep	**3.02**
BCE_G9241_1092	*lfadD-7*; ong-chain-fatty-acid--CoA_ligase	**2.48**
BCE_G9241_1097	dienelactone_hydrolase_domain_protein	**2.05**
BCE_G9241_1102	transcriptional_regulator_AraC_family_putative	**4.13**
BCE_G9241_1115	S-layer_homology_domain	**2.52**
BCE_G9241_1129	hypothetical_protein_cytosolic_protein	**2.02**
BCE_G9241_1174	hypothetical_protein_protein	**2.13**
BCE_G9241_1175	beta-ketoacyl_synthase_N-terminal_domain_protein	**3.33**
BCE_G9241_1189	hypothetical_protein_protein	**2.54**
BCE_G9241_1205	Hep_Hag_family	**2.27**
BCE_G9241_1228	ATP-dependent_DNA_helicase	**2.03**
BCE_G9241_1259	NADH_oxidase	**2.01**
BCE_G9241_1260	hypothetical_protein_cytosolic_protein	**2.28**
BCE_G9241_1261	conserved_hypothetical_protein_protein	**3.49**
BCE_G9241_1324	*maoC*; enoyl-CoA_hydratase_R-specific	**14.54**
BCE_G9241_1325	PhaP_protein	**11.38**
BCE_G9241_1326	hypothetical_protein_protein	**9.77**
BCE_G9241_1327	transcriptional_regulator_PadR_family_putative	**8.68**
BCE_G9241_1328	PhaR_protein	**4.73**
BCE_G9241_1329	*fabG*; 3-oxoacyl-(acyl-carrier-protein)_reductase	**4.55**
BCE_G9241_1330	polyhydroxyalkanoic_acid_synthase_VCA0688	**5.98**
BCE_G9241_1350	oligoendopeptidase_F_putative	**2.24**
BCE_G9241_1351	conserved_hypothetical_protein_protein	**15.25**
BCE_G9241_1353	sporulation_kinase	**2.44**
BCE_G9241_1380	muramoyltetrapeptide_carboxypeptidase_putative	**4.34**
BCE_G9241_1390	flavodoxin	**5.86**
BCE_G9241_1391	conserved_hypothetical_protein_protein	**6.87**
BCE_G9241_1422	imidazoleglycerol-phosphate_dehydratase	**2.58**
BCE_G9241_1423	*hisH*; imidazole_glycerol_phosphate_synthase_glutamine_amidotransferase_subunit	**2.73**
BCE_G9241_1424	phosphoribosylformimino-5-aminoimidazole_carboxamide_ribotide_isomerase	**2.54**
BCE_G9241_1425	conserved_hypothetical_protein_protein	**2.74**
BCE_G9241_1426	*hisF*; imidazoleglycerol_phosphate_synthase_cyclase_subunit	**2.72**
BCE_G9241_1427	phosphoribosyl-AMP_cyclohydrolase	**2.12**
BCE_G9241_1428	phosphoribosyl-ATP_pyrophosphohydrolase	**2.05**
BCE_G9241_1431	MW2224	**2.64**
BCE_G9241_1451	conserved_hypothetical_protein_protein	**6.40**
BCE_G9241_1452	phage_shock_protein_A_putative	**9.83**
BCE_G9241_1453	transporter_yvqF	**2.19**

Table S5. Genes with increased expression in *B. cereus* G9241 in CO_2 (MGM + 0.8% bicarbonate)		
**SEQUENCE ID**	**GENE INFO**	**Fold difference**
BCE_G9241_1454	sensor_histidine_kinase_VC1276	**2.56**
BCE_G9241_1455	*vraR*; DNA-binding_response_regulator_LuxR_family	**2.52**
BCE_G9241_1468	hypothetical_protein_membrane_Spanning_protein	**2.29**
BCE_G9241_1481	*deoD*; purine_nucleoside_phosphorylase	**2.23**
BCE_G9241_1483	*npr*; predicted_transposase	**2.03**
BCE_G9241_1484	hypothetical_protein_protein	**2.84**
BCE_G9241_1485	conserved_hypothetical_protein_protein	**2.10**
BCE_G9241_1497	cytochrome_c-type_biogenesis_protein_putative	**2.32**
BCE_G9241_1501	cell_wall_endopeptidase_family_M23/M37	**3.15**
BCE_G9241_1525	*gpsA*; NAD-dependent_glycerol-3-phosphate_dehydrogenase_superfamily	**2.67**
BCE_G9241_1542	cytochrome_b6-f_complex_iron-sulfur_subunit_putative	**3.01**
BCE_G9241_1543	menaquinol-cytochrome_c_reductase_cytochrome_b_subunit	**3.42**
BCE_G9241_1544	cytochrome_b-c_complex_cytochrome_b_subunit_putative	**4.62**
BCE_G9241_1561	*panC*; pantoate--beta-alanine_ligase	**2.18**
BCE_G9241_1579	spore_coat_protein	**2.29**
BCE_G9241_1593	hypothetical_protein_protein	**8.14**
BCE_G9241_1605	conserved_hypothetical_protein_protein	**3.47**
BCE_G9241_1617	hypothetical_protein_protein	**2.12**
BCE_G9241_1618	hypothetical_protein_protein	**2.51**
BCE_G9241_1619	hypothetical_protein_protein	**2.75**
BCE_G9241_1647	Na+/H+_antiporter	**5.26**
BCE_G9241_1650	conserved_hypothetical_protein_integral_membrane_protein_putative	**2.04**
BCE_G9241_1654	hypothetical_protein_membrane_Spanning_protein	**4.72**
BCE_G9241_1721	VanZ_like_family_family	**2.03**
BCE_G9241_1722	conserved_hypothetical_protein_protein	**3.99**
BCE_G9241_1723	conserved_hypothetical_protein_protein	**2.92**
BCE_G9241_1724	dienelactone_hydrolase_family	**3.91**
BCE_G9241_1725	JEMB	**3.83**
BCE_G9241_1726	transposase_putative	**3.69**
BCE_G9241_1727	reticulocyte_binding_protein	**5.23**
BCE_G9241_1728	collagen_adhesion_protein	**3.20**
BCE_G9241_1751	conserved_hypothetical_protein_protein	**2.45**
BCE_G9241_1772	*fumC*; fumarate_hydratase_class_II	**4.28**
BCE_G9241_1774	*fumC*; fumarate_hydratase_class_II	**4.48**
BCE_G9241_1818	conserved_hypothetical_protein_protein	**2.18**
BCE_G9241_1828	*fabH*; 3-oxoacyl-(acyl-carrier-protein)_synthase_III_subfamily	**6.89**
BCE_G9241_1830	*hflX*; GTP-binding_protein_hflX	**3.14**
BCE_G9241_1899	BNR/Asp-box_repeat_domain_protein	**7.49**
BCE_G9241_1951	*appC*; cytochrome_d_ubiquinol_oxidase_subunit_I	**13.42**
BCE_G9241_1952	*cydB*; cytochrome_d_ubiquinol_oxidase_subunit_II	**17.33**
BCE_G9241_1953	ABC_transporter_ATP-binding_protein_CydD	**29.09**
BCE_G9241_1954	ABC_transporter_ATP-binding_protein_CydC	**45.53**
BCE_G9241_1963	nitroreductase_family_protein	**3.23**

Table S5. Genes with increased expression in *B. cereus* G9241 in CO_2 (MGM + 0.8% bicarbonate)		
**SEQUENCE ID**	**GENE INFO**	**Fold difference**
BCE_G9241_1989	transcriptional_regulator_putative	**3.24**
BCE_G9241_1997	MW0093_putative	**3.70**
BCE_G9241_1998	siderophore_biosynthesis_protein_IucC_family	**4.25**
BCE_G9241_2001	conserved_hypothetical_protein_protein	**4.34**
BCE_G9241_2002	hypothetical_protein_cytosolic_protein	**5.62**
BCE_G9241_2016	ABC_transporter_permease_protein_putative	**5.64**
BCE_G9241_2023	conserved_hypothetical_protein_protein	**2.76**
BCE_G9241_2024	conserved_hypothetical_protein_protein	**3.58**
BCE_G9241_2031	*dps*; non-specific_DNA-binding_protein_Dps_/_iron-_binding_ferritin-like_antioxidant_protein_/_ferroxidase	**5.02**
BCE_G9241_2032	conserved_hypothetical_protein_protein	**2.79**
BCE_G9241_2036	flavin_monoamine_oxidase-related_protein	**2.34**
BCE_G9241_2060	acyl-CoA_hydrolase	**2.04**
BCE_G9241_2069	CBS_domain_protein	**3.31**
BCE_G9241_2079	conserved_hypothetical_protein_protein	**4.93**
BCE_G9241_2080	stomatin-like_protein	**5.79**
BCE_G9241_2081	hypothetical_protein_protein	**5.65**
BCE_G9241_2131	hypothetical_protein_conserved_protein_putative	**2.60**
BCE_G9241_2150	siroheme_synthase_putative	**2.03**
BCE_G9241_2151	transcriptional_regulator_NirR_putative	**2.84**
BCE_G9241_2176	bacillolysin	**2.41**
BCE_G9241_2200	hypothetical_protein_protein	**3.96**
BCE_G9241_2201	penicillin-binding_protein	**28.62**
BCE_G9241_2214	cytochrome_c_oxidase_Cu(A)_center_assembly_protein	**2.42**
BCE_G9241_2229	*adhP*; alcohol_dehydrogenase_propanol-preferring	**4.33**
BCE_G9241_2230	oligopeptide_transport_system_permease_protein_oppC	**3.63**
BCE_G9241_2262	*ctfA*; butyrate-acetoacetate_CoA-transferase_subunit_A	**2.31**
BCE_G9241_2263	coenzyme_A_transferase_beta_subunit	**2.68**
BCE_G9241_2264	peptidase_M20/M25/M40_family_putative	**2.96**
BCE_G9241_2265	beta-lysine_acetyltransferase	**2.24**
BCE_G9241_2268	*kamA*; L-lysine_23-aminomutase	**26.24**
BCE_G9241_2269	YokU	**11.83**
BCE_G9241_2277	sporulation-control_protein	**3.90**
BCE_G9241_2283	conserved_hypothetical_protein_protein	**3.18**
BCE_G9241_2309	penicillin-binding_protein_1A	**2.35**
BCE_G9241_2314	*mmgE*; 2-methylcitrate_dehydratase	**3.06**
BCE_G9241_2315	*prpB*; methylisocitrate_lyase	**2.65**
BCE_G9241_2317	*acdA*; acyl-CoA_dehydrogenase_short-chain_specific	**2.59**
BCE_G9241_2318	3-hydroxyisobutyrate_dehydrogenase	**2.45**
BCE_G9241_2319	*mmsA*; methylmalonate-semialdehyde_dehydrogenase	**3.31**
BCE_G9241_2322	enoly-CoA_hydratase/isomerase_family_protein	**3.30**
BCE_G9241_2334	*dhbC*; isochorismate_synthase	**3.48**
BCE_G9241_2335	yersiniabactin_synthetase_salycilate_ligase_component	**11.63**
BCE_G9241_2336	*dhbB*; isochorismatase	**5.31**

Table S5. Genes with increased expression in *B. cereus* G9241 in CO_2 (MGM + 0.8% bicarbonate)		
**SEQUENCE ID**	**GENE INFO**	**Fold difference**
BCE_G9241_2337	reticulocyte_binding_protein	**6.86**
BCE_G9241_2339	drug_transporter_putative	**8.91**
BCE_G9241_2340	EntD/Gsp/HetI/Sfp_family_protein_putative	**3.26**
BCE_G9241_2344	DinB_protein	**2.16**
BCE_G9241_2345	alkaline_protease_A	**3.96**
BCE_G9241_2353	ABC_transporter_ATP-binding_protein	**2.86**
BCE_G9241_2354	ABC_transporter_permease_protein_probable_SP0913_putative	**3.58**
BCE_G9241_2387	cold_shock_protein-related_protein	**2.05**
BCE_G9241_2420	*omt*; caffeoyl-CoA_O-methyltransferase	**6.84**
BCE_G9241_2421	tellurite_resistance_protein	**3.59**
BCE_G9241_2422	conserved_hypothetical_protein_protein	**3.13**
BCE_G9241_2460	SpoIISA_like_protein	**3.02**
BCE_G9241_2461	SpoIISA_like_protein	**2.56**
BCE_G9241_2464	*pepD-2*; aminoacyl-histidine_dipeptidase	**2.51**
BCE_G9241_2465	hypothetical_protein_protein	**2.58**
BCE_G9241_2468	Patatin-like_phospholipase_family	**2.00**
BCE_G9241_2503	*crt*; 3-hydroxybutyryl-CoA_dehydratase	**2.64**
BCE_G9241_2504	*pccB*; propionyl-CoA_carboxylase_beta_chain	**4.87**
BCE_G9241_2505	*acs-4*; acetyl-CoA_synthetase	**3.82**
BCE_G9241_2535	oligoendopeptidase_putative	**3.13**
BCE_G9241_2550	formamidase	**2.87**
BCE_G9241_2555	ABC_transporter_ATP-binding_protein_putative	**4.92**
BCE_G9241_2556	acetyltransferase	**7.27**
BCE_G9241_2575	conserved_hypothetical_protein_protein	**2.72**
BCE_G9241_2579	*cypA*; cytochrome_P450	**4.73**
BCE_G9241_2580	transporter_putative	**5.65**
BCE_G9241_2581	streptogramin_A_acetyl_transferase	**6.27**
BCE_G9241_2590	macrolide_glycosyltransferase	**2.07**
BCE_G9241_2612	DegV_family_protein_putative	**2.08**
BCE_G9241_2654	outer_membrane_protein_romA	**2.36**
BCE_G9241_2656	YwqG-like_protein	**2.95**
BCE_G9241_2657	putative_esterase_family	**7.09**
BCE_G9241_2658	conserved_hypothetical_protein_protein	**5.82**
BCE_G9241_2675	conserved_hypothetical_protein_protein	**2.18**
BCE_G9241_2726	uridine_kinase_putative	**2.21**
BCE_G9241_2738	*rpiA*; ribose_5-phosphate_isomerase	**4.20**
BCE_G9241_2740	hypothetical_protein_protein	**17.84**
BCE_G9241_2741	chitin-binding_protein_putative	**18.66**
BCE_G9241_2762	Butirosin_biosynthesis_protein_BtrG	**2.19**
BCE_G9241_2788	conserved_hypothetical_protein_protein	**3.67**
BCE_G9241_2789	conserved_hypothetical_protein_protein	**2.82**
BCE_G9241_2800	cell_wall-associated_hydrolase	**3.71**
BCE_G9241_2801	mandelate_racemase/muconate_lactonizing_enzyme_family_protein	**4.47**
BCE_G9241_2812	X-pro_dipeptidyl-peptidase_putative	**2.16**

Table S5. Genes with increased expression in *B. cereus* G9241 in CO_2 (MGM + 0.8% bicarbonate)		
**SEQUENCE ID**	**GENE INFO**	**Fold difference**
BCE_G9241_2857	hypothetical_protein_protein	**6.04**
BCE_G9241_2871	hypothetical_protein_protein	**16.98**
BCE_G9241_2897	PTS_system_IIC_component_putative	**2.14**
BCE_G9241_2902	hypothetical_protein_protein	**2.81**
BCE_G9241_2903	transcriptional_regulator_putative	**2.81**
BCE_G9241_2904	hypothetical_protein_protein	**2.78**
BCE_G9241_2905	hypothetical_protein_protein	**3.13**
BCE_G9241_2936	fructose-bisphosphate_aldolase	**2.42**
BCE_G9241_2939	glyoxalase_family_protein_superfamily	**2.00**
BCE_G9241_2941	antibiotic_biosynthesis_monooxygenase_domain_protein	**3.64**
BCE_G9241_2971	*moxR*; ATPase_MoxR_family	**3.60**
BCE_G9241_2972	hypothetical_protein_secreted_protein	**3.31**
BCE_G9241_2973	hypothetical_protein_membrane_Spanning_protein	**3.16**
BCE_G9241_2986	*phnA*; alkylphosphonate_utilization_operon_protein_PhnA	**6.06**
BCE_G9241_2987	sulfate_permease_family_protein_VCA0077	**8.76**
BCE_G9241_2988	transcriptional_regulator_arsR_family	**4.33**
BCE_G9241_3046	short-chain-specific_acyl-CoA_dehydrogenase	**3.44**
BCE_G9241_3047	EpiH/GdmH-related_protein	**3.11**
BCE_G9241_3048	acetyltransferase_GNAT_family	**2.22**
BCE_G9241_3060	metal-dependent_hydrolase	**2.02**
BCE_G9241_3100	hydrolase_isochorismatase_family	**2.14**
BCE_G9241_3150	transposase_(IS4_family)_domain_protein	**2.25**
BCE_G9241_3151	conserved_hypothetical_protein_protein	**2.41**
BCE_G9241_3170	NADPH-cytochrome_P450_reductase	**2.07**
BCE_G9241_3187	putative_phosphohydrolases_Icc_family	**2.53**
BCE_G9241_3188	penicillin-binding_protein	**2.23**
BCE_G9241_3201	permease_putative	**4.69**
BCE_G9241_3208	MccC_family_protein	**2.19**
BCE_G9241_3211	conserved_hypothetical_protein_protein	**2.44**
BCE_G9241_3212	*serA*; D-isomer_specific_2-hydroxyacid_dehydrogenase_family_protein	**2.68**
BCE_G9241_3213	*serC*; phosphoserine_aminotransferase	**2.14**
BCE_G9241_3220	penicillin_G_acylase_putative	**2.49**
BCE_G9241_3227	internalin	**2.36**
BCE_G9241_3236	6-aminohexanoate-dimer_hydrolase	**3.10**
BCE_G9241_3249	SWIM_zinc_finger_family	**2.26**
BCE_G9241_3252	conserved_hypothetical_protein_protein	**4.71**
BCE_G9241_3278	conserved_hypothetical_protein_protein	**2.25**
BCE_G9241_3280	conserved_hypothetical_protein_protein	**2.83**
BCE_G9241_3309	collagen-like_triple_helix_repeat_protein	**2.06**
BCE_G9241_3317	conserved_hypothetical_protein_protein	**2.05**
BCE_G9241_3318	ATP_synthase_protein_I	**2.55**
BCE_G9241_3323	dTDP-glucose_46-dehydratase_putative	**2.05**
BCE_G9241_3325	CDP-4-dehydro-6-deoxy-D-gulose_4-reductase	**2.01**

Table S5. Genes with increased expression in *B. cereus* G9241 in CO_2 (MGM + 0.8% bicarbonate)		
**SEQUENCE ID**	**GENE INFO**	**Fold difference**
BCE_G9241_3395	AiiA-like_enzyme	**2.06**
BCE_G9241_3399	alcohol_dehydrogenase_zinc-containing	**2.92**
BCE_G9241_3412	ferrichrome_ABC_transporter_permease_protein	**2.35**
BCE_G9241_3425	acetyltransferase_GNAT_family	**2.01**
BCE_G9241_3460	hy	**3.33**
BCE_G9241_3477	hypothetical_protein_protein	**2.43**
BCE_G9241_3491	reticulocyte_binding_protein	**2.67**
BCE_G9241_3496	D-alanyl-D-alanine_carboxypeptidase	**2.50**
BCE_G9241_3500	*dhaS*; aldehyde_dehydrogenase_family_protein	**4.28**
BCE_G9241_3504	integral_membrane_protein	**2.21**
BCE_G9241_3543	ABC_transporter_permease_protein_putative	**2.43**
BCE_G9241_3544	ABC_transporter_ATP-binding_protein	**2.62**
BCE_G9241_3554	*nrdG*; anaerobic_ribonucleoside-triphosphate_reductase_activating_protein	**10.01**
BCE_G9241_3555	anaerobic_ribonucleoside-triphosphate_reductase_putative	**8.06**
BCE_G9241_3572	conserved_hypothetical_protein_protein	**2.47**
BCE_G9241_3573	spore_coat_protein_L-related_protein	**2.63**
BCE_G9241_3580	*fadA-2*; 3-ketoacyl-CoA_thiolase	**2.02**
BCE_G9241_3589	CsbD-like_family	**12.74**
BCE_G9241_3597	phospholipase/carboxylesterase_family_protein	**2.77**
BCE_G9241_3598	glyoxalase_family_protein_putative	**3.21**
BCE_G9241_3599	*opuE*; sodium/proline_symporter	**2.08**
BCE_G9241_3603	*carD*; CarD-like_transcriptional_regulator	**4.78**
BCE_G9241_3604	*hutG*; formiminoglutamase	**4.47**
BCE_G9241_3605	*hutI*; imidazolonepropionase	**7.65**
BCE_G9241_3606	*hutU*; urocanate_hydratase	**6.26**
BCE_G9241_3623	CcdC_protein	**3.12**
BCE_G9241_3624	cytochrome_c-type_biogenesis_protein_putative	**4.39**
BCE_G9241_3664	N-acetylmuramoyl-L-alanine_amidase	**3.13**
BCE_G9241_3672	*pXO1-18*; DNA_integration/recombination/inversion_protein	**3.85**
BCE_G9241_3673	hypothetical_protein_membrane_Spanning_protein	**2.06**
BCE_G9241_3695	copper-translocating_P-type_ATPase	**4.61**
BCE_G9241_3696	*pacS*; cation-transporting_ATPase_P-type	**6.80**
BCE_G9241_3700	IroE_protein	**2.78**
BCE_G9241_3736	phosphoglycerol_transferase	**8.40**
BCE_G9241_3797	YlxP-like_protein	**2.01**
BCE_G9241_3814	*codY*; transcriptional_regulator_CodY	**3.59**
BCE_G9241_3815	*hslU*; heat_shock_protein_HslVU_ATPase_subunit_HslU	**3.05**
BCE_G9241_3816	*clpQ*; heat_shock_protein_HslV	**2.50**
BCE_G9241_3820	MW1132	**4.23**
BCE_G9241_3834	*fabG*; 3-oxoacyl-(acyl-carrier-protein)_reductase	**2.40**
BCE_G9241_3866	hypothetical_protein_membrane_Spanning_protein	**2.60**
BCE_G9241_3931	cytochrome_c_oxidase_subunit_III	**3.72**
BCE_G9241_3932	*qoxA*; quinol_oxidase_subunit_I	**3.77**

Table S5. Genes with increased expression in *B. cereus* G9241 in CO_2 (MGM + 0.8% bicarbonate)		
**SEQUENCE ID**	**GENE INFO**	**Fold difference**
BCE_G9241_3933	cytochrome_c_oxidase_polypeptide_II	**3.06**
BCE_G9241_3935	*ctaA*; cytochrome_oxidase_assembly_protein	**2.02**
BCE_G9241_3996	*metE*; 5-methyltetrahydropteroyltriglutamate--_homocysteine_S-methyltransferase	**3.11**
BCE_G9241_4002	conserved_hypothetical_protein_protein	**9.65**
BCE_G9241_4042	conserved_hypothetical_protein_protein	**3.36**
BCE_G9241_4043	conserved_hypothetical_protein_protein	**6.54**
BCE_G9241_4108	alpha/beta_hydrolase	**2.12**
BCE_G9241_4117	*ribD*; riboflavin_biosynthesis_protein_RibD	**3.31**
BCE_G9241_4118	*ribE*; riboflavin_synthase_alpha_subunit	**4.17**
BCE_G9241_4119	*ribBA*; 34-dihydroxy-2-butanone_4-phosphate_synthase/GTP_cyclohydrolase_II	**4.95**
BCE_G9241_4120	*ribH*; 6, 7-dimethyl-8-ribityllumazine_synthase	**6.71**
BCE_G9241_4128	conserved_hypothetical_protein_protein	**2.13**
BCE_G9241_4133	transcriptional_regulator_MarR_family_putative	**2.56**
BCE_G9241_4173	MutT/nudix_family_protein	**2.04**
BCE_G9241_4195	cell_wall_surface_anchor_family_protein_putative	**2.48**
BCE_G9241_4196	transposase_IS4_family_putative	**4.52**
BCE_G9241_4249	hypothetical_protein_protein	**3.06**
BCE_G9241_4250	conserved_hypothetical_protein_protein	**9.61**
BCE_G9241_4260	conserved_hypothetical_protein_protein	**2.79**
BCE_G9241_4273	transposase_(IS4_family)_domain_protein	**2.07**
BCE_G9241_4308	superoxide_dismutase	**2.20**
BCE_G9241_4387	hypothetical_protein_membrane_Spanning_protein	**2.03**
BCE_G9241_4388	conserved_hypothetical_protein_protein	**2.75**
BCE_G9241_4389	spore_germination_protein_xc_precursor_bacillus	**3.70**
BCE_G9241_4393	transposase	**2.38**
BCE_G9241_4400	transposase_X	**7.66**
BCE_G9241_4406	*npr*; predicted_transposase	**3.34**
BCE_G9241_4508	*mreC*; rod_shape-determining_protein_MreC	**2.45**
BCE_G9241_4531	hypothetical_protein_protein	**6.45**
BCE_G9241_4532	conserved_hypothetical_protein_protein	**7.75**
BCE_G9241_4671	maoC_family_protein	**2.31**
BCE_G9241_4695	*pepQ*; proline_dipeptidase	**2.08**
BCE_G9241_4707	*ald*; alanine_dehydrogenase	**2.03**
BCE_G9241_4709	universal_stress_protein_family	**5.84**
BCE_G9241_4714	conserved_hypothetical_protein_protein	**2.41**
BCE_G9241_4715	cysteine-rich_protein	**3.14**
BCE_G9241_4729	*acs-6*; acetyl-CoA_synthetase	**4.67**
BCE_G9241_4753	*acuC*; acetoin_utilization_protein_AcuC	**5.25**
BCE_G9241_4754	hydrolase_alpha/beta_fold_family_putative	**3.42**
BCE_G9241_4782	nicotinate_phosphoribosyltransferase	**2.22**
BCE_G9241_4800	transcriptional_regulator_DeoR_family-related_protein	**5.08**
BCE_G9241_4810	*carD*; CarD-like_transcriptional_regulator	**2.70**

Table S5. Genes with increased expression in *B. cereus* G9241 in CO_2 (MGM + 0.8% bicarbonate)		
**SEQUENCE ID**	**GENE INFO**	**Fold difference**
BCE_G9241_4836	Na+/H+_antiporter_putative	**5.99**
BCE_G9241_4837	TrkA_C-terminal_domain_protein	**3.23**
BCE_G9241_4838	DNA_integration/recombination/inversion_protein	**2.31**
BCE_G9241_4913	conserved_hypothetical_protein_protein	**6.49**
BCE_G9241_4921	cell_surface_protein	**2.73**
BCE_G9241_4922	hypothetical_protein_protein	**2.08**
BCE_G9241_4923	conserved_hypothetical_protein_protein	**4.13**
BCE_G9241_4956	hypothetical_protein_protein	**4.38**
BCE_G9241_4957	hypothetical_protein_protein	**3.31**
BCE_G9241_5024	transcriptional_regulator_DeoR_family	**2.35**
BCE_G9241_5067	DNA-binding_protein	**2.07**
BCE_G9241_5069	cell_wall_endopeptidase_family_M23/M37	**2.41**
BCE_G9241_5102	*acd-7*; acyl-CoA_dehydrogenase	**7.44**
BCE_G9241_5103	hypothetical_protein_protein	**9.48**
BCE_G9241_5104	*atoB-4*; acetyl-CoA_acetyltransferase	**9.05**
BCE_G9241_5105	*hbd-1*; putative_3-hydroxyacyl-CoA_dehydrogenase_FadB	**12.11**
BCE_G9241_5106	hypothetical_protein_protein	**7.34**
BCE_G9241_5108	hypothetical_protein_protein	**4.17**
BCE_G9241_5109	D-alanyl-D-alanine_carboxypeptidase_putative	**2.48**
BCE_G9241_5133	DedA_family_protein_putative	**3.06**
BCE_G9241_5166	IG_hypothetical_protein_16995	**3.02**
BCE_G9241_5196	hypothetical_protein_protein	**3.32**
BCE_G9241_5199	iron_compound_ABC_transporter_ATP-binding_protein	**4.24**
BCE_G9241_5200	iron_compound_ABC_transporter_permease_protein	**3.81**
BCE_G9241_5201	iron_compound_ABC_transporter_permease_protein	**4.52**
BCE_G9241_5202	iron(III)_ABC_transporter_periplasmic_binding_protein_putative	**4.03**
BCE_G9241_5231	*crh*; phosphocarrier_protein_Hpr	**2.14**
BCE_G9241_5236	integral_membrane_protein	**2.11**
BCE_G9241_5273	ribosomal_subunit_interface_protein	**2.47**
BCE_G9241_5355	integral_membrane_protein	**4.25**
BCE_G9241_5423	phosphoglycerate_transporter_protein	**7.31**
BCE_G9241_5459	*alkK-2*; medium-chain_acyl-CoA_ligase	**4.33**
BCE_G9241_5469	NADH-ubiquinone_oxidoreductase_NQO10_subunit_(NQO10)_putative	**2.10**
BCE_G9241_5470	*nuoI*; NADH-ubiquinone_oxidoreductase_NQO9_subunit_(NQO9)	**2.78**
BCE_G9241_5515	transcriptional_regulator_tetR_family	**2.12**
BCE_G9241_5516	*acdA*; acyl-CoA_dehydrogenase_short-chain_specific	**2.44**
BCE_G9241_5531	collagen_adhesin_protein	**2.88**
BCE_G9241_5563	*fhuB*; ferrichrome_transport_permease_fhuB	**2.80**
BCE_G9241_5623	acriflavin_resistance_plasma_membrane_protein	**3.04**
BCE_G9241_CNI_0001	transposase_(IS4_family)_domain_protein	**2.03**
BCE_G9241_CNI_0048	*pXO1-80*; peptidase_M23/M37_family	**2.05**
BCE_G9241_CNI_0049	reticulocyte_binding_protein	**2.85**
BCE_G9241_CNI_0053	conserved_hypothetical_protein_protein	**2.42**

Table S5. Genes with increased expression in *B. cereus* G9241 in CO_2 (MGM + 0.8% bicarbonate)		
**SEQUENCE ID**	**GENE INFO**	**Fold difference**
BCE_G9241_CNI_0054	transposase_(IS4_family)_domain_protein	**2.29**
BCE_G9241_CNI_0055	acetyltransferase_GNAT_family_family	**2.06**
BCE_G9241_CNI_0123	transcriptional_regulator_GntR_family	**2.08**
BCE_G9241_CNI_0125	conserved_hypothetical_protein_protein	**8.91**
BCE_G9241_CNI_0139	main_capsid_protein_Gp34-like_protein	**2.32**
BCE_G9241_CNI_0141	protein_gp8	**2.63**
BCE_G9241_CNI_0142	gp9_putative	**2.41**
BCE_G9241_CNI_0143	conserved_hypothetical_protein_protein	**2.76**
BCE_G9241_CNI_0144	major_capsid_protein_gpP	**3.20**
BCE_G9241_CNI_0145	gp14_-like_protein	**3.25**
BCE_G9241_CNI_0152	major_capsid_protein_gpP	**3.21**
BCE_G9241_CNI_0154	protein_gp15	**3.19**
BCE_G9241_CNI_0155	*carB*; carbamoyl-phosphate_synthase_large_subunit_domain_protein	**3.15**
BCE_G9241_CNI_0166	conserved_hypothetical_protein_protein	**3.21**
BCE_G9241_CNI_0215	peptidase_M23/M37_family_putative	**2.19**
BCE_G9241_CNI_0216	glutaredoxin-like_protein_NrdH-related_protein	**2.68**
BCE_G9241_CNI_0217	conserved_hypothetical_protein_protein	**2.75**
BCE_G9241_CNI_0218	conserved_hypothetical_protein_protein	**2.96**
BCE_G9241_CNI_0219	hypothetical_protein_protein	**3.07**
BCE_G9241_CNI_0220	hypothetical_protein_protein	**3.11**
BCE_G9241_CNI_0222	conserved_hypothetical_protein_protein	**2.84**
BCE_G9241_CNI_0223	hypothetical_protein_protein	**3.04**
BCE_G9241_CNI_0224	conserved_hypothetical_protein_protein	**2.85**
BCE_G9241_CNI_0225	hypothetical_protein_protein	**2.50**
BCE_G9241_CNI_0248	transposase_for_insertion_sequence_element_is231e	**2.06**
BCE_G9241_CNI_0261	adhesin_protein	**3.69**
BCE_G9241_CNI_0265	acetyltransferase	**2.55**
BCE_G9241_CNI_0284	membrane_protein	**2.01**
BCE_G9241_CNI_0314	transposase_(IS4_family)_domain_protein	**2.10**
BCE_G9241_pBC218_0008	conserved_hypothetical_protein_protein	**8.62**
BCE_G9241_pBC218_0010	hypothetical_protein_protein	**47.05**
BCE_G9241_pBC218_0011	hypothetical_protein_protein	**50.77**
BCE_G9241_pBC218_0012	*pXO2-42*; S-layer_protein_/_peptidoglycan_endo-beta-N-_acetylglucosaminidase	**28.56**
BCE_G9241_pBC218_0013	periplasmic_component_of_efflux_system	**11.27**
BCE_G9241_pBC218_0015	phosphoglycerate_transporter_protein	**2.92**
BCE_G9241_pBC218_0017	ABC_transporter_ATP-binding_protein	**2.73**
BCE_G9241_pBC218_0018	*ybjZ*; ABC_transporter_ATP-binding_protein	**2.64**
BCE_G9241_pBC218_0020	DNA-binding_response_regulator_VicR	**2.09**
BCE_G9241_pBC218_0021	sensory_box_histidine_kinase_VicK_putative	**2.00**
BCE_G9241_pBC218_0024	protease_HhoA	**7.46**
BCE_G9241_pBC218_0025	hypothetical_protein_protein	**53.30**
BCE_G9241_pBC218_0026	protective_antigen	**40.13**
BCE_G9241_pBC218_0027	lethal_factor_precursor	**30.70**

Table S5. Genes with increased expression in *B. cereus* G9241 in CO_2 (MGM + 0.8% bicarbonate)		
**SEQUENCE ID**	**GENE INFO**	**Fold difference**
BCE_G9241_pBC218_0030	transposase	**2.09**
BCE_G9241_pBC218_0031	transposase_IS3_family_interruption	**3.06**
BCE_G9241_pBC218_0033	transposase_IS3_family_interruption	**3.29**
BCE_G9241_pBC218_0034	pVS1_resolvase	**6.11**
BCE_G9241_pBC218_0035	collagen_triple_helix_repeat_protein_putative	**2.02**
BCE_G9241_pBC218_0037	IS861_transposase_OrfB_putative	**2.06**
BCE_G9241_pBC218_0038	transposase	**2.54**
BCE_G9241_pBC218_0039	enterotoxin_B	**42.88**
BCE_G9241_pBC218_0040	non-hemolytic_enterotoxin_lytic_component_L1	**75.72**
BCE_G9241_pBC218_0042	non-hemolytic_enterotoxin_lytic_component_L2_putative	**24.70**
BCE_G9241_pBC218_0043	S-layer_homology_domain_protein	**41.00**
BCE_G9241_pBC218_0044	serine_protease_DO	**2.29**
BCE_G9241_pBC218_0045	*pXO1-109*; transcriptional_regulator_arsR_family	**54.40**
BCE_G9241_pBC218_0046	membrane_protein_putative	**77.91**
BCE_G9241_pBC218_0047	probable_electron_transfer_protein_Rv1937_putative	**89.28**
BCE_G9241_pBC218_0048	*cysK*; cysteine_synthase_A	**50.40**
BCE_G9241_pBC218_0049	*pXO2-61*; trans-acting_positive_regulator_(Atx_A)	**2.52**
BCE_G9241_pBC218_0051	transposase	**2.10**
BCE_G9241_pBC218_0055	*yngK-1*; YngK_protein	**6.87**
BCE_G9241_pBC218_0057	pXO1_ORF14-like_protein	**25.96**
BCE_G9241_pBC218_0058	collagen_adhesion_protein	**2.56**
BCE_G9241_pBC218_0059	polysaccharide_translocase_protein_putative	**39.13**
BCE_G9241_pBC218_0060	CMP-sialic_acid_synthetase_putative	**40.68**
BCE_G9241_pBC218_0061	UDP-N-acetylglucosamine_2-epimerase_putative	**44.33**
BCE_G9241_pBC218_0062	sialic_acid_synthase_putative	**18.34**
BCE_G9241_pBC218_0063	glycosyl_transferase_putative	**77.14**
BCE_G9241_pBC218_0064	*galU*; UTP-glucose-1-phosphate_uridylyltransferase	**42.26**
BCE_G9241_pBC218_0065	exopolysaccharide_biosynthesis_protein	**57.93**
BCE_G9241_pBC218_0066	*wzz*; chain_length_determinant_protein	**39.44**
BCE_G9241_pBC218_0067	transcriptional_regulator_LytR_family	**21.08**
BCE_G9241_pBC218_0068	hypothetical_protein_protein	**8.71**
BCE_G9241_pBC218_0069	polysaccharide_capsule_synthesis_protein	**22.30**
BCE_G9241_pBC218_0070	hypothetical_protein_protein	**13.21**
BCE_G9241_pBC218_0071	glycosyl_transferase_WecB/TagA/CpsF_family	**11.79**
BCE_G9241_pBC218_0072	*wzy*; polysaccharide_polymerase	**9.07**
BCE_G9241_pBC218_0073	glycosyl_transferase_group_2_family_protein	**15.82**
BCE_G9241_pBC218_0075	first_of_two_overlapping_orfs_with_similarity_to_IS3_genes	**10.51**
BCE_G9241_pBC218_0076	transposase	**10.37**
BCE_G9241_pBC218_0079	transposase_(IS4_family)	**7.00**
BCE_G9241_pBC218_0080	MAEBL_putative	**2.23**
BCE_G9241_pBC218_0081	*dnaX*; DNA_polymerase_III_gamma_and_tau_subunits	**7.30**
BCE_G9241_pBC218_0082	*tnpB*; transposase_all7245	**11.06**
BCE_G9241_pBC218_0084	DNA_polymerase_III_gamma_and_tau_subunits_putative	**2.25**
BCE_G9241_pBC218_0085	restriction_endonuclease_family	**2.88**

Table S5. Genes with increased expression in *B. cereus* G9241 in CO_2 (MGM + 0.8% bicarbonate)		
**SEQUENCE ID**	**GENE INFO**	**Fold difference**
BCE_G9241_pBC218_0086	SEC-C_motif_domain_protein	**2.05**
BCE_G9241_pBC218_0088	hypothetical_protein_protein	**4.36**
BCE_G9241_pBC218_0089	conserved_hypothetical_protein_protein	**2.02**
BCE_G9241_pBC218_0096	conserved_hypothetical_protein_protein_subfamily	**3.71**
BCE_G9241_pBC218_0097	hypothetical_protein_cytosolic_protein	**3.30**
BCE_G9241_pBC218_0098	hypothetical_protein_protein	**2.88**
BCE_G9241_pBC218_0099	hypothetical_protein_protein	**2.45**
BCE_G9241_pBC218_0104	geranylgeranyl_hydrogenase_BchP_putative	**7.68**
BCE_G9241_pBC218_0106	metallo-beta-lactamase_superfamily_protein	**2.84**
BCE_G9241_pBC218_0107	hypothetical_protein_protein	**6.12**
BCE_G9241_pBC218_0108	hypothetical_protein_protein	**2.68**
BCE_G9241_pBC218_0119	lipoprotein_NLP/P60_family	**28.54**
BCE_G9241_pBC218_0120	membrane_protein_putative	**22.39**
BCE_G9241_pBC218_0121	hypothetical_protein_protein	**6.17**
BCE_G9241_pBC218_0122	conserved_hypothetical_protein_protein	**3.10**
BCE_G9241_pBC218_0126	pXO1_ORF16-like_protein	**2.76**
BCE_G9241_pBC218_0128	*gnd*; 6-phosphogluconate_dehydrogenase_decarboxylating	**2.64**
BCE_G9241_pBC218_0132	pXO1_ORF14-like_protein	**2.03**
BCE_G9241_pBC218_0133	hypothetical_protein_protein	**3.20**
BCE_G9241_pBC218_0134	hypothetical_protein_protein	**3.44**
BCE_G9241_pBC218_0135	*pXO1-42*; conjugation_protein_TraG/TraD_family	**11.36**
BCE_G9241_pBC218_0136	hypothetical_protein_protein	**7.68**
BCE_G9241_pBC218_0141	DNA_primase	**2.77**
BCE_G9241_pBC218_0142	*tnpB*; transposase_all7245	**2.16**
BCE_G9241_pBC218_0147	surface_adhesion_protein_putative	**3.12**
BCE_G9241_pBC218_0150	*pXO1-23*; group_II_intron_reverse_transcriptase_maturase	**3.43**
BCE_G9241_pBC218_0154	conserved_hypothetical_protein_protein	**2.76**
BCE_G9241_pBC218_0155	conserved_hypothetical_protein_protein	**2.68**
BCE_G9241_pBC218_0174	conserved_hypothetical_protein_protein	**16.54**
BCE_G9241_pBC218_0175	hypothetical_protein_protein	**2.32**
BCE_G9241_pBClin29_0001	phage_putative_tail_component_N-terminal_domain_protein	**3.52**
BCE_G9241_pBClin29_0002	prophage_LambdaBa01_minor_structural_protein	**2.90**
BCE_G9241_pBClin29_0003	hypothetical_protein_protein	**2.63**
BCE_G9241_pBClin29_0004	XpaF1_protein	**2.73**
BCE_G9241_pBClin29_0028	transposase-like_protein_B	**2.84**
BCE_G9241_pBClin29_0029	phage_protein-related_protein	**3.15**
BCE_G9241_pBClin29_0036	IstB-like_ATP_binding_protein_family	**2.29**
BCE_G9241_pBClin29_0037	hypothetical_protein_protein	**2.53**
BCE_G9241_pBClin29_0038	phage_protein	**2.70**
BCE_G9241_pBCXO1_0008	conserved_hypothetical_protein_protein	**2.71**
BCE_G9241_pBCXO1_0023	conserved_hypothetical_protein_protein	**2.16**
BCE_G9241_pBCXO1_0030	conserved_hypothetical_protein_protein	**2.54**
BCE_G9241_pBCXO1_0045	transposase_(IS4_family)	**2.05**
BCE_G9241_pBCXO1_0061	conserved_hypothetical_protein_protein	**2.77**

Table S5. Genes with increased expression in *B. cereus* G9241 in CO_2 (MGM + 0.8% bicarbonate)		
**SEQUENCE ID**	**GENE INFO**	**Fold difference**
BCE_G9241_pBCXO1_0063	conserved_hypothetical_protein_protein	**2.40**
BCE_G9241_pBCXO1_0102	conserved_hypothetical_protein_protein	**2.09**
BCE_G9241_pBCXO1_0103	conserved_hypothetical_protein_protein	**4.64**
BCE_G9241_pBCXO1_0104	conserved_hypothetical_protein_protein	**3.39**
BCE_G9241_pBCXO1_0105	S-layer_homology_domain_protein	**12.59**
BCE_G9241_pBCXO1_0106	PAP2_superfamily_domain_protein	**9.54**
BCE_G9241_pBCXO1_0108	glycosyl_transferase_group_2_family_protein_domain_protein	**172.58**
BCE_G9241_pBCXO1_0109	*galU*; UTP-glucose-1-phosphate_uridylyltransferase	**226.82**
BCE_G9241_pBCXO1_0110	UDP-glucose/GDP-mannose_dehydrogenase_family_NAD_binding_domain_family	**123.46**
BCE_G9241_pBCXO1_0114	IS861_transposase_OrfB	**2.78**
BCE_G9241_pBCXO1_0115	conserved_hypothetical_protein_protein	**8.68**
BCE_G9241_pBCXO1_0116	conserved_hypothetical_protein_protein	**25.80**
BCE_G9241_pBCXO1_0117	conserved_hypothetical_protein_protein	**53.08**
BCE_G9241_pBCXO1_0118	conserved_hypothetical_protein_protein	**40.10**
BCE_G9241_pBCXO1_0119	*cya*; calmodulin-sensitive_adenylate_cyclase	**10.58**
BCE_G9241_pBCXO1_0120	*apt*; adenine_phosphoribosyltransferase	**27.10**
BCE_G9241_pBCXO1_0121	*apt*; adenine_phosphoribosyltransferase	**26.53**
BCE_G9241_pBCXO1_0126	conserved_hypothetical_protein_protein	**5.61**
BCE_G9241_pBCXO1_0128	conserved_hypothetical_protein_protein	**2.03**
