# Supplementary material for: Comparative Transcriptional Profiling of Bacillus cereus Sensu Lato Strains during Growth in CO2-Bicarbonate and Aerobic Atmospheres
Source: PLoS One. 2009 Mar 19;4(3):e4904. doi: 10.1371/journal.pone.0004904 (PMC2654142; doi:10.1371/journal.pone.0004904)
Supplement: Table S6 — Genes with increased expression in B. cereus G9241 in MGM in O2 (0.30 MB PDF) [file pone.0004904.s006.pdf]

Table S6. Genes with increased expression in *B. cereus* G9241 in MGM in O_2		
**SEQUENCE ID**	**GENE INFO**	**Fold difference**
BCE_G9241_0025	Orn/Lys/Arg_decarboxylase	**4.02**
BCE_G9241_0034	*metS*; methionyl-tRNA_synthetase	**5.03**
BCE_G9241_0035	*metS*; methionyl-tRNA_synthetase	**2.07**
BCE_G9241_0042	*ispE*; 4-diphosphocytidyl-2C-methyl-D-erythritolkinase	**3.17**
BCE_G9241_0058	S1_RNA-binding_domain_protein	**2.20**
BCE_G9241_0064	transcription_activator_probable_Baf_family	**3.49**
BCE_G9241_0065	chaperonin_33_kDa	**2.48**
BCE_G9241_0067	anthranilate_synthase_component_I	**2.02**
BCE_G9241_0079	*ctsR*; transcriptional_regulator_ctsR	**3.54**
BCE_G9241_0080	ClpC_ATPase	**2.50**
BCE_G9241_0087	*ispF*; 2C-methyl-D-erythritol_24-cyclodiphosphate_synthase	**2.33**
BCE_G9241_0095	*nusG*; transcription_termination/antitermination_factor_nusG	**2.15**
BCE_G9241_0131	*rplM*; ribosomal_protein_L13	**2.23**
BCE_G9241_0132	*rpsI*; ribosomal_protein_S9	**2.31**
BCE_G9241_0140	glycerate_kinase	**2.05**
BCE_G9241_0141	*rocF*; arginase	**2.27**
BCE_G9241_0142	conserved_hypothetical_protein_protein_TIGR00159	**2.06**
BCE_G9241_0147	peptidase_putative	**2.83**
BCE_G9241_0151	conserved_hypothetical_protein_protein	**3.46**
BCE_G9241_0152	sigma-70_region_2_family	**4.41**
BCE_G9241_0161	oxidoreductase_FAD-binding_putative	**2.57**
BCE_G9241_0162	multi-drug_resistance_protein_putative	**3.02**
BCE_G9241_0181	*proC*; pyrroline-5-carboxylate_reductase	**2.69**
BCE_G9241_0187	*modA*; molybdenum_ABC_transporter_periplasmic_molybdate-binding_protein	**2.67**
BCE_G9241_0188	molybdenum_cofactor_biosynthesis_protein_putative	**2.88**
BCE_G9241_0197	YxkD-like_protein	**3.62**
BCE_G9241_0204	galactose-1-phosphate_uridylyltransferase	**3.68**
BCE_G9241_0222	hydrolase_haloacid_dehalogenase-like_family	**2.93**
BCE_G9241_0235	D-alanine--D-alanine_ligase	**5.18**
BCE_G9241_0236	*murF*; UDP-N-acetylmuramoylalanyl-D-glutamyl-2_6-_diaminopimelate--D-alanyl-D-alanyl_ligase	**2.29**
BCE_G9241_0240	*acpS*; holo-(acyl-carrier-protein)_synthase	**4.21**
BCE_G9241_0241	conserved_hypothetical_protein_protein	**2.56**
BCE_G9241_0242	*alr*; alanine_racemase	**2.01**
BCE_G9241_0262	*guaA*; GMP_synthase	**2.01**
BCE_G9241_0264	DNA-binding_response_regulator	**2.21**
BCE_G9241_0267	alpha/beta_hydrolase	**2.48**
BCE_G9241_0282	*purC*; phosphoribosylaminoimidazole-succinocarboxamidesynthase	**2.07**
BCE_G9241_0295	conserved_hypothetical_protein_protein_TIGR00265	**2.48**
BCE_G9241_0299	hypothetical_protein_membrane_Spanning_protein	**2.40**
BCE_G9241_0314	*gatC*; glutamyl-tRNA(gln)_amidotransferase_C_subunit	**2.92**
BCE_G9241_0324	*ampS*; aminopeptidase	**2.82**
BCE_G9241_0340	*amhX*; amidohydrolase_amhX	**3.91**

Table S6. Genes with increased expression in *B. cereus* G9241 in MGM in O_2		
**SEQUENCE ID**	**GENE INFO**	**Fold difference**
BCE_G9241_0344	5-methylthioribose_kinase	**2.12**
BCE_G9241_0373	methyl-accepting_chemotaxis_protein_putative	**3.40**
BCE_G9241_0376	*malA*; alpha-glucosidase	**2.74**
BCE_G9241_0377	PTS_system_glucose-specific_IIBC_component	**4.79**
BCE_G9241_0378	PTS_system_IIBC_component	**3.82**
BCE_G9241_0384	anaerobic_C4-dicarboxylate_transporter	**2.78**
BCE_G9241_0386	uncharacterized_protein_from_YqgV/UPF0045_family	**2.28**
BCE_G9241_0387	ABC_transporter_permease_protein_cysTW_family	**2.67**
BCE_G9241_0388	pyrimidine_precursor_biosynthesis_enzyme_putative	**2.31**
BCE_G9241_0392	hypothetical_protein_protein	**5.40**
BCE_G9241_0395	thioredoxin	**2.95**
BCE_G9241_0396	conserved_hypothetical_protein_protein	**3.32**
BCE_G9241_0420	*cadA*; cadmium-translocating_P-type_ATPase	**2.94**
BCE_G9241_0432	thioredoxin-like_oxidoreductases	**3.47**
BCE_G9241_0433	conserved_hypothetical_protein_protein	**3.00**
BCE_G9241_0448	glutaminase_A	**2.85**
BCE_G9241_0474	*mutY*; A/G-specific_adenine_glycosylase	**2.12**
BCE_G9241_0478	protein_of_unknown_function_(DUF402)_family	**3.23**
BCE_G9241_0485	daunorubicin_resistance_ATP-binding_protein_drrA	**6.30**
BCE_G9241_0486	membrane_protein_putative	**4.83**
BCE_G9241_0487	ABC_transporter_permease_protein	**3.24**
BCE_G9241_0488	LCTB_protein	**4.30**
BCE_G9241_0491	*fur-3*; transcriptional_regulator_Fur_family	**2.34**
BCE_G9241_0497	conserved_hypothetical_protein_protein_TIGR01033	**4.29**
BCE_G9241_0503	RNA_methyltransferase_TrmH_family_group_2	**4.18**
BCE_G9241_0504	sensory_box_protein/GGDEF_domain_protein	**2.09**
BCE_G9241_0530	accessory_gene_regulator_protein_C_putative	**4.83**
BCE_G9241_0531	*agrA*; accessory_gene_regulator_protein_A	**2.72**
BCE_G9241_0535	microbial_collagenase	**63.51**
BCE_G9241_0538	*mcpB*; methyl-accepting_chemotaxis_protein	**2.53**
BCE_G9241_0550	methyl-accepting_chemotaxis_transducer_putative	**5.03**
BCE_G9241_0559	acetyltransferase_GNAT_family_putative	**3.21**
BCE_G9241_0563	acetyltransferase_GNAT_family_putative	**3.54**
BCE_G9241_0572	nicotinate_phosphoribosyltransferase_putative	**3.32**
BCE_G9241_0573	transcriptional_activator_NprR	**6.23**
BCE_G9241_0574	hypothetical_protein_protein	**5.16**
BCE_G9241_0577	hypothetical_protein_exported_protein	**3.61**
BCE_G9241_0582	glyoxylase_I_family_protein_VCA0890	**2.30**
BCE_G9241_0600	NorQ_protein	**2.20**
BCE_G9241_0606	*arcD*; arginine/ornithine_antiporter	**2.09**
BCE_G9241_0608	*treP*; PTS_system_trehalose-specific_IIBC_component	**84.83**
BCE_G9241_0609	trehalose-6-phosphate_hydrolase	**47.16**
BCE_G9241_0615	ATPase_histidine_kinase-_DNA_gyrase_B-_and_HSP90-like_domain_protein_protein	**3.32**

Table S6. Genes with increased expression in *B. cereus* G9241 in MGM in O_2		
**SEQUENCE ID**	**GENE INFO**	**Fold difference**
BCE_G9241_0616	*vraR*; DNA-binding_response_regulator	**2.33**
BCE_G9241_0644	*glpT*; glycerol-3-phosphate_transporter	**26.69**
BCE_G9241_0645	transcriptional_regulator_MarR_family	**8.47**
BCE_G9241_0646	drug_transport_protein_putative	**5.73**
BCE_G9241_0647	ribose_operon_repressor_RbsR	**4.23**
BCE_G9241_0648	*rbsK*; ribokinase	**3.97**
BCE_G9241_0651	*rbsC*; ribose_ABC_transporter_permease_protein	**3.66**
BCE_G9241_0652	*rbsB*; periplasmic_sugar-binding_proteins	**2.72**
BCE_G9241_0653	transaldolase_putative	**2.94**
BCE_G9241_0654	proteinase_VCA0223	**212.16**
BCE_G9241_0658	phospholipase_c_precursor	**200.54**
BCE_G9241_0659	*AA1-330*; phospholipase_c_precursor	**150.93**
BCE_G9241_0660	conserved_hypothetical_protein_protein	**3.71**
BCE_G9241_0665	methyl-accepting_chemotaxis_protein_putative	**4.43**
BCE_G9241_0669	sortase_family_protein_putative	**3.40**
BCE_G9241_0670	di-/tripeptide_transporter	**6.50**
BCE_G9241_0675	IG_hypothetical_protein_22578	**3.41**
BCE_G9241_0677	xanthine/uracil_permeases_family_protein	**2.12**
BCE_G9241_0679	conserved_hypothetical_protein_protein	**4.44**
BCE_G9241_0681	Na+/H+_antiporter_putative	**4.58**
BCE_G9241_0724	hypothetical_protein_protein	**4.15**
BCE_G9241_0725	*kdpA*; potassium-transporting_ATPase_A_subunit	**4.44**
BCE_G9241_0726	hypothetical_protein_protein	**6.64**
BCE_G9241_0727	*kdpB*; potassium-translocating_P-type_ATPase_B_subunit	**4.09**
BCE_G9241_0728	*kdpC*; potassium-transporting_ATPase_C_subunit	**2.96**
BCE_G9241_0739	transposase	**2.07**
BCE_G9241_0763	rhodanese-like_domain_protein	**2.00**
BCE_G9241_0764	hydroxyacylglutathione_hydrolase	**2.71**
BCE_G9241_0771	conserved_hypothetical_protein_protein	**2.98**
BCE_G9241_0789	hypothetical_protein_membrane_Spanning_protein	**2.74**
BCE_G9241_0810	conserved_hypothetical_protein_protein	**2.57**
BCE_G9241_0812	glucokinase_regulator-related_protein	**5.15**
BCE_G9241_0813	PTS_system_IIBC_components	**3.33**
BCE_G9241_0814	hypothetical_protein_protein	**2.90**
BCE_G9241_0851	NADP-dependent_glyceraldehyde-3-phosphate_dehydrogenase	**6.05**
BCE_G9241_0890	*pXO2-42*; S-layer_protein_precursor	**3.18**
BCE_G9241_0893	*algI*; alginate_O-acetylation_protein_algI	**2.38**
BCE_G9241_0897	hypothetical_protein_protein	**2.61**
BCE_G9241_0898	hypothetical_protein_protein	**3.48**
BCE_G9241_0899	predicted_protein	**3.49**
BCE_G9241_0902	*pXO2-42*; surface-layer_N-acetylmuramoyl-L-alanine_amidase	**2.51**
BCE_G9241_0909	hypothetical_protein_protein	**5.94**
BCE_G9241_0910	hypothetical_protein_protein	**10.95**
BCE_G9241_0911	limonene_cyclase	**6.31**

Table S6. Genes with increased expression in *B. cereus* G9241 in MGM in O_2		
**SEQUENCE ID**	**GENE INFO**	**Fold difference**
BCE_G9241_0912	Sb42_putative	**9.07**
BCE_G9241_0913	replicative_DNA_helicase_putative	**5.18**
BCE_G9241_0918	hypothetical_protein_protein	**2.08**
BCE_G9241_0919	hypothetical_protein_protein	**37.65**
BCE_G9241_0920	putative_peptidoglycan_binding_domain_protein	**9.91**
BCE_G9241_0921	hypothetical_protein_protein	**3.57**
BCE_G9241_0926	*atl*; bifunctional_autolysin	**4.56**
BCE_G9241_0931	putative_peptidoglycan_binding_domain_protein	**18.56**
BCE_G9241_0932	hypothetical_protein_protein	**2.57**
BCE_G9241_0940	5-methylcytosine-specific_restriction_related_enzyme	**2.60**
BCE_G9241_0944	conserved_hypothetical_protein_protein	**2.48**
BCE_G9241_0948	hypothetical_protein_protein	**2.33**
BCE_G9241_0961	hypothetical_protein_membrane_Spanning_protein	**2.81**
BCE_G9241_0963	*carB*; carbamoyl-phosphate_synthase_large_subunit	**3.55**
BCE_G9241_0996	S-layer_homology_domain	**28.32**
BCE_G9241_1003	hypothetical_protein_protein	**2.36**
BCE_G9241_1010	hypothetical_protein_membrane_Spanning_protein	**9.27**
BCE_G9241_1012	rsbV	**3.71**
BCE_G9241_1013	*rsbW*; anti-sigma_B_factor	**3.55**
BCE_G9241_1014	sigma-B	**2.58**
BCE_G9241_1015	bacterioferritin_putative	**2.95**
BCE_G9241_1044	*glpP*; glycerol_uptake_operon_antiterminator_regulatory_protein	**4.62**
BCE_G9241_1045	*glpD*; aerobic_glycerol-3-phosphate_dehydrogenase	**9.80**
BCE_G9241_1048	transcriptional_regulator_PadR_family_domain_protein	**2.41**
BCE_G9241_1059	ABC_transporter_ATP-binding_protein	**2.57**
BCE_G9241_1060	membrane_protein_putative	**2.09**
BCE_G9241_1095	leukotoxin_putative	**29.98**
BCE_G9241_1112	membrane_protein_putative	**2.96**
BCE_G9241_1126	signal_peptidase_I	**2.13**
BCE_G9241_1165	hydrolase	**3.03**
BCE_G9241_1178	*trpS*; tryptophanyl-tRNA_synthetase	**2.78**
BCE_G9241_1186	MATE_efflux_family_protein	**3.07**
BCE_G9241_1187	*pXO2-66*; pheromone_binding_protein	**2.07**
BCE_G9241_1192	negative_regulator_of_competence_mecA_putative	**2.40**
BCE_G9241_1198	YjbH-like_protein	**4.41**
BCE_G9241_1200	adenylate_cyclase	**2.39**
BCE_G9241_1201	IG_hypothetical_protein_17542	**2.58**
BCE_G9241_1240	*trpE*; anthranilate_synthase_component_I	**5.50**
BCE_G9241_1241	*trpGD*; anthranilate_synthase_component_II	**4.81**
BCE_G9241_1242	*trpD*; anthranilate_phosphoribosyltransferase	**4.47**
BCE_G9241_1243	*trpC*; indole-3-glycerol_phosphate_synthase	**5.23**
BCE_G9241_1244	hypothetical_protein_protein	**5.40**
BCE_G9241_1245	*trpF*; N-(5'phosphoribosyl)anthranilate_isomerase	**3.85**
BCE_G9241_1246	*trpB*; tryptophan_synthase_beta_subunit	**5.97**

Table S6. Genes with increased expression in *B. cereus* G9241 in MGM in O_2		
**SEQUENCE ID**	**GENE INFO**	**Fold difference**
BCE_G9241_1247	*trpA*; tryptophan_synthase_alpha_subunit	**2.72**
BCE_G9241_1271	conserved_hypothetical_protein_protein	**2.09**
BCE_G9241_1313	transcriptional_regulator_GntR_family_putative	**2.97**
BCE_G9241_1314	cysteine-rich_domain_family	**9.14**
BCE_G9241_1315	iron-sulfur_cluster_binding_protein	**4.38**
BCE_G9241_1316	YkgG_family_protein	**4.27**
BCE_G9241_1317	prepilin_peptidase_type_IV	**2.58**
BCE_G9241_1366	ribonucleotide-diphosphate_reductase_alpha_chain	**2.08**
BCE_G9241_1369	transcriptional_regulator_GntR_family	**2.20**
BCE_G9241_1370	ABC_transporter_ATP-binding_protein_(yhcG)	**2.36**
BCE_G9241_1371	ABC_transporter_permease_protein_putative	**2.49**
BCE_G9241_1373	bacitracin_transport_permease_protein_BCRB	**2.09**
BCE_G9241_1549	hypothetical_protein_membrane_Spanning_protein	**2.03**
BCE_G9241_1555	Lmbe-related_protein	**2.19**
BCE_G9241_1632	lacX_protein	**2.32**
BCE_G9241_1636	hypothetical_protein_cytosolic_protein	**2.16**
BCE_G9241_1637	cold_shock_protein-related_protein	**3.32**
BCE_G9241_1667	hydrolase	**2.33**
BCE_G9241_1669	sodium-driven_polar_flagellar_protein_PomB_putative	**3.48**
BCE_G9241_1670	*cheY*; chemotaxis_response_regulator	**6.32**
BCE_G9241_1671	*cheA*; histidine_kinase_(cheA)	**6.42**
BCE_G9241_1672	chemotaxis_protein_cheC	**4.21**
BCE_G9241_1673	conserved_hypothetical_protein_protein	**3.23**
BCE_G9241_1674	conserved_hypothetical_protein_protein	**2.78**
BCE_G9241_1675	chemotaxis_protein_methyltransferase_putative	**2.43**
BCE_G9241_1676	conserved_hypothetical_protein_protein	**2.29**
BCE_G9241_1678	conserved_hypothetical_protein_protein	**8.35**
BCE_G9241_1679	flagellar_hook-associated_protein_1_putative	**5.64**
BCE_G9241_1680	flagellar_hook-associated_protein_flgL_putative	**5.56**
BCE_G9241_1681	*fliD*; flagellar_hook-associated_protein_2	**5.25**
BCE_G9241_1682	flagellar_protein_putative	**5.07**
BCE_G9241_1683	conserved_hypothetical_protein_protein	**4.69**
BCE_G9241_1684	*flgB*; flagellar_basal-body_rod_protein_flgB	**12.05**
BCE_G9241_1685	*flgC*; flagellar_basal-body_rod_protein_flgC	**9.78**
BCE_G9241_1686	flagellar_hook-basal_body_complex_protein_fliE	**15.92**
BCE_G9241_1687	flagellar_M-ring_protein_putative	**12.91**
BCE_G9241_1688	*fliG*; flagellar_motor_switch_protein_fliG	**6.45**
BCE_G9241_1689	conserved_hypothetical_protein_protein	**4.93**
BCE_G9241_1690	*fliI*; type_III_secretion_cytoplasmic_ATPase_SctN_TC0040	**4.64**
BCE_G9241_1691	*xcpC*; hypothetical_protein_cytosolic_protein	**3.44**
BCE_G9241_1692	conserved_hypothetical_protein_protein	**5.06**
BCE_G9241_1693	Basal-body_rod_modification_protein_flgD	**6.29**
BCE_G9241_1694	flagellar_hook_protein_flgE_putative	**9.79**
BCE_G9241_1695	conserved_hypothetical_protein_protein	**4.57**

Table S6. Genes with increased expression in *B. cereus* G9241 in MGM in O_2		
**SEQUENCE ID**	**GENE INFO**	**Fold difference**
BCE_G9241_1696	conserved_hypothetical_protein_protein	**3.19**
BCE_G9241_1697	chemotaxis_protein_cheV_VC1602	**5.01**
BCE_G9241_1700	flagellin	**5.20**
BCE_G9241_1701	flagellin	**7.19**
BCE_G9241_1702	flagellin	**7.47**
BCE_G9241_1703	flagellin	**9.02**
BCE_G9241_1704	transglycosylase_SLT_domain_protein	**4.49**
BCE_G9241_1705	flagellar_motor_switch_protein_fliN_VC2125_putative	**6.41**
BCE_G9241_1706	flagellar_motor_switch_protein_(fliM)_putative	**6.41**
BCE_G9241_1707	flagellar_motor_switch_protein_fliN_putative	**5.93**
BCE_G9241_1708	flagellar_motor_switch_protein_fliN	**7.29**
BCE_G9241_1709	*fliP*; flagellar_biosynthetic_protein_fliP	**6.48**
BCE_G9241_1710	*fliQ*; flagellar_biosynthesis_protein_fliQ	**3.97**
BCE_G9241_1711	flagellar_biosynthetic_protein_fliR_putative	**7.96**
BCE_G9241_1712	*flhB*; flagellar_biosynthesis_protein_flhB	**7.11**
BCE_G9241_1713	*flhA*; flagellar_biosynthesis_protein_flhA	**5.45**
BCE_G9241_1714	flagellar_biosynthetic_protein_flhF_putative	**3.48**
BCE_G9241_1715	flagellar_basal-body_rod_protein_flgF_putative	**3.14**
BCE_G9241_1718	AzlC_family_protein	**2.00**
BCE_G9241_1719	branched-chain_amino_acid_transport_protein_azlD	**2.16**
BCE_G9241_1734	permease_putative	**2.08**
BCE_G9241_1753	conserved_hypothetical_protein_protein	**5.94**
BCE_G9241_1769	*nhaC*; Na+/H+_antiporter_NhaC	**3.82**
BCE_G9241_1777	transcriptional_regulator	**4.30**
BCE_G9241_1778	conserved_hypothetical_protein_integral_membrane_protein_putative	**2.24**
BCE_G9241_1790	hypothetical_protein_membrane_Spanning_protein	**2.73**
BCE_G9241_1791	multi-TM2_domain_protein	**3.92**
BCE_G9241_1792	conserved_hypothetical_protein_protein	**6.01**
BCE_G9241_1793	RNA_polymerase_sigma-24_factor_putative	**3.00**
BCE_G9241_1803	*gltT*; proton/sodium-glutamate_symport_protein	**46.21**
BCE_G9241_1804	*aspA*; aspartate_ammonia-lyase	**24.83**
BCE_G9241_1805	*aspA*; aspartate_ammonia-lyase	**8.88**
BCE_G9241_1806	*malS*; malate_oxidoreductase_VC1188	**15.88**
BCE_G9241_1807	sensor_histidine_kinase_putative	**16.00**
BCE_G9241_1808	response_regulator_putative	**10.42**
BCE_G9241_1812	*asnA*; aspartate--ammonia_ligase	**2.79**
BCE_G9241_1834	acetyltransferase_GNAT_family	**2.09**
BCE_G9241_1838	sodium-_and_chloride-dependent_transporter	**4.98**
BCE_G9241_1846	*msrA*; peptide_methionine_sulfoxide_reductase	**2.98**
BCE_G9241_1847	*ilvE*; branched-chain_amino_acid_aminotransferase	**25.27**
BCE_G9241_1848	*ilvB*; acetolactate_synthase_large_subunit_biosynthetic_type	**22.92**
BCE_G9241_1849	*ilvN*; acetolactate_synthase_III_small_chain_VC2482	**20.06**
BCE_G9241_1850	*ilvC*; ketol-acid_reductoisomerase	**23.77**
BCE_G9241_1851	*ilv*D;dihydroxy-acid_dehydratase	**20.56**

**Table S6. Genes with increased expression in *B. cereus* G9241 in MGM in O_2**		
**SEQUENCE ID**	**GENE INFO**	**Fold difference**
BCE_G9241_1852	*ilvA*; threonine_dehydratase	**7.02**
BCE_G9241_1854	uncharacterized_membrane_protein_possible_Na+_channel_or_pump	**2.21**
BCE_G9241_1861	*abfT-1*; acetyl-CoA_hydrolase/transferase_family_protein	**2.22**
BCE_G9241_1875	membrane_protein_putative	**9.61**
BCE_G9241_1876	non-hemolytic_enterotoxin_lytic_component_L2	**39.16**
BCE_G9241_1877	enterotoxin_A	**61.32**
BCE_G9241_1878	non-hemolytic_enterotoxin_lytic_component_L1	**28.18**
BCE_G9241_1879	non-expressed_enterotoxin_C	**58.60**
BCE_G9241_1880	transporter_LysE_family	**19.00**
BCE_G9241_1976	oxidoreductase_short_chain_dehydrogenase/reductase_family_superfamily	**2.71**
BCE_G9241_1977	hypothetical_protein_protein	**2.49**
BCE_G9241_2012	transglutaminase-like_predicted_protease	**3.65**
BCE_G9241_2029	conserved_hypothetical_protein_protein	**2.46**
BCE_G9241_2041	adhesion_lipoprotein	**4.33**
BCE_G9241_2044	xenobiotic_reductase_A	**2.57**
BCE_G9241_2057	ABC_transporter_permease_protein_putative	**2.73**
BCE_G9241_2090	acetyltransferase_GNAT_family_putative	**2.22**
BCE_G9241_2095	transcriptional_regulator_GntR_family	**2.57**
BCE_G9241_2100	conserved_hypothetical_protein_protein	**2.01**
BCE_G9241_2111	conserved_hypothetical_protein_protein	**2.85**
BCE_G9241_2123	RNA_polymerase_sigma-70_factor_ECF_subfamily_putative	**2.26**
BCE_G9241_2128	conserved_hypothetical_protein_protein	**6.73**
BCE_G9241_2178	*aspS*; aspartyl-tRNA_synthetase	**3.40**
BCE_G9241_2212	DegV_family_protein	**2.13**
BCE_G9241_2216	asparagine_synthase_(glutamine-hydrolyzing)	**2.23**
BCE_G9241_2235	*gntK*; gluconate_kinase	**2.41**
BCE_G9241_2236	gluconate_permease	**2.06**
BCE_G9241_2242	macrolide-efflux_protein	**2.78**
BCE_G9241_2246	*proV*; glycine_betaine/carnitine/choline_ABC_transporter_ATP-binding_protein	**2.35**
BCE_G9241_2324	hypothetical_protein_cytosolic_protein-related_protein	**2.20**
BCE_G9241_2328	*pXO1-109*; transcriptional_repressor	**3.85**
BCE_G9241_2332	oxalate/formate_antiporter_putative	**19.88**
BCE_G9241_2355	transporter_putative	**2.69**
BCE_G9241_2356	oligopeptide_transport_system_permease_protein_oppB	**2.35**
BCE_G9241_2361	conserved_hypothetical_protein_protein	**4.09**
BCE_G9241_2362	conserved_hypothetical_protein_protein	**4.17**
BCE_G9241_2381	hydrolase_alpha/beta_fold_family_putative	**3.33**
BCE_G9241_2382	*hemG*; protoporphyrinogen_oxidase	**2.60**
BCE_G9241_2402	acetyltransferase	**2.19**
BCE_G9241_2499	*acdA*; acyl-CoA_dehydrogenase_short-chain_specific	**2.61**
BCE_G9241_2510	uridine_kinase_putative	**2.86**
BCE_G9241_2515	*drrA*; DNA-binding_response_regulator	**2.58**

Table S6. Genes with increased expression in *B. cereus* G9241 in MGM in O_2		
**SEQUENCE ID**	**GENE INFO**	**Fold difference**
BCE_G9241_2518	*mtn*; S-adenosylhomocysteine_nucleosidase	**2.92**
BCE_G9241_2519	conserved_hypothetical_protein_protein	**3.08**
BCE_G9241_2522	bacillolysin	**2.21**
BCE_G9241_2525	Aha1_domain_superfamily	**3.56**
BCE_G9241_2545	*murE*; UDP-N-acetylmuramoylalanyl-D-glutamyl-2_6-_diaminopimelate_ligase	**2.93**
BCE_G9241_2563	*hom*; homoserine_dehydrogenase_(hom)	**3.47**
BCE_G9241_2605	oxidoreductase_zinc-binding_putative	**2.33**
BCE_G9241_2621	*pulA*; alpha-dextran_endo-16-alpha-glucosidase	**3.23**
BCE_G9241_2630	LAAC	**2.59**
BCE_G9241_2645	beta-lactamase	**2.13**
BCE_G9241_2650	nudix/MutT_family_protein	**2.26**
BCE_G9241_2663	acetyltransferase_GNAT_family_putative	**2.76**
BCE_G9241_2664	probable_electron_transfer_protein_Rv1937_putative	**2.15**
BCE_G9241_2676	bacillolysin	**86.91**
BCE_G9241_2693	MW2053	**2.56**
BCE_G9241_2701	6-aminohexanoate-dimer_hydrolase	**2.68**
BCE_G9241_2746	ubiquinone/menaquinone_biosynthesis_methyltransferase_UBIE	**3.81**
BCE_G9241_2747	YjbR_protein_putative	**4.06**
BCE_G9241_2748	acetyltransferase_GNAT_family_family	**3.83**
BCE_G9241_2761	N-methyl-transferase-related_protein	**2.25**
BCE_G9241_2769	conserved_hypothetical_protein_protein	**2.51**
BCE_G9241_2797	helix-turn-helix_domain_protein	**2.15**
BCE_G9241_2828	DegV_family_protein	**2.83**
BCE_G9241_2838	conserved_hypothetical_protein_protein	**3.07**
BCE_G9241_2915	*tyrA*; prephenate_dehydrogenase	**4.75**
BCE_G9241_2916	*hisC*; histidinol-phosphate_aminotransferase	**7.11**
BCE_G9241_2917	*aroC*; chorismate_synthase	**5.46**
BCE_G9241_2918	MW1680	**3.68**
BCE_G9241_2947	*proB*; glutamate_5-kinase	**2.47**
BCE_G9241_2967	glyoxylase_family_protein_putative	**2.07**
BCE_G9241_2993	conserved_hypothetical_protein_protein	**7.31**
BCE_G9241_2994	MutT/nudix_family_protein_putative	**10.85**
BCE_G9241_2997	conserved_hypothetical_protein_protein	**5.17**
BCE_G9241_2998	ABC_transporter_ATP-binding_protein	**6.50**
BCE_G9241_2999	transcription_regulator_(GntR_family)_BH0651	**4.23**
BCE_G9241_3015	hypothetical_protein_cytosolic_protein	**2.43**
BCE_G9241_3020	HesB-like_protein	**2.68**
BCE_G9241_3066	*aspA*; aspartate_ammonia-lyase	**13.22**
BCE_G9241_3067	L-asparaginase_I_putative	**2.67**
BCE_G9241_3074	hemolysin_BL_binding_component_precursor	**28.70**
BCE_G9241_3075	Hbl_B_protein	**129.93**
BCE_G9241_3076	hemolysin_BL_lytic_component_L1	**52.52**
BCE_G9241_3077	hemolysin_BL_lytic_component_L2	**33.91**

Table S6. Genes with increased expression in *B. cereus* G9241 in MGM in O_2		
**SEQUENCE ID**	**GENE INFO**	**Fold difference**
BCE_G9241_3082	amino_acid_permease	**2.01**
BCE_G9241_3083	*proC*; pyrroline-5-carboxylate_reductase	**2.22**
BCE_G9241_3089	sensor_histidine_kinase_putative	**2.67**
BCE_G9241_3090	glutaminase_A	**3.62**
BCE_G9241_3091	sodium:alanine_symporter_family_protein	**8.13**
BCE_G9241_3096	*ushA*; 5'-nucleotidase	**2.41**
BCE_G9241_3132	*menA*; 1, 4-dihydroxy-2-naphthoateoctaprenyltransferase	**2.13**
BCE_G9241_3159	hypothetical_protein_protein	**2.16**
BCE_G9241_3162	*gloA*; lactoylglutathione_lyase	**2.18**
BCE_G9241_3182	hypothetical_protein_protein	**2.97**
BCE_G9241_3207	hypothetical_protein_membrane_Spanning_protein	**3.48**
BCE_G9241_3214	conserved_hypothetical_protein_protein	**2.29**
BCE_G9241_3215	conserved_protein	**4.26**
BCE_G9241_3235	major_facilitator_family_transporter_putative	**2.18**
BCE_G9241_3242	conserved_hypothetical_protein_protein	**2.97**
BCE_G9241_3243	SMR_drug_efflux_transporter	**4.08**
BCE_G9241_3244	transcriptional_regulator_tetR_family_domain_protein	**4.65**
BCE_G9241_3245	perfringolysin_O_precursor	**110.36**
BCE_G9241_3259	conserved_hypothetical_protein_integral_membrane_protein_putative	**2.04**
BCE_G9241_3260	extracellular_ribonuclease	**12.99**
BCE_G9241_3291	haloacid_dehalogenase-like_hydrolase_putative	**2.22**
BCE_G9241_3342	serine_transporter_SdaC	**2.71**
BCE_G9241_3353	conserved_hypothetical_protein_protein	**2.09**
BCE_G9241_3354	phenazine_biosynthesis_protein_PhzF_family_putative	**2.68**
BCE_G9241_3355	acetyltransferase	**3.10**
BCE_G9241_3356	BtrF_putative	**4.08**
BCE_G9241_3357	acetyltransferase_GNAT_family	**2.59**
BCE_G9241_3361	transcriptional_regulator_MarR_family	**2.01**
BCE_G9241_3368	hypothetical_protein_membrane_Associated_protein	**2.63**
BCE_G9241_3393	hypothetical_protein_membrane_Spanning_protein	**2.06**
BCE_G9241_3404	LMBE-related_protein	**2.29**
BCE_G9241_3405	conserved_hypothetical_protein_protein	**2.61**
BCE_G9241_3406	*arsB*; transporter_NadC/P/Pho87_family	**2.32**
BCE_G9241_3424	phosphoglycerate_mutase_family_protein	**2.36**
BCE_G9241_3428	alpha-amylase	**2.13**
BCE_G9241_3442	glycerophosphoryl_diester_phosphodiesterase	**6.15**
BCE_G9241_3453	FAD-linked_oxidases_C-terminal_domain_protein	**2.81**
BCE_G9241_3468	hypothetical_protein_protein	**43.50**
BCE_G9241_3498	*panF*; pantothenate_permease	**3.22**
BCE_G9241_3517	molybdopterin_cofactor_biosynthesis_protein_A	**2.17**
BCE_G9241_3518	*fdhD*; formate_dehydrogenase_family_accessory_protein_FdhD	**2.05**
BCE_G9241_3519	hypothetical_protein_cytosolic_protein	**3.85**
BCE_G9241_3548	oxidoreductase_Gfo/Idh/MocA_family_VCA0099	**2.27**
BCE_G9241_3631	transcriptional_regulator_LysR_family	**2.75**

Table S6. Genes with increased expression in *B. cereus* G9241 in MGM in O_2		
**SEQUENCE ID**	**GENE INFO**	**Fold difference**
BCE_G9241_3645	ABC_transporter_ATP-binding/permease_protein_MDR_family	**2.09**
BCE_G9241_3666	*hspR*; regulatory_protein_GlnR	**2.13**
BCE_G9241_3678	*miaA*; tRNA_delta(2)-isopentenylpyrophosphate_transferase	**4.28**
BCE_G9241_3679	BRCA1	**2.68**
BCE_G9241_3680	extracellular_protein_putative	**3.29**
BCE_G9241_3681	*fruA*; PTS_system_fructose-specific_family_IIABC_components	**33.25**
BCE_G9241_3682	*fruB*; 1-phosphofructokinase	**18.73**
BCE_G9241_3683	*fruR*; transcriptional_regulator_DeoR_family	**13.14**
BCE_G9241_3684	alcohol_dehydrogenase_iron-containing	**2.59**
BCE_G9241_3705	*xth*; exodeoxyribonuclease_III	**2.21**
BCE_G9241_3712	phosphoglycerate_mutase_putative	**2.57**
BCE_G9241_3713	acetoin_dehydrogenase_E2_component_probable_putative	**4.57**
BCE_G9241_3732	phosphatidylinositol-specific_phospholipase_C_X_domain_protein	**31.51**
BCE_G9241_3733	microbial_collagenase	**16.06**
BCE_G9241_3765	oxidoreductase_short_chain_dehydrogenase/reductase_family_superf amily	**3.32**
BCE_G9241_3782	hypothetical_protein_protein	**2.03**
BCE_G9241_3784	hypothetical_protein_protein	**69.98**
BCE_G9241_3785	hypothetical_protein_protein	**6.98**
BCE_G9241_3786	prophage_LambdaW1_site-specific_recombinase_resolvase_family_putative	**3.70**
BCE_G9241_3840	DAK2_domain_protein	**2.10**
BCE_G9241_3841	putative_alkaline-shock_protein	**2.47**
BCE_G9241_3860	conserved_hypothetical_protein_protein	**2.27**
BCE_G9241_3907	*panE*; 2-dehydropantoate_2-reductase	**3.81**
BCE_G9241_3918	methyltransferase_putative	**3.87**
BCE_G9241_3919	conserved_hypothetical_protein_protein	**2.31**
BCE_G9241_3945	*typA*; GTP-binding_protein_typA	**2.00**
BCE_G9241_3946	conserved_hypothetical_protein_protein	**2.66**
BCE_G9241_3947	inositol_monophosphatase_family_protein	**3.24**
BCE_G9241_3951	lysine_decarboxylase	**2.34**
BCE_G9241_3966	putative_transcriptional_regulator	**4.69**
BCE_G9241_3967	metallo-beta-lactamase_superfamily_protein	**2.06**
BCE_G9241_3969	cytochrome_d_ubiquinol_oxidase_subunit_II_putative	**2.03**
BCE_G9241_3971	*dapD*; 2,3,4,5-tetrahydropyridine-2-carboxylate_N-_succinyltransferase	**2.84**
BCE_G9241_4006	*malF*; MW0191	**5.01**
BCE_G9241_4007	*malE*; maltose_ABC_transporter_periplasmic_maltose-_binding_protein	**4.72**
BCE_G9241_4010	ABC_transporter_ATP-binding/TOBE_domain_protein	**3.08**
BCE_G9241_4046	*ptsG*; PTS_system_glucose-specific_IIABC_component	**8.34**
BCE_G9241_4087	hypothetical_protein_protein	**2.82**
BCE_G9241_4088	xanthine/uracil_permease_family_protein	**2.96**
BCE_G9241_4089	*corA*; magnesium_and_cobalt_transport_protein_CorA	**2.15**
BCE_G9241_4090	*pdp*; pyrimidine-nucleoside_phosphorylase	**2.72**

**Table S6. Genes with increased expression in *B. cereus* G9241 in MGM in O_2**		
**SEQUENCE ID**	**GENE INFO**	**Fold difference**
BCE_G9241_4092	*deoB*; phosphopentomutase	**2.06**
BCE_G9241_4103	glutamate-rich_protein_grpB	**4.27**
BCE_G9241_4104	conserved_hypothetical_protein_protein	**2.29**
BCE_G9241_4131	*cpdB*; 2'3'-cyclic-nucleotide_2'-phosphodiesterase	**4.75**
BCE_G9241_4147	*sdhB*; L-serine_dehydratase_iron-sulfur-dependent_beta_subunit	**3.49**
BCE_G9241_4151	conserved_hypothetical_protein_protein	**2.08**
BCE_G9241_4165	hypothetical_protein_protein	**2.00**
BCE_G9241_4201	*ispA*; geranyltranstransferase	**2.23**
BCE_G9241_4205	*nusB*; transcription_antitermination_factor_nusB	**2.06**
BCE_G9241_4222	*aroQ*; 3-dehydroquinate_dehydratase_type_II	**2.25**
BCE_G9241_4234	lipoate-protein_ligase_A	**3.93**
BCE_G9241_4239	*sugE*; SugE_protein	**10.06**
BCE_G9241_4243	ABC_transporter_permease_protein	**2.21**
BCE_G9241_4244	ABC_transporter_permease_protein	**2.20**
BCE_G9241_4247	transcriptional_regulator_GntR_family	**2.23**
BCE_G9241_4263	conserved_hypothetical_protein_protein	**3.88**
BCE_G9241_4264	*aroK*; shikimate_kinase	**3.43**
BCE_G9241_4265	prolyl_4-hydroxylase_alpha_subunit	**7.24**
BCE_G9241_4294	hydroxyacylglutathione_hydrolase	**2.96**
BCE_G9241_4323	hypothetical_protein_cytosolic_protein	**2.93**
BCE_G9241_4333	*recO*; DNA_repair_protein_RecO	**2.01**
BCE_G9241_4347	conserved_hypothetical_protein_protein_TIGR00046	**2.92**
BCE_G9241_4353	*hrcA*; heat-inducible_transcription_repressor_HrcA	**4.84**
BCE_G9241_4355	transcriptional_regulator_MarR_family	**2.15**
BCE_G9241_4363	*comEB*; comE_operon_protein_2	**2.07**
BCE_G9241_4375	*psd*; phosphatidylserine_decarboxylase	**2.26**
BCE_G9241_4378	hypothetical_protein_cytosolic_protein	**2.21**
BCE_G9241_4379	*rpmG*; ribosomal_protein_L33	**3.50**
BCE_G9241_4414	conserved_hypothetical_protein_protein	**2.87**
BCE_G9241_4449	TPR_domain_protein	**2.97**
BCE_G9241_4477	*ruvA*; Holliday_junction_DNA_helicase_RuvA	**2.03**
BCE_G9241_4493	ABC_transporter_permease_protein_putative	**3.02**
BCE_G9241_4494	ABC_transporter_ATP-binding_protein	**3.62**
BCE_G9241_4495	sensor_histidine_kinase_putative	**2.67**
BCE_G9241_4496	DNA-binding_response_regulator	**2.27**
BCE_G9241_4502	*cafA*; cytoplasmic_axial_filament_protein	**2.50**
BCE_G9241_4513	*folC*; FolC_family_protein	**2.44**
BCE_G9241_4523	transcriptional_regulator_MarR_family	**4.14**
BCE_G9241_4530	TPR_repeat_protein	**3.05**
BCE_G9241_4687	DHH_subfamily_1_protein	**3.13**
BCE_G9241_4699	conserved_hypothetical_protein_protein	**5.07**
BCE_G9241_4700	sigma-70_region_2_family	**2.64**
BCE_G9241_4702	zwittermicin_A_resistance_protein_ZmaR	**4.33**
BCE_G9241_4703	conserved_hypothetical_protein_protein	**7.20**

Table S6. Genes with increased expression in *B. cereus* G9241 in MGM in O_2		
**SEQUENCE ID**	**GENE INFO**	**Fold difference**
BCE_G9241_4712	*argH*; argininosuccinate_lyase	**3.16**
BCE_G9241_4713	*argG*; argininosuccinate_synthase	**9.10**
BCE_G9241_4718	protein_ecsC	**2.46**
BCE_G9241_4727	metal-dependent_hydrolase	**2.13**
BCE_G9241_4732	aminotransferase_class_V	**2.37**
BCE_G9241_4738	*megL*; methionine_gamma-lyase	**3.73**
BCE_G9241_4743	*tyrS*; tyrosyl-tRNA_synthetase	**2.43**
BCE_G9241_4748	conserved_hypothetical_protein_protein	**3.61**
BCE_G9241_4749	acetyltransferase_CysE/LacA/LpxA/NodL_family	**6.59**
BCE_G9241_4761	oxidoreductase_Gfo/Idh/MocA_family	**2.83**
BCE_G9241_4762	*mscL*; large_conductance_mechanosensitive_channel_protein	**3.33**
BCE_G9241_4771	aminopeptidase_putative	**2.63**
BCE_G9241_4775	acetyltransferase_GNAT_family	**2.20**
BCE_G9241_4778	transcriptional_regulator_PadR_family	**2.29**
BCE_G9241_4785	conserved_hypothetical_protein_protein	**2.35**
BCE_G9241_4786	*pheT*; tRNA_binding_domain_protein	**2.32**
BCE_G9241_4788	thioredoxin	**4.00**
BCE_G9241_4790	glutamyl-aminopeptidase	**2.33**
BCE_G9241_4803	conserved_hypothetical_protein_protein_TIGR00275	**2.56**
BCE_G9241_4804	drug_resistance_transporter_EmrB/QacA_family_protein	**5.89**
BCE_G9241_4806	Aha1_domain_superfamily	**3.11**
BCE_G9241_4834	*leuS*; leucyl-tRNA_synthetase	**2.49**
BCE_G9241_4872	conserved_hypothetical_protein_protein	**9.80**
BCE_G9241_4873	cell_wall_surface_anchor_family_protein	**2.56**
BCE_G9241_4903	S-layer_homology_domain	**9.51**
BCE_G9241_4906	YitT-like_protein	**2.45**
BCE_G9241_4917	membrane_protein_putative	**2.16**
BCE_G9241_4924	conserved_hypothetical_protein_protein	**2.38**
BCE_G9241_4938	hypothetical_protein_membrane_Spanning_protein	**3.07**
BCE_G9241_4939	transcription_regulator_arsR_family	**3.23**
BCE_G9241_4940	DNA-binding_response_regulator	**2.63**
BCE_G9241_4954	dihydroorotase	**2.03**
BCE_G9241_4962	*menE*; O-succinylbenzoate-CoA_ligase	**4.16**
BCE_G9241_4966	*menF*; isochorismate_synthase	**3.70**
BCE_G9241_4967	*menA*; 1,4-dihydroxy-2-naphthoateoctaprenyltransferase	**3.18**
BCE_G9241_4993	MW0837	**2.16**
BCE_G9241_4994	*aspB-3*; aspartate_aminotransferase	**2.06**
BCE_G9241_4995	*lrp*; leucine_responsive_regulatory_protein	**2.69**
BCE_G9241_4999	aminotransferase_class_II	**3.77**
BCE_G9241_5000	hypothetical_protein_protein	**2.05**
BCE_G9241_5005	*arsB*; transporter_NadC/P/Pho87_family	**2.02**
BCE_G9241_5014	*manB*; phosphoglucomutase/phosphomannomutase_family_protein	**2.26**
BCE_G9241_5016	*pepA*; cytosol_aminopeptidase	**2.08**
BCE_G9241_5020	*ndh*; NADH_dehydrogenase	**6.03**

Table S6. Genes with increased expression in *B. cereus* G9241 in MGM in O_2		
**SEQUENCE ID**	**GENE INFO**	**Fold difference**
BCE_G9241_5025	hypothetical_protein_cytosolic_protein	**2.28**
BCE_G9241_5033	pyridine_nucleotide-disulphide_oxidoreductase_family_protein_putative	**3.77**
BCE_G9241_5036	*phnA*; alkylphosphonate_utilization_operon_protein_PhnA	**2.42**
BCE_G9241_5041	YuzD-like_protein	**2.17**
BCE_G9241_5043	conserved_hypothetical_protein_protein	**2.35**
BCE_G9241_5044	CotS-related_protein	**2.47**
BCE_G9241_5053	hypothetical_protein_protein	**40.85**
BCE_G9241_5064	hypothetical_protein_membrane_Spanning_protein	**2.18**
BCE_G9241_5079	*sufC*; FeS_assembly_ATPase_sufC	**3.77**
BCE_G9241_5089	arsenate_reductase_putative	**2.70**
BCE_G9241_5090	conserved_hypothetical_protein_protein	**20.37**
BCE_G9241_5112	conserved_hypothetical_protein_protein_TIGR00106	**2.04**
BCE_G9241_5113	methyl-accepting_chemotaxis_transducer_putative	**5.64**
BCE_G9241_5119	amino_acid_permease	**2.19**
BCE_G9241_5123	pheromone_cCF10_percursor/lipoprotein_60_kDa	**2.34**
BCE_G9241_5124	conserved_hypothetical_protein_protein	**2.95**
BCE_G9241_5125	YitT_family_protein_putative	**4.60**
BCE_G9241_5130	conserved_hypothetical_protein_protein	**3.49**
BCE_G9241_5131	transcriptional_regulator_Cro/CI_family	**5.38**
BCE_G9241_5132	hypothetical_protein_protein	**2.38**
BCE_G9241_5134	conserved_hypothetical_protein_protein	**35.82**
BCE_G9241_5135	protein_erfK/srfK_precursor	**7.90**
BCE_G9241_5136	*phoB*; two-component_response_regulator_vanRB	**5.35**
BCE_G9241_5137	sensor_histidine_kinase_VncS_putative	**2.90**
BCE_G9241_5145	*drrA*; response_regulator_DrrA	**4.69**
BCE_G9241_5169	pyrimidine_nucleoside_trans	**57.48**
BCE_G9241_5171	sodium/alanine_symporter_VC2356	**2.18**
BCE_G9241_5178	major_facilitator_family_transporter	**3.14**
BCE_G9241_5184	SET_domain_putative	**2.24**
BCE_G9241_5185	methyl-accepting_chemotaxis_protein_putative	**3.43**
BCE_G9241_5186	endonuclease/exonuclease/phosphatase_family_protein_putative	**6.57**
BCE_G9241_5205	*smpB*; SsrA-binding_protein	**2.36**
BCE_G9241_5208	*est*; carboxylesterase	**2.53**
BCE_G9241_5209	*secG*; preprotein_translocase_secG_subunit	**3.45**
BCE_G9241_5211	murein_hydrolase_exporter	**2.96**
BCE_G9241_5241	hypothetical_protein_protein	**4.65**
BCE_G9241_5258	transcriptional_regulator_LysR_family_putative	**2.26**
BCE_G9241_5264	hypothetical_protein_membrane_Spanning_protein	**2.40**
BCE_G9241_5275	cold_shock_protein-related_protein	**4.65**
BCE_G9241_5281	DegV_family_protein	**2.57**
BCE_G9241_5282	conserved_hypothetical_protein_protein_TIGR00257	**3.54**
BCE_G9241_5283	cell-envelope_associated_acid_phosphatase	**2.56**
BCE_G9241_5292	hypothetical_protein_protein	**4.32**

Table S6. Genes with increased expression in *B. cereus* G9241 in MGM in O_2		
**SEQUENCE ID**	**GENE INFO**	**Fold difference**
BCE_G9241_5344	YdjC-like_protein	**5.67**
BCE_G9241_5345	6-phospho-beta-glucosidase	**29.65**
BCE_G9241_5346	PTS_system_IIA_component	**8.81**
BCE_G9241_5347	*celB*; PTS_system_cellobiose-specific_IIC_component	**5.37**
BCE_G9241_5348	*celA*; PTS_system_IIB_component	**16.48**
BCE_G9241_5359	hypothetical_protein	**3.03**
BCE_G9241_5387	L-lactate_transporter	**3.55**
BCE_G9241_5398	nupC_family_protein	**7.33**
BCE_G9241_5399	diguanylate_cyclase/phosphodiesterase_domain_1	**4.24**
BCE_G9241_5400	glycosyl_transferase_group_2_family_protein_putative	**2.19**
BCE_G9241_5404	conserved_hypothetical_protein_protein	**2.44**
BCE_G9241_5407	membrane_protein_with_C2C2_zinc_finger	**3.00**
BCE_G9241_5408	conserved_hypothetical_protein_protein	**3.43**
BCE_G9241_5409	hypothetical_protein_membrane_Associated_protein	**3.99**
BCE_G9241_5417	hypothetical_protein_membrane_Spanning_protein	**2.46**
BCE_G9241_5418	conserved_hypothetical_protein_integral_membrane_protein	**4.22**
BCE_G9241_5419	ABC_transporter_ATP-binding_protein	**2.65**
BCE_G9241_5422	transposase_IS605-TnpB_family_putative	**2.21**
BCE_G9241_5458	ABC_transporter_ATP-binding_protein_putative	**3.71**
BCE_G9241_5478	integral_membrane_protein	**2.73**
BCE_G9241_5493	3-demethylubiquinone-9_3-methyltransferase/sugar-_phosphate_isomerase_family_protein	**2.83**
BCE_G9241_5494	protein_tyrosine_phosphatase	**2.25**
BCE_G9241_5495	phosphotransferase_system_enzyme_IIA-like_protein	**2.88**
BCE_G9241_5498	conserved_hypothetical_protein_protein	**2.25**
BCE_G9241_5499	membrane_protein_putative	**2.18**
BCE_G9241_5500	Sua5/YciO/YrdC/YwlC_family_protein	**3.12**
BCE_G9241_5504	*prfA*; peptide_chain_release_factor_1	**2.32**
BCE_G9241_5524	PapR-related_protein	**13.97**
BCE_G9241_5525	transcription_regulator_PlcR_probable_putative	**2.48**
BCE_G9241_5532	conserved_hypothetical_protein_protein	**8.25**
BCE_G9241_5537	conserved_hypothetical_protein_protein	**3.83**
BCE_G9241_5538	hypothetical_protein_protein	**3.50**
BCE_G9241_5539	microcin_immunity_protein_MccF_VCA0439	**2.55**
BCE_G9241_5546	*speE*; spermidine_synthase	**3.45**
BCE_G9241_5549	glycerophosphoryl_diester_phosphodiesterase_putative	**2.77**
BCE_G9241_5566	lipoate-protein_ligase_A_family_protein	**3.52**
BCE_G9241_5567	*pta*; phosphate_acetyltransferase	**3.58**
BCE_G9241_5578	xanthine_permease_putative	**2.03**
BCE_G9241_5583	bacitracin_transport_permease_protein_BCRB	**2.44**
BCE_G9241_5584	bacitracin_transport_ATP-binding_protein_bcrA	**2.63**
BCE_G9241_5585	lipase/acylhydrolase_putative	**5.14**
BCE_G9241_5586	sugar-binding_transcriptional_regulator_LacI_family_putative	**2.24**
BCE_G9241_5600	GGDEF_family_protein	**2.05**

Table S6. Genes with increased expression in *B. cereus* G9241 in MGM in O_2		
**SEQUENCE ID**	**GENE INFO**	**Fold difference**
BCE_G9241_5601	hypothetical_protein_cytosolic_protein-related_protein	**2.12**
BCE_G9241_5602	*cstA*; carbon_starvation_protein_CstA	**3.88**
BCE_G9241_5603	response_regulator_putative	**6.74**
BCE_G9241_5610	hypothetical_protein_membrane_Spanning_protein	**3.12**
BCE_G9241_5617	ABC_transporter_ATP-binding_protein_uup	**3.80**
BCE_G9241_5626	LrgB_family_protein	**19.80**
BCE_G9241_5627	hypothetical_protein_protein	**26.63**
BCE_G9241_5628	LrgA_family_protein	**43.01**
BCE_G9241_5630	*lytS*; sensor_histidine_kinase	**3.38**
BCE_G9241_5636	conserved_hypothetical_protein_protein	**4.10**
BCE_G9241_5643	*deaD*; ATP-dependent_RNA_helicase_DEAD/DEAH_box_family	**2.85**
BCE_G9241_5644	membrane_protein_putative	**3.83**
BCE_G9241_5657	*drrA*; response_regulator	**2.17**
BCE_G9241_5666	*ychF*; GTP-binding_protein_YchF	**2.52**
BCE_G9241_5668	mechanosensitive_ion_channel	**2.43**
BCE_G9241_CNI_0003	conserved_hypothetical_protein_protein	**3.07**
BCE_G9241_CNI_0066	hypothetical_protein_protein	**4.27**
BCE_G9241_CNI_0081	hypothetical_protein	**2.10**
BCE_G9241_CNI_0249	*metS*; methionyl-tRNA_synthetase	**5.11**
BCE_G9241_CNI_0258	*map*; methionine_aminopeptidase_type_I	**2.45**
BCE_G9241_CNI_0272	sensor_protein_vanSB	**4.21**
BCE_G9241_CNI_0273	proteinase_VCA0223	**31.63**
BCE_G9241_CNI_0288	hypothetical_protein_protein	**2.74**
BCE_G9241_CNI_0290	hypothetical_protein_protein	**69.42**
BCE_G9241_CNI_0291	glycerol_uptake_facilitator_protein	**21.31**
BCE_G9241_CNI_0292	*glpK*; glycerol_kinase	**11.05**
BCE_G9241_CNI_0293	DNA-binding_response_regulator	**5.55**
BCE_G9241_CNI_0301	bacitracin_transport_permease_protein_BCRB	**2.18**
BCE_G9241_CNI_0304	flagellar_biosynthetic_protein_fliR_putative	**8.58**
BCE_G9241_CNI_0305	drug_resistance_transporter_EmrB/QacA_subfamily	**2.22**
BCE_G9241_CNI_0316	conserved_hypothetical_protein_protein	**3.81**
BCE_G9241_pBClin29_0017	hypothetical_protein_protein	**2.66**
BCE_G9241_pBClin29_0020	conserved_hypothetical_protein_protein	**3.56**
BCE_G9241_pBCXO1_0019	conserved_hypothetical_protein_protein	**3.88**
BCE_G9241_pBCXO1_0053	conserved_hypothetical_protein_protein	**2.68**
