# Supplementary material for: Comparative Transcriptional Profiling of Bacillus cereus Sensu Lato Strains during Growth in CO2-Bicarbonate and Aerobic Atmospheres
Source: PLoS One. 2009 Mar 19;4(3):e4904. doi: 10.1371/journal.pone.0004904 (PMC2654142; doi:10.1371/journal.pone.0004904)
Supplement: Table S7 — Genes with increased expression in B. anthracis Sterne (34F2) in CO2 (MGM+0.8% bicarbonate) (0.17 MB PDF) [file pone.0004904.s007.pdf]

Table S7. Genes with increased expression in *B. anthracis* Sterne ($34F_2$) in CO_2 (MGM + 0.8% bicarbonate)		
***SEQUENCE ID**	**GENE INFO**	**Fold difference**
GBAA0167	hypothetical protein	**10.17**
GBAA0231	oligopeptide abc transporter, oligopeptide-binding protein, putative	**6.89**
GBAA0232	oligopeptide abc transporter, permease protein	**5.91**
GBAA0360	hypothetical protein	**2.80**
GBAA0366	fatty acid desaturase	**2.52**
GBAA0368	amino acid abc transporter, atp-binding protein	**3.84**
GBAA0405	cation-transporting atpase, e1-e2 family	**8.06**
GBAA0543	penicillin-binding domain protein	**2.10**
GBAA0549	hypothetical protein	**4.05**
GBAA0566	glycerol-3-phosphate abc transporter, atp-binding protein, putative	**2.35**
GBAA0567	glycerol-3-phosphate abc transporter, permease protein, putative	**2.83**
GBAA0569	glycerol-3-phosphate abc transporter, glycerol-3-phosphate-binding protein, putative	**2.13**
GBAA0650	sensor histidine kinase	**4.59**
GBAA0651	dna-binding response regulator	**3.61**
GBAA0715	phosphate abc transporter, phosphate-binding protein, putative	**16.41**
GBAA0716	phosphate abc transporter, permease protein, putative	**9.92**
GBAA0717	phosphate abc transporter, permease protein, putative	**9.96**
GBAA0747	xanthine/uracil permease family protein	**2.18**
GBAA0760	lipoprotein, putative	**2.93**
GBAA0785	na/pi-cotransporter family protein	**5.32**
GBAA0789	hypothetical protein	**3.37**
GBAA0790	transcription antiterminator, bglg family	**2.29**
GBAA0797	abc transporter, permease protein, putative	**2.34**
GBAA0849	*gapN*; glyceraldehyde-3-phosphate dehydrogenase, nadp-dependent	**2.49**
GBAA0855	amino acid abc transporter, amino acid-binding protein	**2.04**
GBAA0856	amino acid abc transporter, permease protein	**2.15**
GBAA0860	hypothetical protein	**2.23**
GBAA0872	n-acetylmuramoyl-l-alanine amidase, family 2	**2.28**
GBAA0873	hypothetical protein	**2.43**
GBAA0878	sulfate permease family protein	**2.62**
GBAA0887	*eag*: s-layer protein ea1	**21.25**
GBAA0902	ornithine cyclodeaminase	**2.32**
GBAA0906	hypothetical protein	**2.28**
GBAA0916	hypothetical protein	**2.94**
GBAA0918	hypothetical protein	**2.99**
GBAA0919	hypothetical protein	**2.58**
GBAA0936	lipoprotein, putative	**3.06**
GBAA0939	hypothetical protein	**2.38**
GBAA0956	hypothetical protein	**2.49**
GBAA1005	hypothetical protein	**6.70**
GBAA1006	hypothetical protein	**4.13**
GBAA1007	hypothetical protein	**4.06**
GBAA1038	drug resistance transporter, emrb/qaca family	**3.11**

Table S7. Genes with increased expression in *B. anthracis* Sterne ($34F_2$) in CO_2 (MGM + 0.8% bicarbonate)		
***SEQUENCE ID**	**GENE INFO**	**Fold difference**
GBAA1039	hypothetical protein	**4.53**
GBAA1091	acyl-CoA synthase	**7.24**
GBAA1130	s-layer protein, putative	**6.54**
GBAA1132	*aceA*; isocitrate lyase	**3.35**
GBAA1142	*addA*; atp-dependent nuclease, subunit a	**2.11**
GBAA1143	hypothetical protein	**2.16**
GBAA1144	*gerPF-1*; spore germination protein gerpf	**2.19**
GBAA1153	hypothetical protein	**2.52**
GBAA1185	*fabF*; 3-oxoacyl-(acyl carrier protein) synthase	**2.03**
GBAA1274	hypothetical protein	**2.34**
GBAA1308	glycolate oxidase, iron-sulfur subunit, putative	**9.21**
GBAA1309	*glcD*; glycolate oxidase, subunit glcd	**9.72**
GBAA1321	formate/nitrite transporter family protein	**6.91**
GBAA1337	iron compound abc transporter, iron compound-binding protein, putative	**2.99**
GBAA1354	hypothetical protein	**6.18**
GBAA1363	hypothetical protein	**2.69**
GBAA1417	*ilvB-1*; acetolactate synthase large subunit	**2.69**
GBAA1418	*ilvN*; acetolactate synthase III small subunit	**3.18**
GBAA1419	*ilvC-1*; ketol-acid reductoisomerase	**3.42**
GBAA1420	*leuA;* 2-isopropylmalate synthase	**3.90**
GBAA1421	*leuB*; 3-isopropylmalate dehydrogenase	**3.81**
GBAA1422	*leuC*; isopropylmalate isomerase large subunit	**3.33**
GBAA1423	*leuD*; isopropylmalate isomerase small subunit	**2.92**
GBAA1430	*hisF*; imidazole glycerol phosphate synthase subunit HisF	**2.02**
GBAA1432	histidinol-phosphatase	**3.05**
GBAA1433	hypothetical protein	**2.88**
GBAA1470	hypothetical protein	**3.98**
GBAA1526	*gpsA*; NAD(P)H-dependent glycerol-3-phosphate dehydrogenase	**3.05**
GBAA1543	hypothetical protein	**2.18**
GBAA1546	*qcrC*; menaquinol-cytochrome c reductase, cytochrome b/c subunit	**2.50**
GBAA1562	*panB*; 3-methyl-2-oxobutanoate hydroxymethyltransferase	**3.49**
GBAA1563	*panC*; pantoate--beta-alanine ligase	**4.52**
GBAA1564	*panD*; aspartate 1-decarboxylase precursor	**3.68**
GBAA1577	hypothetical protein	**2.04**
GBAA1605	cation transporter, putative	**3.01**
GBAA1634	hypothetical protein	**5.55**
GBAA1635	sodium/solute symporter family protein	**5.23**
GBAA1636	adenosylmethionine--8-amino-7-oxononanoate transaminase	**2.98**
GBAA1639	germination protein gern	**11.70**
GBAA1734	abc transporter, atp-binding protein	**4.01**
GBAA1735	abc transporter, substrate-binding protein, putative	**2.60**
GBAA1736	abc transporter, permease protein, putative	**2.44**
GBAA1737	metallo-beta-lactamase family protein	**2.95**
GBAA1738	hypothetical protein	**2.41**

Table S7. Genes with increased expression in *B. anthracis* Sterne ($34F_2$) in CO_2 (MGM + 0.8% bicarbonate)		
***SEQUENCE ID**	**GENE INFO**	**Fold difference**
GBAA1759	transporter, eama family	**2.21**
GBAA1826	3-oxoacyl-(acyl carrier protein) synthase	**3.03**
GBAA1834	polysaccharide biosynthesis family protein	**2.25**
GBAA1899	hypothetical protein	**2.35**
GBAA1900	bnr repeat domain protein	**7.66**
GBAA1944	*cydB-1*; cytochrome d ubiquinol oxidase, subunit ii	**36.62**
GBAA1945	transport atp-binding protein cydc	**65.50**
GBAA1946	transport atp-binding protein cydd	**24.51**
GBAA1947	hypothetical protein	**7.14**
GBAA1951	hypothetical protein	**3.04**
GBAA1953	hydrolase, alpha/beta fold family	**3.64**
GBAA1973	transcription antiterminator, lytr family	**2.70**
GBAA1976	sensor histidine kinase	**2.31**
GBAA2013	*dpS*; general stress protein	**2.65**
GBAA2084	hypothetical protein	**2.78**
GBAA2126	*narH*; respiratory nitrate reductase, beta subunit	**2.16**
GBAA2140	hypothetical protein	**2.66**
GBAA2142	precorrin-2 dehydrogenase	**3.63**
GBAA2143	cbix domain protein	**3.65**
GBAA2235	hypothetical protein	**4.75**
GBAA2244	hypothetical protein	**3.76**
GBAA2245	hypothetical protein	**2.52**
GBAA2249	sco1/senc family lipoprotein	**2.97**
GBAA2267	alcohol dehydrogenase	**7.62**
GBAA2300	*kamA*; l-lysine 2,3-aminomutase	**23.71**
GBAA2301	hypothetical protein	**34.72**
GBAA2306	hypothetical protein	**4.16**
GBAA2307	protein kinase domain protein	**2.82**
GBAA2308	sporulation-control protein spo0m, putative	**4.49**
GBAA2348	*mmgD*; citrate synthase	**3.22**
GBAA2353	*garR*; 2-hydroxy-3-oxopropionate reductase	**2.15**
GBAA2354	*mmsA-1*; methylmalonic acid semialdehyde dehydrogenase	**2.21**
GBAA2457	o-methyltransferase family protein	**4.12**
GBAA2458	hypothetical protein	**3.46**
GBAA2459	hypothetical protein	**2.80**
GBAA2500	hypothetical protein	**2.34**
GBAA2531	abc transporter, atp-binding protein	**2.07**
GBAA2534	acetyltransferase, gnat family	**9.53**
GBAA2535	hypothetical protein	**7.23**
GBAA2536	spore coat protein, putative	**6.62**
GBAA2537	hypothetical protein	**7.96**
GBAA2538	metallo-beta-lactamase/rhodanese-like domain protein	**8.77**
GBAA2539	hypothetical protein	**2.11**
GBAA2547	acyl-coa dehydrogenase	**2.65**

Table S7. Genes with increased expression in *B. anthracis* Sterne ($34F_2$) in CO_2 (MGM + 0.8% bicarbonate)		
***SEQUENCE ID**	**GENE INFO**	**Fold difference**
GBAA2548	acetyl-CoA carboxylase	**3.06**
GBAA2550	*mvaB;* hydroxymethylglutaryl-CoA lyase	**5.67**
GBAA2551	enoyl-CoA hydratase	**3.88**
GBAA2552	carboxyl transferase domain protein	**6.68**
GBAA2553	acetoacetyl-coa synthase, putative	**6.73**
GBAA2574	hypothetical protein	**4.57**
GBAA2575	penicillin-binding protein, putative	**3.96**
GBAA2576	transcriptional regulator, merr family	**2.42**
GBAA2578	mutt/nudix family protein	**2.00**
GBAA2632	cytochrome p450 family protein	**3.08**
GBAA2692	hypothetical protein	**3.25**
GBAA2693	hypothetical protein	**7.60**
GBAA2695	hypothetical protein	**9.14**
GBAA2761	acetoin operon transcriptional activator, putative	**3.99**
GBAA2782	cpsh domain protein	**2.83**
GBAA2783	hypothetical protein	**2.80**
GBAA2839	hypothetical protein	**9.82**
GBAA2840	hypothetical protein	**10.30**
GBAA2841	hypothetical protein	**4.57**
GBAA3118	metallo-beta-lactamase family protein	**3.81**
GBAA3239	hypothetical protein	**2.04**
GBAA3284	tpr domain protein	**2.11**
GBAA3357	hypothetical protein	**2.04**
GBAA3358	nad(p)h dehydrogenase, quinone family	**2.20**
GBAA3359	hypothetical protein	**2.28**
GBAA3360	hypothetical protein	**2.08**
GBAA3420	hypothetical protein	**3.48**
GBAA3501	lysozyme, putative	**2.02**
GBAA3502	hypothetical protein	**2.73**
GBAA3503	hypothetical protein	**3.60**
GBAA3504	hypothetical protein	**2.95**
GBAA3506	penicillin-binding protein, putative	**3.43**
GBAA3508	hypothetical protein	**2.37**
GBAA3529	hypothetical protein	**2.58**
GBAA3609	*dhaS*; aldehyde dehydrogenase	**4.08**
GBAA3653	abc transporter, atp-binding protein	**2.40**
GBAA3663	anaerobic ribonucleoside triphosphate reductase	**11.83**
GBAA3833	*glnA*; glutamine synthetase, type i	**2.49**
GBAA3834	*glnR*; transcriptional repressor glnr	**3.83**
GBAA3859	heavy metal-transporting atpase	**6.55**
GBAA3860	copper-ion-binding protein	**10.70**
GBAA3895	sulfatase	**7.85**
GBAA3985	*ftsY*; signal recognition particle-docking protein ftsy	**2.23**
GBAA4050	*spoVE*; stage v sporulation protein e	**2.48**

Table S7. Genes with increased expression in *B. anthracis* Sterne ($34F_2$) in CO_2 (MGM + 0.8% bicarbonate)		
***SEQUENCE ID**	**GENE INFO**	**Fold difference**
GBAA4149	hydrolase, carbon-nitrogen family	**3.71**
GBAA4150	hypothetical protein	**3.04**
GBAA4151	*ctaF*; cytochrome c oxidase, subunit ivb	**2.87**
GBAA4152	*ctaE*; cytochrome c oxidase, subunit iii	**2.41**
GBAA4153	*ctaD*; cytochrome c oxidase, subunit i	**2.27**
GBAA4154	*ctaC*; cytochrome c oxidase, subunit ii	**2.03**
GBAA4156	*ctaA*; cytochrome aa3 controlling protein	**2.34**
GBAA4161	phoh family protein	**2.10**
GBAA4218	*metE*; 5-methyltetrahydropteroyltriglutamate-- homocysteine methyltransferase	**2.37**
GBAA4224	hypothetical protein	**17.83**
GBAA4238	hypothetical protein	**2.27**
GBAA4242	hypothetical protein	**4.39**
GBAA4379	hypothetical protein	**2.14**
GBAA4380	mutt/nudix family protein	**3.09**
GBAA4427	hydrolase, haloacid dehalogenase-like family	**2.45**
GBAA4469	sodium:dicarboxylate symporter family protein	**5.84**
GBAA4492	phosphate transport system regulatory protein phou, putative	**5.62**
GBAA4493	*pstB*; phosphate abc transporter, atp-binding protein	**13.04**
GBAA4494	*pstA*; phosphate abc transporter, permease protein	**18.28**
GBAA4495	*pstC*; phosphate abc transporter, permease protein	**18.35**
GBAA4496	*phoX*; phosphate abc transporter, phosphate-binding protein	**24.10**
GBAA4574	alkaline phosphatase	**5.36**
GBAA4576	acetyltransferase, gnat family	**2.03**
GBAA4586	phenylalanine-4-hydroxylase, putative	**2.07**
GBAA4625	*trmU*; trna (5-methylaminomethyl-2-thiouridylate)-methyltransferase	**2.07**
GBAA4626	aminotransferase, class v	**2.37**
GBAA4627	rrf2 family protein	**2.65**
GBAA4695	*hemD*; uroporphyrinogen-III synthetase	**2.12**
GBAA4697	hemx protein	**2.50**
GBAA4698	*hemA*; glutamyl-tRNA reductase	**3.06**
GBAA4799	hypothetical protein	**6.56**
GBAA4800	hypothetical protein	**9.44**
GBAA4807	hd domain protein	**3.10**
GBAA4875	universal stress protein family	**4.90**
GBAA4895	hypothetical protein	**2.06**
GBAA4896	acetyl-coa synthetase, putative	**2.98**
GBAA4917	*acuB*; acetoin utilization protein acub	**2.02**
GBAA4927	hypothetical protein	**4.51**
GBAA4928	hypothetical protein	**4.61**
GBAA4993	sodium/hydrogen exchanger family protein	**2.72**
GBAA4994	trka domain protein	**2.32**
GBAA4996	abc transporter, permease protein, putative	**3.38**
GBAA4997	abc transporter, atp-binding protein	**2.79**
GBAA5003	abc transporter, atp-binding protein, putative	**2.43**

Table S7. Genes with increased expression in *B. anthracis* Sterne ($34F_2$) in CO_2 (MGM + 0.8% bicarbonate)		
***SEQUENCE ID**	**GENE INFO**	**Fold difference**
GBAA5004	hypothetical protein	**2.06**
GBAA5046	hypothetical protein	**3.91**
GBAA5071	hypothetical protein	**2.90**
GBAA5077	hypothetical protein	**2.24**
GBAA5191	nad(p)h dehydrogenase, quinone family	**2.18**
GBAA5246	acyl-coa dehydrogenase	**2.44**
GBAA5247	hypothetical protein	**2.14**
GBAA5249	3-hydroxyacyl-coa dehydrogenase/enoyl-coa hydratase/isomerase family protein	**2.09**
GBAA5287	trka domain protein	**2.02**
GBAA5300	sodium/alanine symporter family protein	**3.56**
GBAA5360	phage major capsid protein, hk97 family	**2.51**
GBAA5370	*cggR*; gapa transcriptional regulator cggr	**5.46**
GBAA5439	chromate ion transporter	**3.79**
GBAA5532	*nuoN*; NADH dehydrogenase subunit N	**2.25**
GBAA5534	*nuoL*; NADH dehydrogenase subunit L	**2.19**
GBAA5535	*nuoK;* NADH dehydrogenase kappa subunit	**3.11**
GBAA5536	*nuoJ*; NADH dehydrogenase subunit J	**2.19**
GBAA5537	*nuoI*; NADH dehydrogenase subunit I	**2.18**
GBAA5606	aminopeptidase, putative	**2.51**
GBAA5634	hypothetical protein	**2.04**
GBAA5639	d-alanyl-d-alanine carboxypeptidase, putative	**2.66**
GBAA5655	*metA*; homoserine O-succinyltransferase	**2.01**
GBAA5656	O-acetylhomoserine sulfhydrylase	**2.26**
GBAApXO1_0023	hypothetical protein	**2.75**
GBAApXO1_0121	hypothetical protein	**3.53**
GBAApXO1_0123	hypothetical protein	**40.58**
GBAApXO1_0124	s-layer protein, (pxo1-90)	**35.13**
GBAApXO1_0125	phosphatase, pap2 family, (pxo1-91)	**12.03**
GBAApXO1_0137	hypothetical protein	**48.25**
GBAApXO1_0138	hypothetical protein	**2.71**
GBAApXO1_0139	hypothetical protein	**2.67**
GBAApXO1_0140	hypothetical protein	**2.28**
GBAApXO1_0142	calmodulin-sensitive adenylate cyclase	**2.08**
GBAApXO1_0146	transcriptional activator atxa, (pxo1-119)	**5.24**
GBAApXO1_0153	hypothetical protein	**9.53**
GBAApXO1_0154	transposase x	**2.44**
GBAApXO1_0156	spore germination protein xb	**2.26**
GBAApXO1_0157	spore germination protein xa	**2.32**
GBAApXO1_0158	spore germination protein xc, (pxo1-112)	**2.44**
GBAApXO1_0159	hypothetical protein	**3.08**
GBAApXO1_0160	hypothetical protein	**2.57**
GBAApXO1_0164	protective antigen	**11.80**
GBAApXO1_0165	hypothetical protein	**11.85**
GBAApXO1_0166	transcriptional repressor pagr, (pxo1-109)	**15.07**

**Table S7. Genes with increased expression in *B. anthracis* Sterne (34F_2) in CO_2 (MGM + 0.8% bicarbonate)**		
***SEQUENCE ID**	**GENE INFO**	**Fold difference**
GBAApXO1_0171	ribonuclease domain protein	**6.05**
GBAApXO1_0172	lethal factor	**5.34**

*Sequence ID numbers are GBAA locus tags from the *Bacillus anthracis* Ames Ancestor genome.
