# Supplementary material for: Comparative Transcriptional Profiling of Bacillus cereus Sensu Lato Strains during Growth in CO2-Bicarbonate and Aerobic Atmospheres
Source: PLoS One. 2009 Mar 19;4(3):e4904. doi: 10.1371/journal.pone.0004904 (PMC2654142; doi:10.1371/journal.pone.0004904)
Supplement: Table S8 — Genes with increased expression in B. anthracis Sterne (34F2) in MGM in O2 (0.16 MB PDF) [file pone.0004904.s008.pdf]

Table S8. Genes with increased expression in *B. anthracis* Sterne ($34F_2$) in MGM in O_2		
***SEQUENCE ID**	**GENE INFO**	**Fold difference**
GBAA0027	*tdk*; thymidylate kinase	**2.03**
GBAA0034	*abrB*; transition state transcriptional regulatory protein abrb	**4.76**
GBAA0065	transcriptional activator, putative, baf family	**2.24**
GBAA0066	*hslO*; Hsp33-like chaperonin	**2.19**
GBAA0068	*pabB*; para-aminobenzoate synthase component I	**2.28**
GBAA0069	*pabA-1*; para-aminobenzoate synthase component II	**2.70**
GBAA0070	*pabC*; 4-amino-4-deoxychorismate lyase	**2.16**
GBAA0077	*ctsR*; transcriptional regulator ctsr	**2.23**
GBAA0082	dna-binding protein, putative	**2.03**
GBAA0154	*rocF*; arginase	**2.54**
GBAA0158	hypothetical protein	**5.37**
GBAA0159	*glmS*; D-fructose-6-phosphate amidotransferase	**3.08**
GBAA0168	hypothetical protein	**4.33**
GBAA0169	rna polymerase sigma-70 factor, ecf subfamily	**5.68**
GBAA0179	hypothetical protein	**2.19**
GBAA0250	*acpS*; holo-(acyl-carrier-protein) synthase	**2.43**
GBAA0251	lipoprotein, putative	**2.56**
GBAA0370	methyl-accepting chemotaxis protein	**3.87**
GBAA0396	*proS-1*; prolyl-tRNA synthetase	**2.40**
GBAA0532	abc transporter, atp-binding protein	**2.27**
GBAA0533	abc transporter, permease protein, putative	**2.20**
GBAA0534	abc transporter, permease protein, putative	**2.05**
GBAA0535	potassium channel protein, putative	**2.14**
GBAA0558	methyl-accepting chemotaxis protein	**2.44**
GBAA0573	hypothetical protein	**4.98**
GBAA0574	hypothetical protein	**2.26**
GBAA0587	acetyltransferase, gnat family	**8.85**
GBAA0613	*sspH*; acid-soluble spore protein H	**2.06**
GBAA0614	proton/peptide symporter family protein	**3.12**
GBAA0623	hypothetical protein	**2.26**
GBAA0631	*treB*; pts system, trehalose-specific iibc component	**20.61**
GBAA0632	*treC*; trehalose-6-phosphate hydrolase	**26.81**
GBAA0644	hypothetical protein	**2.19**
GBAA0646	RNA polymerase sigma-70 factor	**2.63**
GBAA0647	hypothetical protein	**2.65**
GBAA0661	*glpT*; glycerol-3-phosphate transporter	**16.60**
GBAA0662	transcriptional regulator, marr family	**4.02**
GBAA0664	*rbsR*; ribose operon repressor	**2.05**
GBAA0665	*rbsK*; ribokinase	**2.46**
GBAA0666	*rbsD*; ribose abc transporter protein	**2.42**
GBAA0667	*rbsA*; ribose abc transporter, atp-binding protein	**2.24**
GBAA0684	methyl-accepting chemotaxis protein	**2.44**
GBAA0745	phospholipase, putative	**3.18**
GBAA0791	*celC-1*; pts system, cellobiose-specific iia component	**4.75**

**Table S8. Genes with increased expression in *B. anthracis* Sterne ($34F_2$) in MGM in O_2**		
***SEQUENCE ID**	**GENE INFO**	**Fold difference**
GBAA0792	*celA-1*; pts system, cellobiose-specific iib component	**5.44**
GBAA0793	*celB-1*; pts system, cellobiose-specific iic component	**4.20**
GBAA0794	hypothetical protein	**3.50**
GBAA0795	hypothetical protein	**3.00**
GBAA0866	*alsS*; alpha-acetolactate synthase	**2.54**
GBAA0981	s-layer protein, putative	**2.78**
GBAA1025	*glpF*; glycerol uptake facilitator protein	**26.23**
GBAA1026	*glpK*; glycerol kinase	**17.44**
GBAA1027	*glpD*; glycerol-3-phosphate dehydrogenase, aerobic	**64.69**
GBAA1125	methyl-accepting chemotaxis protein	**3.67**
GBAA1177	*clpB*; atp-dependent clp protease, atp-binding subunit clpb	**2.01**
GBAA1247	hypothetical protein	**2.61**
GBAA1248	*trpE*; anthranilate synthase component I	**6.34**
GBAA1249	*pabA-2*; anthranilate synthase component II	**4.71**
GBAA1250	anthranilate phosphoribosyltransferase	**3.47**
GBAA1251	indole-3-glycerol-phosphate synthase	**4.29**
GBAA1252	*trpF*; N-(5'-phosphoribosyl)anthranilate isomerase	**3.71**
GBAA1253	*trpB*; tryptophan synthase subunit beta	**3.04**
GBAA1254	*trpA*; tryptophan synthase subunit alpha	**2.77**
GBAA1314	transcriptional regulator, gntr family	**2.49**
GBAA1374	abc transporter, atp-binding protein	**2.02**
GBAA1375	abc transporter, permease protein, putative	**2.82**
GBAA1376	abc transporter, atp-binding protein	**2.31**
GBAA1629	*cspB-1*; cold shock protein cspb	**2.22**
GBAA1662	flagellar motor switch protein	**2.14**
GBAA1694	hypothetical protein	**2.22**
GBAA1701	hypothetical protein	**4.20**
GBAA1753	RNA polymerase sigma-70 factor	**2.60**
GBAA1754	hypothetical protein	**3.58**
GBAA1799	proton/sodium-glutamate symporter	**13.04**
GBAA1800	*aspA-2*; aspartate ammonia-lyase	**7.03**
GBAA1801	*ykwA*; malate oxidoreductase	**5.46**
GBAA1802	sensor histidine kinase	**4.17**
GBAA1803	response regulator	**3.91**
GBAA1862	acetyl-coa hydrolase/transferase family protein	**4.36**
GBAA1880	putative manganese transport protein MntH	**2.01**
GBAA1926	s-layer protein, putative	**5.04**
GBAA1958	*fabG*; 3-ketoacyl-(acyl-carrier-protein) reductase	**2.64**
GBAA1998	*nadE*; NAD(+) synthetase	**2.19**
GBAA2011	hypothetical protein	**2.55**
GBAA2020	oxidoreductase, aldo/keto reductase family	**3.43**
GBAA2032	hypothetical protein	**2.21**
GBAA2115	hypothetical protein	**2.65**
GBAA2118	hypothetical protein	**4.39**

Table S8. Genes with increased expression in *B. anthracis* Sterne ($34F_2$) in MGM in O_2		
***SEQUENCE ID**	**GENE INFO**	**Fold difference**
GBAA2186	*aspS-1*; asparaginyl-tRNA synthetase	**2.43**
GBAA2193	tpr domain protein	**2.04**
GBAA2197	hypothetical protein	**2.23**
GBAA2201	site-specific recombinase, resolvase family, putative	**2.28**
GBAA2202	transposase, is110 family, orfa	**2.07**
GBAA2216	sodium-dependent transporter, putative	**5.97**
GBAA2290	hypothetical protein	**4.30**
GBAA2315	s-layer protein, putative	**2.41**
GBAA2367	oxalate:formate antiporter, putative	**77.66**
GBAA2369	*dhbC*; isochorismate synthase	**2.16**
GBAA2370	*dhbE*; 2,3-dihydroxybenzoate-amp ligase	**2.00**
GBAA2388	*thrS-1*; threonyl-tRNA synthetase	**3.05**
GBAA2441	hypothetical protein	**3.18**
GBAA2442	hypothetical protein	**3.36**
GBAA2443	abc transporter, atp-binding/permease protein	**3.57**
GBAA2444	abc transporter, atp-binding/permease protein	**3.11**
GBAA2562	conserved hypothetical protein tigr00488	**2.15**
GBAA2563	hypothetical protein	**3.21**
GBAA2564	5'-methylthioadenosine/S-adenosylhomocysteine nucleosidase	**2.92**
GBAA2566	acetyltransferase, gnat family	**2.76**
GBAA2731	hypothetical protein	**2.20**
GBAA2732	RNA polymerase sigma-70 factor	**2.96**
GBAA2742	hypothetical protein	**3.08**
GBAA2743	hypothetical protein	**3.04**
GBAA2744	acetyltransferase, gnat family	**3.21**
GBAA2808	hypothetical protein	**2.04**
GBAA2809	hypothetical protein	**2.62**
GBAA2836	sensory box sigma-54 dependent dna-binding response regulator	**2.71**
GBAA2837	hypothetical protein	**2.96**
GBAA2842	deda family protein	**4.59**
GBAA2855	cytidine deaminase	**2.09**
GBAA2866	5'-methylthioadenosine/S-adenosylhomocysteine nucleosidase	**2.42**
GBAA2867	hypothetical protein	**2.44**
GBAA2868	glycosyl transferase, wecb/taga/cpsf family	**2.57**
GBAA2869	peptidase, putative	**2.47**
GBAA2886	hypothetical protein	**3.31**
GBAA2938	acetyltransferase, gnat family	**2.36**
GBAA2939	acetyltransferase, gnat family	**2.50**
GBAA2940	hypothetical protein	**2.58**
GBAA2941	hypothetical protein	**2.80**
GBAA2947	sulfatase	**3.25**
GBAA2955	*hisC-2*; putative aminotransferase	**2.71**
GBAA2956	*aroF-2*; chorismate synthase	**2.51**
GBAA2957	hypothetical protein	**3.24**

Table S8. Genes with increased expression in *B. anthracis* Sterne ($34F_2$) in MGM in O_2		
***SEQUENCE ID**	**GENE INFO**	**Fold difference**
GBAA2958	3-deoxy-7-phosphoheptulonate synthase	**2.16**
GBAA2988	permease, putative	**4.39**
GBAA2989	transcriptional regulator, arsr family	**4.15**
GBAA2992	*proA*; gamma-glutamyl phosphate reductase	**2.05**
GBAA2993	*proB*; gamma-glutamyl kinase	**3.29**
GBAA3020	major facilitator family transporter	**6.81**
GBAA3032	adenine deaminase, putative	**2.72**
GBAA3051	hypothetical protein	**3.04**
GBAA3077	hypothetical protein	**2.07**
GBAA3089	NAD-dependent deacetylase	**2.67**
GBAA3090	*pcP*; pyrrolidone-carboxylate peptidase	**2.61**
GBAA3093	hypothetical protein	**3.00**
GBAA3094	hypothetical protein	**3.26**
GBAA3095	hypothetical protein	**4.52**
GBAA3096	urea amidolyase-related protein	**5.40**
GBAA3097	conserved hypothetical protein tigr00370	**4.60**
GBAA3098	transcriptional regulator, iclr family	**4.30**
GBAA3136	*aspA-3*; aspartate ammonia-lyase	**6.85**
GBAA3137	*ansA-2*; l-asparaginase	**3.04**
GBAA3138	*ansR*; transcriptional regulator ansr	**4.37**
GBAA3140	hypothetical protein	**2.41**
GBAA3141	amino acid permease family protein	**5.95**
GBAA3142	*brnQ-5*; branched-chain amino acid transport system ii carrier protein	**9.35**
GBAA3143	*proC*; pyrroline-5-carboxylate reductase	**14.23**
GBAA3144	hypothetical protein	**18.75**
GBAA3145	malate dehydrogenase, putative	**18.64**
GBAA3146	hypothetical protein	**6.87**
GBAA3147	hypothetical protein	**17.49**
GBAA3153	response regulator	**2.21**
GBAA3155	*glsA-2*; glutaminase	**16.03**
GBAA3162	5'-nucleotidase, putative	**3.56**
GBAA3187	hydrolase, alpha/beta fold family	**2.72**
GBAA3246	hypothetical protein	**2.14**
GBAA3247	hypothetical protein	**2.54**
GBAA3248	3-beta hydroxysteroid dehydrogenase/isomerase family protein	**2.78**
GBAA3250	hypothetical protein	**3.16**
GBAA3251	3-oxoacyl-(acyl carrier protein) synthase	**3.31**
GBAA3254	lpxtg-motif cell wall anchor domain protein	**4.19**
GBAA3291	methyl-accepting chemotaxis protein	**4.58**
GBAA3323	hypothetical protein	**5.42**
GBAA3324	RNA polymerase sigma-70 factor	**4.41**
GBAA3335	hypothetical protein	**2.32**
GBAA3391	hypothetical protein	**2.05**
GBAA3396	hypothetical protein	**2.13**

Table S8. Genes with increased expression in *B. anthracis* Sterne ($34F_2$) in MGM in O_2		
***SEQUENCE ID**	**GENE INFO**	**Fold difference**
GBAA3406	abc transporter, atp-binding protein	**2.67**
GBAA3408	tryptophanyl-tRNA synthetase	**2.06**
GBAA3433	*zwf*; glucose-6-phosphate 1-dehydrogenase	**2.23**
GBAA3438	alcohol dehydrogenase, zinc-containing	**2.36**
GBAA3482	hypothetical protein	**2.57**
GBAA3538	hypothetical protein	**4.60**
GBAA3560	glycerophosphoryl diester phosphodiesterase, putative	**40.91**
GBAA3603	acetyltransferase, gnat family	**2.77**
GBAA3614	rard protein	**2.38**
GBAA3629	hypothetical protein	**2.03**
GBAA3647	transcription antiterminator, lytr family	**6.79**
GBAA3648	hypothetical protein	**8.66**
GBAA3649	rna polymerase sigma-70 factor, ecf subfamily	**9.15**
GBAA3655	oxidoreductase, gfo/idh/moca family	**2.34**
GBAA3729	als operon regulatory protein alsr, putative	**2.28**
GBAA3845	hypothetical protein	**10.12**
GBAA3907	hypothetical protein	**2.05**
GBAA4033	hypothetical protein	**3.22**
GBAA4271	hydrolase, haloacid dehalogenase-like family	**2.18**
GBAA4321	hypothetical protein	**2.01**
GBAA4346	*cpdB*; 2',3'-cyclic nucleotide 2'-phosphodiesterase/3'-nucleotidase bifunctional periplasmic precursor protein	**3.39**
GBAA4431	lipoate-protein ligase a, putative	**2.77**
GBAA4435	*sugE-1*; suge protein	**2.39**
GBAA4571	nitroreductase family protein	**2.82**
GBAA4746	acid phosphatase	**2.57**
GBAA4790	*brnQ-6*; branched-chain amino acid transport system ii carrier protein	**10.55**
GBAA4812	drug resistance transporter, emrb/qaca family	**2.86**
GBAA4813	hypothetical protein	**4.90**
GBAA4961	drug resistance transporter, emrb/qaca family	**3.99**
GBAA4988	hypothetical protein	**2.43**
GBAA5064	*feoB*; ferrous iron transport protein b	**2.04**
GBAA5217	abc transporter, atp-binding protein	**2.23**
GBAA5253	proline dehydrogenase family protein	**2.30**
GBAA5255	conserved hypothetical protein tigr00106	**2.32**
GBAA5256	methyl-accepting chemotaxis protein	**5.15**
GBAA5261	amino acid permease family protein	**5.25**
GBAA5262	hypothetical protein	**8.52**
GBAA5283	hypothetical protein	**2.03**
GBAA5284	transcriptional regulator, marr family	**2.14**
GBAA5301	sodium/alanine symporter family protein	**12.98**
GBAA5314	*tyrS-2*; tyrosyl-tRNA synthetase	**2.45**
GBAA5317	methyl-accepting chemotaxis protein	**2.85**
GBAA5318	endonuclease/exonuclease/phosphatase family	**3.46**

**Table S8. Genes with increased expression in *B. anthracis* Sterne ($34F_2$) in MGM in O_2**		
***SEQUENCE ID**	**GENE INFO**	**Fold difference**
GBAA5345	hypothetical protein	**2.71**
GBAA5346	hypothetical protein	**2.24**
GBAA5431	conserved hypothetical protein tigr00257	**2.06**
GBAA5432	transcription antiterminator, lytr family	**2.20**
GBAA5498	dna-binding protein	**2.85**
GBAA5528	*spoIID*; stage ii sporulation protein d	**3.13**
GBAA5559	conserved hypothetical protein tigr01440	**2.27**
GBAA5560	ribose-5-phosphate isomerase B	**2.07**
GBAA5563	pts system, glucose-specific iia component, putative	**2.51**
GBAA5582	hypothetical protein	**3.01**
GBAA5628	iron compound abc transporter, iron compound-binding protein	**3.88**
GBAA5636	*pta*; phosphate acetyltransferase	**3.74**
GBAA5649	abc transporter, permease protein	**2.25**
GBAA5650	abc transporter, atp-binding protein	**2.94**
GBAA5651	lipase/acylhydrolase, putative	**2.59**
GBAA5663	*thiD-2*; phosphomethylpyrimidine kinase	**2.01**
GBAA5667	response regulator, putative	**2.50**
GBAA5703	atp-dependent rna helicase, dead/deah box family	**2.33**
GBAApXO1_0129	utp-glucose-1-phosphate uridylyltransferase, (pxo1-94)	**2.88**

*Sequence ID numbers are GBAA locus tags from the *Bacillus anthracis* Ames Ancestor genome.
