# Supplementary material for: Comparative Transcriptional Profiling of Bacillus cereus Sensu Lato Strains during Growth in CO2-Bicarbonate and Aerobic Atmospheres
Source: PLoS One. 2009 Mar 19;4(3):e4904. doi: 10.1371/journal.pone.0004904 (PMC2654142; doi:10.1371/journal.pone.0004904)
Supplement: Table S9 — Genes with increased expression in B. cereus 10987 in CO2 (MGM+0.8% bicarbonate) (0.12 MB PDF) [file pone.0004904.s009.pdf]

Table S9. Genes with increased expression in *B. cereus* 10987 in CO_2 (MGM + 0.8% bicarbonate)		
**SEQUENCE ID**	**GENE INFO**	**Fold difference**
BCE_A0001	Tn554-related, transposase B	**2.90**
BCE_A0002	Tn554-related, transposase C	**2.07**
BCE_A0242	Tn554-related, transposase A	**2.20**
BCE0148	spore germination protein GerD	**2.08**
BCE0149	kinb signaling pathway activation protein	**2.24**
BCE0260	4-hydroxyphenylpyruvate dioxygenase	**3.20**
BCE0261	fumarylacetoacetate hydrolase family protein	**3.58**
BCE0262	homogentisate 1,2-dioxygenase, putative	**2.99**
BCE0433	mandelate racemase/muconate lactonizing enzyme family protein	**2.08**
BCE0434	transcriptional regulator domain protein	**2.59**
BCE0435	Na+/H+ antiporter NhaC	**2.61**
BCE0469	amino acid ABC transporter, ATP-binding protein	**3.38**
BCE0478	hypothetical protein	**2.66**
BCE0519	cation-transporting ATPase, E1-E2 family	**8.46**
BCE0554	PTS system, N-acetylglucosamine-specific IIBC component, putative	**2.74**
BCE0585	glutamate synthase, large subunit, putative	**4.89**
BCE0617	hypothetical protein	**2.68**
BCE0618	SPFH domain/band 7 family protein	**2.88**
BCE0641	citrate cation symporter family	**8.28**
BCE0642	malate dehydrogenase, putative	**5.99**
BCE0687	hypothetical protein	**2.07**
BCE0724	oligopeptide ABC transporter, oligopeptide-binding protein	**4.57**
BCE0725	oligopeptide ABC transporter, permease protein	**4.61**
BCE0726	oligopeptide ABC transporter, permease protein	**4.95**
BCE0727	oligopeptide ABC transporter, ATP-binding protein	**3.93**
BCE0880	hypothetical protein	**4.68**
BCE0883	PTS system, cellobiose-specific IIB component	**2.57**
BCE0884	PTS system, cellobiose-specific IIC component	**2.88**
BCE0885	hypothetical protein	**3.57**
BCE0886	hypothetical protein	**2.59**
BCE0913	hypothetical protein	**2.10**
BCE0915	hypothetical protein	**2.00**
BCE0925	sugE protein, putative	**3.34**
BCE0926	sugE protein, putative	**2.50**
BCE0962	hypothetical protein	**2.10**
BCE0979	enoyl-CoA hydratase	**3.44**
BCE1106	hypothetical protein	**5.47**
BCE1107	hypothetical protein	**3.13**
BCE1115	glyoxalase family protein	**3.68**
BCE1116	glyoxylase family protein	**3.95**
BCE1193	acyl-CoA synthase	**5.42**
BCE1425	maoC family protein	**2.16**
BCE1427	transcriptional regulator, PadR family	**2.41**
BCE1428	phaR protein	**3.13**

Table S9. Genes with increased expression in *B. cereus* 10987 in CO_2 (MGM + 0.8% bicarbonate)		
**SEQUENCE ID**	**GENE INFO**	**Fold difference**
BCE1429	3-ketoacyl-(acyl-carrier-protein) reductase	**3.14**
BCE1430	poly(R)-hydroxyalkanoic acid synthase, class III, PhaC subunit	**3.57**
BCE1453	hypothetical protein	**4.27**
BCE1564	hypothetical protein	**2.28**
BCE1565	branched-chain amino acid transport system II carrier protein	**2.16**
BCE1571	flavohemoprotein	**2.20**
BCE1624	hypothetical protein	**2.23**
BCE1651	menaquinol-cytochrome c reductase, cytochrome b/c subunit	**2.40**
BCE1729	germination protein gerN	**14.02**
BCE1798	transcriptional regulator, TetR family	**2.48**
BCE1817	short chain dehydrogenase	**5.58**
BCE1899	extracellular solute-binding protein, putative	**2.07**
BCE2035	hypothetical protein	**2.31**
BCE2230	hypothetical protein	**2.09**
BCE2251	hypothetical protein	**2.21**
BCE2264	hypothetical protein	**4.57**
BCE2269	acyl carrier protein phosphodiesterase	**3.11**
BCE2328	hypothetical protein	**2.11**
BCE2330	CoA-transferase, beta subunit	**2.45**
BCE2377	2-methylcitrate dehydratase	**2.44**
BCE2378	carboxyvinyl-carboxyphosphonatephosphorylmutase	**3.16**
BCE2379	hypothetical protein	**4.79**
BCE2380	acyl-CoA dehydrogenase	**3.60**
BCE2381	2-hydroxy-3-oxopropionate reductase	**3.19**
BCE2382	methylmalonic acid semialdehyde dehydrogenase	**2.50**
BCE2384	enoyl-CoA hydratase	**2.51**
BCE2490	O-methyltransferase family protein	**2.66**
BCE2553	carboxyl transferase domain protein	**3.22**
BCE2554	acetoacetyl-CoA synthase, putative	**2.49**
BCE2752	spore germination protein XC	**3.12**
BCE2753	spore germination protein XA	**2.90**
BCE2754	spore germination protein XB	**2.11**
BCE2794	acetoin operon transcriptional activator, putative	**3.76**
BCE2869	hypothetical protein	**7.76**
BCE2870	hypothetical protein	**6.83**
BCE2871	hypothetical protein	**3.01**
BCE2990	membrane protein, MmpL family, putative	**2.26**
BCE3034	amino acid permease family protein	**2.12**
BCE3147	Tn554-related, transposase A	**2.11**
BCE3148	Tn554-related, transposase B	**2.83**
BCE3149	Tn554-related, transposase C	**2.11**
BCE3401	Integral membrane protein domain protein	**2.11**
BCE3609	ABC transporter, permease protein	**2.09**
BCE3610	ABC transporter, ATP-binding protein	**2.93**

Table S9. Genes with increased expression in *B. cereus* 10987 in CO_2 (MGM + 0.8% bicarbonate)		
**SEQUENCE ID**	**GENE INFO**	**Fold difference**
BCE3621	anaerobic ribonucleoside-triphosphate reductase activating protein	**5.10**
BCE3622	anaerobic ribonucleoside triphosphate reductase	**8.40**
BCE3653	hypothetical protein	**2.34**
BCE3671	phospholipase/carboxylesterase family protein	**3.14**
BCE3672	glyoxylase family protein	**3.27**
BCE3677	formimidoylglutamase	**6.40**
BCE3678	imidazolonepropionase	**4.93**
BCE3679	urocanate hydratase	**4.18**
BCE3680	histidine ammonia-lyase	**5.07**
BCE3695	cytochrome c-type biogenesis protein CcdA	**2.23**
BCE3738	site-specific recombinase, phage integrase family	**2.32**
BCE3797	sulfatase	**4.01**
BCE3857	reverse transcriptase/endonuclease protein	**2.39**
BCE3927	orotate phosphoribosyltransferase	**5.11**
BCE3928	orotidine 5'-phosphate decarboxylase	**5.24**
BCE3929	dihydroorotate dehydrogenase	**4.73**
BCE3930	dihydroorotate dehydrogenase electron transfer subunit	**5.26**
BCE3931	carbamoyl-phosphate synthase large subunit	**6.47**
BCE3932	carbamoyl-phosphate synthase small subunit	**6.13**
BCE3933	dihydroorotase	**6.38**
BCE3934	aspartate carbamoyltransferase catalytic subunit	**5.28**
BCE3987	hypothetical protein	**2.98**
BCE3988	cytochrome c oxidase, subunit IVB	**4.02**
BCE3989	cytochrome c oxidase, subunit III	**4.07**
BCE3990	cytochrome c oxidase, subunit I	**3.34**
BCE3991	cytochrome c oxidase, subunit II	**2.86**
BCE3998	PhoH family protein	**2.06**
BCE4063	maltosaccharide ABC transporter, permease protein	**2.43**
BCE4064	maltosaccharide ABC transporter, maltosaccharide-binding protein, putative	**2.25**
BCE4078	hypothetical protein	**2.50**
BCE4172	hypothetical protein	**2.33**
BCE4173	sugar-binding transcriptional regulator, LacI family, putative	**2.25**
BCE4178	hypothetical protein	**3.11**
BCE4180	riboflavin synthase subunit alpha	**2.77**
BCE4181	bifunctional 3,4-dihydroxy-2-butanone 4-phosphate synthase/GTP cyclohydrolase II protein	**4.06**
BCE4182	riboflavin synthase subunit beta	**3.06**
BCE4310	hypothetical protein	**3.02**
BCE4332	5-methyltetrahydrofolate--homocysteine methyltransferase	**2.13**
BCE4649	electron transfer flavoprotein, alpha subunit	**2.25**
BCE4653	acyl-CoA synthase	**2.80**
BCE4778	inorganic polyphosphate/ATP-NAD kinase	**3.15**
BCE4781	acetyl-CoA synthetase, putative	**3.41**
BCE4801	acetyl-coenzyme A synthetase	**2.12**

**Table S9. Genes with increased expression in *B. cereus* 10987 in CO_2 (MGM + 0.8% bicarbonate)**		
**SEQUENCE ID**	**GENE INFO**	**Fold difference**
BCE4802	acetoin utilization protein AcuA	**2.96**
BCE4803	acetoin utilization protein AcuB	**3.34**
BCE4922	hypothetical protein	**2.49**
BCE4950	cytochrome d ubiquinol oxidase, subunit II	**7.47**
BCE4973	hypothetical protein	**2.32**
BCE5000	carbohydrate kinase, PfkB family	**2.07**
BCE5002	pyridoxal phosphate-dependent enzyme, putative	**2.13**
BCE5003	dihydroorotase	**2.46**
BCE5004	hypothetical protein	**2.42**
BCE5005	hypothetical protein	**2.99**
BCE5006	hypothetical protein	**3.48**
BCE5007	hypothetical protein	**4.46**
BCE5024	glycogen phosphorylase	**3.60**
BCE5025	glycogen synthase	**4.41**
BCE5026	glycogen biosynthesis protein GlgD	**4.70**
BCE5027	glucose-1-phosphate adenylyltransferase	**3.26**
BCE5028	glycogen branching enzyme	**3.52**
BCE5083	CAAX amino terminal protease family protein	**3.09**
BCE5091	hypothetical protein	**2.44**
BCE5092	hypothetical protein	**2.31**
BCE5093	hypothetical protein	**2.29**
BCE5107	phosphoglycerate mutase family protein, putative	**2.30**
BCE5108	hypothetical protein	**3.04**
BCE5132	lipoprotein, putative	**3.00**
BCE5133	PAP2 family protein	**6.25**
BCE5142	acyl-CoA dehydrogenase	**12.47**
BCE5143	acetyl-CoA acetyltransferase	**10.16**
BCE5144	3-hydroxyacyl-CoA dehydrogenase/enoyl-CoA hydratase/isomerase family protein	**11.77**
BCE5195	hypothetical protein	**2.21**
BCE5229	hypothetical protein	**4.01**
BCE5328	hypothetical protein	**3.26**
BCE5351	D-amino acid aminotransferase	**2.22**
BCE5352	hypothetical protein	**2.46**
BCE5416	NADH dehydrogenase subunit M	**2.23**
BCE5417	NADH dehydrogenase subunit L	**2.94**
BCE5418	NADH dehydrogenase kappa subunit	**2.16**
BCE5419	NADH dehydrogenase subunit J	**3.69**
BCE5420	NADH dehydrogenase subunit I	**3.25**
BCE5422	NADH dehydrogenase delta subunit	**2.95**
BCE5423	NADH dehydrogenase subunit C	**2.81**
BCE5424	NADH dehydrogenase beta subunit	**2.86**
BCE5471	transcriptional regulator, TetR family	**4.06**
BCE5472	acyl-CoA dehydrogenase	**4.68**

**Table S9. Genes with increased expression in *B. cereus* 10987 in CO_2 (MGM + 0.8% bicarbonate)**		
**SEQUENCE ID**	**GENE INFO**	**Fold difference**
BCE5473	acyl-CoA dehydrogenase	**4.66**
BCE5474	3-hydroxybutyryl-CoA dehydrogenase	**4.20**
BCE5475	acetyl-CoA acetyltransferase	**4.79**
BCE5476	ferredoxin, 4Fe-4S	**4.38**
BCE5488	aminopeptidase, putative	**2.10**
BCE5489	hypothetical protein	**2.12**
BCE5534	homoserine O-succinyltransferase	**2.08**
BCE5596	oligoendopeptidase F, putative	**2.12**
