# Supplementary material for: Comparative Transcriptional Profiling of Bacillus cereus Sensu Lato Strains during Growth in CO2-Bicarbonate and Aerobic Atmospheres
Source: PLoS One. 2009 Mar 19;4(3):e4904. doi: 10.1371/journal.pone.0004904 (PMC2654142; doi:10.1371/journal.pone.0004904)
Supplement: Table S10 — Genes with increased expression in B. cereus 10987 in MGM in O2 (0.10 MB PDF) [file pone.0004904.s010.pdf]

Table S10. Genes with increased expression in *B. cereus* 10987 in MGM in O_2		
**SEQUENCE ID**	**GENE INFO**	**Fold difference**
BCE0158	D-fructose-6-phosphate amidotransferase	**2.25**
BCE0363	RNA methyltransferase, TrmA family	**2.01**
BCE0407	hypothetical protein	**2.03**
BCE0683	iron compound ABC transporter, iron compound-binding protein	**2.29**
BCE0730	transcriptional regulator, MarR family	**3.23**
BCE0731	drug resistance transporter, EmrB/QacA family	**2.72**
BCE0808	hypothetical protein	**4.36**
BCE0809	potassium-transporting ATPase subunit A	**4.37**
BCE0810	potassium-transporting ATPase subunit B	**2.99**
BCE0811	potassium-transporting ATPase, C subunit	**2.65**
BCE0887	hypothetical protein	**4.25**
BCE1137	hypothetical protein	**2.22**
BCE1140	hypothetical protein	**2.10**
BCE1224	transporter, EamA family	**2.77**
BCE1409	glycolate oxidase, iron-sulfur subunit, putative	**15.08**
BCE1410	glycolate oxidase, subunit GlcD	**21.41**
BCE1415	transcriptional regulator, GntR family	**2.09**
BCE1461	hypothetical protein	**3.29**
BCE1493	hypothetical protein	**2.62**
BCE1840	Na+/H+ antiporter NhaC	**4.34**
BCE1857	lipoprotein, putative	**2.47**
BCE1859	hypothetical protein	**2.88**
BCE1860	hypothetical protein	**2.74**
BCE1861	RNA polymerase sigma-70 factor	**2.08**
BCE1880	asparagine synthetase AsnA	**4.98**
BCE1923	sodium-dependent transporter	**3.07**
BCE1951	chlorohydrolase	**2.02**
BCE1966	hypothetical protein	**2.54**
BCE2044	3-ketoacyl-(acyl-carrier-protein) reductase	**2.22**
BCE2072	hypothetical protein	**2.34**
BCE2121	hypothetical protein	**2.90**
BCE2201	hypothetical protein	**3.09**
BCE2215	hypothetical protein	**2.60**
BCE2396	oxalate:formate antiporter, putative	**78.18**
BCE2419	threonyl-tRNA synthetase	**2.18**
BCE2455	CAAX amino terminal protease family protein	**2.26**
BCE2472	hypothetical protein	**2.71**
BCE2473	hypothetical protein	**2.80**
BCE2515	hypothetical protein	**2.22**
BCE2536	penicillin-binding protein	**2.00**
BCE2743	hypothetical protein	**2.32**
BCE2799	lipoprotein, putative	**2.55**
BCE2830	methylase	**2.07**

Table S10. Genes with increased expression in *B. cereus* 10987 in MGM in O_2		
**SEQUENCE ID**	**GENE INFO**	**Fold difference**
BCE2836	hypothetical protein	**2.19**
BCE2906	degV family protein	**2.97**
BCE2979	EF0108	**2.68**
BCE2987	sulfatase	**2.45**
BCE3028	gamma-glutamyl phosphate reductase	**2.29**
BCE3029	gamma-glutamyl kinase	**3.16**
BCE3030	hypothetical protein	**4.24**
BCE3100	hypothetical protein	**2.05**
BCE3101	hypothetical protein	**2.35**
BCE3104	lysine-specific permease	**4.41**
BCE3157	5'-nucleotidase, putative	**2.37**
BCE3170	hypothetical protein	**2.63**
BCE3190	hypothetical protein	**2.90**
BCE3191	NADH dehydrogenase subunit L	**3.88**
BCE3244	hypothetical protein	**2.13**
BCE3460	phosphoesterase, putative subfamily	**2.80**
BCE3485	iron compound ABC transporter, iron compound-binding protein, putative	**4.64**
BCE3486	iron compound ABC transporter, permease protein	**2.44**
BCE3487	iron compound ABC transporter, permease protein	**2.60**
BCE3554	BNR repeat domain protein	**2.48**
BCE3555	flavodoxin	**2.57**
BCE3556	hypothetical protein	**2.07**
BCE3605	transcription antiterminator, LytR family	**2.03**
BCE3606	hypothetical protein	**3.22**
BCE3607	RNA polymerase sigma-70 factor, ECF subfamily	**2.83**
BCE3697	hypothetical protein	**3.01**
BCE3743	hypothetical protein	**2.86**
BCE3767	hypothetical protein	**2.45**
BCE3771	iron compound ABC transporter, iron compound-binding protein	**2.03**
BCE3970	hypothetical protein	**2.23**
BCE4036	hypothetical protein	**2.11**
BCE4070	hypothetical protein	**2.53**
BCE4152	xanthine/uracil permease family protein	**5.23**
BCE4208	L-serine dehydratase, iron-sulfur-dependent, alpha subunit	**2.15**
BCE4209	L-serine dehydratase, iron-sulfur-dependent, beta subunit	**2.38**
BCE4314	prolyl 4-hydroxylase, alpha subunit domain protein	**2.40**
BCE4392	conserved hypothetical protein TIGR00046	**2.09**
BCE4450	iron compound ABC transporter, iron compound-binding protein	**2.74**
BCE4484	hesA/moeB/thiF family protein	**2.61**
BCE4488	hypothetical protein	**2.08**
BCE4490	D-tyrosyl-tRNA deacylase	**2.14**
BCE4636	ABC transporter, ATP-binding protein	**2.06**
BCE4638	acid phosphatase	**2.02**
BCE4665	hypothetical protein	**2.24**

Table S10. Genes with increased expression in *B. cereus* 10987 in MGM in O_2		
**SEQUENCE ID**	**GENE INFO**	**Fold difference**
BCE4700	hypothetical protein	**2.47**
BCE4712	S-adenosylmethionine decarboxylase proenzyme	**2.07**
BCE4883	permease, putative	**2.42**
BCE4932	hypothetical protein	**2.02**
BCE4952	S-layer protein, putative	**2.07**
BCE5017	1,4-dihydroxy-2-naphthoate octaprenyltransferase,	**2.25**
BCE5064	pyridine nucleotide-disulphide oxidoreductase	**3.08**
BCE5160	hypothetical protein	**4.98**
BCE5181	methyl-accepting chemotaxis protein	**3.06**
BCE5197	hypothetical protein	**2.86**
BCE5198	nucleoside transporter, NupC family	**2.62**
BCE5201	sodium/alanine symporter family protein	**2.40**
BCE5206	oxidoreductase, aldo/keto reductase family	**2.03**
BCE5207	major facilitator family transporter	**2.33**
BCE5215	endonuclease/exonuclease/phosphatase family	**2.70**
BCE5235	hypothetical protein	**5.87**
BCE5354	nucleoside transporter, NupC family	**14.05**
BCE5404	mbl protein	**2.31**
BCE5455	modification methylase, HemK family	**2.15**
BCE5516	phosphate acetyltransferase	**2.17**
BCE5571	antiholin-like protein LrgB	**44.70**
BCE5572	murein hydrolase regulator LrgA	**13.05**
