# Supplementary material for: Comparative Transcriptional Profiling of Bacillus cereus Sensu Lato Strains during Growth in CO2-Bicarbonate and Aerobic Atmospheres
Source: PLoS One. 2009 Mar 19;4(3):e4904. doi: 10.1371/journal.pone.0004904 (PMC2654142; doi:10.1371/journal.pone.0004904)
Supplement: Table S11 — Primers for SYBR-Green qRT-PCR for B. anthracis Sterne 34F2 (0.08 MB PDF) [file pone.0004904.s011.pdf]

1Table S1. Primers for SYBR-Green qRT-PCR for *B. anthracis* Sterne 34F$_2$			
**sequence i.d.**	**common name**	**primer name**	***primer sequence**
GBAA1945	transport atp-binding protein *cydc*	1945 for	AGATGGACAACTTGCAATGGA
		1945 rev	TATAGCGCCATTTCCATTCCA
GBAA0887	s-layer protein ea1	0887 for	TGCAATTACAGGTAAGCCAGA
		0887 rev	ACCTTTAACAACGCCAGCTTT
GBAA2367	oxalate:formate antiporter, putative	2367 for	TCTCTGCATATGGTTCTGGTA
		2367 rev	TGTTTCCTGAACTGCACTTTG
GBAA3649	sigma 70 family rna polymerase, ecf family	3649 for	GAGGATGCGATACAAACAACA
		3649 rev	ATCGCACGCACTCATATTGTA
pXO1 0164	*pagA*	pXO1 0164 for	GAATTTTCAAGCACCCATGGT
		pXO1 0164 rev	TCAGCGGAAGTAGCAAATGTA
pXO1 0146	*atxA*	pXO1 0146 for	CAAGTAGGAGCTTTATACCCA
		pXO1 0146 rev	AGGGAAACGGCCAATAATCAT
pXO1 0137	hypothetical	pXO1 0137 for	AGCTGAGAGATATGAGCTCAA
		pXO1 0137 rev	AGATGTCCGTGTGTTAGATAA
GBAA0107	*fusA* (elongation factor EF-2)	0107 for	AGGTCACGTAGATTTCACAGT
		0107 rev	GAGTATAAGAAATCTGCACC

*Primer sequences based on *B. anthracis* Ames Ancestor Genome NC_007530
