# Supplementary material for: Comparative Transcriptional Profiling of Bacillus cereus Sensu Lato Strains during Growth in CO2-Bicarbonate and Aerobic Atmospheres
Source: PLoS One. 2009 Mar 19;4(3):e4904. doi: 10.1371/journal.pone.0004904 (PMC2654142; doi:10.1371/journal.pone.0004904)
Supplement: Table S12 — Primers for SYBR-Green qRT-PCR for B. cereus G9241 (0.08 MB PDF) [file pone.0004904.s012.pdf]

Table S12. Primers for SYBR-Green qRT-PCR for *B. cereus* G9241				
**sequence i.d.**	**common name**	**contig number**	**primer name**	**primer sequence**
BCE_G9241_1390	flavodoxin	AAEK01000009	G1390 for	ATGGTGACCTTCCTGATGATT
			G1390 rev	AGTCCTTCTAACACAACTGTG
BCE_G9241_2201	penicillin binding protein	AAEK01000073	G2201 for	ATGAGGCTCTTTGGTATAGCT
			G2201 rev	ATGTTCTGTAGCGTATTGCTC
BCE_G9241_0658	phospholipase c precursor	AAEK01000035	G0658 for	CACTTGTAAAGCAAGATCGAG
			G0658 rev	TAGCTCCAGTTTCTTTTGCCT
BCE_G9241_2676	bacillolysin	AAEK01000129	G2676 for	ATTAACGGGCACTGGAAAAGA
			G2676 rev	ACTAGTTGCTAATGTACCAGG
BCE_G9241 pBC218_0045	transcriptional regulator *arsR* family	AAEK01000004	G-218-0045 for	ATTAGAAGAGGATGCTGAGCT
			G-218-0045 rev	TCTAGTCCTTTTCGATTCCCT
BCE_G9241 pBC218_0026	protective antigen	AAEK01000004	G-218-0026 for	TCCAATCTGCAATTTGGACAG
			G-218-0026 rev	AGAAGGGGATTCTCGTTGATA
BCE_G9241 pBC218_0027	lethal factor precursor	AAEK01000004	G-218-0027 for	TGGTATACACGTAAGGCTGAA
			G-218-0027 rev	TCTTATCCAAATCGCCTCCT
BCE_G9241 pBC218_0064	UTP-glucose-1-phosphate uridylyltransferase - *galU*	AAEK01000004	G-218-0064 for	TCGTCGTTATCAAGTACGTCA
			G-218-0064 rev	AATCGTTGAATCGCATCCGTT
BCE_G9241 pBCXO1_0109	UTP-glucose-1-phosphate uridylyltransferase - *galU*	AAEK01000036	GX-0109 for	TACGTATCCATGTCTCAAGCA
			GX-0109 rev	TCCAGAGGTGGTTTTTCAACT
BCE_G9241 pBCXO1_0105	S-layer homology domain protein	AAEK01000036	GX-0105 for	TCTGGACGTTCCATCAAATCA
			GX-0105 rev	ATGGATTTTGAGCTTCTGCCT
BCE_G9241 pBCXO1_0106	PAP2 superfamily domain protein	AAEK01000036	GX-0106 for	AAAGTACTAGGTATCGCCACA
			GX-0106 rev	ATCCTGTCATTGCATAACGGA
BCE_G9241_0105	*fusA* homolog	AAEK01000143	G0105 for	TGTTAGACGCAGTTATCGACT
			G0105 rev	AGTACACACGGAAGAACGTTA
