# Supplementary material for: Comparative Transcriptional Profiling of Bacillus cereus Sensu Lato Strains during Growth in CO2-Bicarbonate and Aerobic Atmospheres
Source: PLoS One. 2009 Mar 19;4(3):e4904. doi: 10.1371/journal.pone.0004904 (PMC2654142; doi:10.1371/journal.pone.0004904)
Supplement: Table S13 — Putative motility genes more highly expressed in O2 for B. cereus G9241 and B. anthracis Sterne (34F2) (0.08 MB PDF) [file pone.0004904.s013.pdf]

**Table S13: Putative motility genes more highly expressed in O_2 for *B. cereus* G9241 and *B. anthracis* Sterne ($34F_2$)**		
**Locus Tag #**	**Gene Name**	**Fold Difference in O_2**
***B. cereus* G9241**		
BCE_G9241_0373	methyl-accepting_chemotaxis_protein_putative	**3.40**
BCE_G9241_0538	methyl-accepting_chemotaxis_protein	**2.53**
BCE_G9241_0550	methyl-accepting_chemotaxis_transducer_putative	**5.03**
BCE_G9241_0665	methyl-accepting_chemotaxis_protein_putative	**4.43**
BCE_G9241_1669	sodium-driven_polar_flagellar_protein_PomB_putative	**3.48**
BCE_G9241_1670	chemotaxis_response_regulator	**6.32**
BCE_G9241_1671	histidine_kinase_(cheA)	**6.42**
BCE_G9241_1672	chemotaxis_protein_cheC	**4.21**
BCE_G9241_1675	chemotaxis_protein_methyltransferase_putative	**2.43**
BCE_G9241_1679	flagellar_hook-associated_protein_1_putative	**5.64**
BCE_G9241_1680	flagellar_hook-associated_protein_flgL_putative	**5.56**
BCE_G9241_1681	flagellar_hook-associated_protein_2	**5.25**
BCE_G9241_1682	flagellar_protein_putative	**5.07**
BCE_G9241_1684	flagellar_basal-body_rod_protein_flgB	**12.05**
BCE_G9241_1685	flagellar_basal-body_rod_protein_flgC	**9.78**
BCE_G9241_1686	flagellar_hook-basal_body_complex_protein_fliE	**15.92**
BCE_G9241_1687	flagellar_M-ring_protein_putative	**12.91**
BCE_G9241_1688	flagellar_motor_switch_protein_fliG	**6.45**
BCE_G9241_1690	type_III_secretion_cytoplasmic_ATPase_SctN_TC0040	**4.64**
BCE_G9241_1694	flagellar_hook_protein_flgE_putative	**9.79**
BCE_G9241_1697	chemotaxis_protein_cheV_VC1602	**5.01**
BCE_G9241_1702	flagellin	**7.47**
BCE_G9241_1706	flagellar_motor_switch_protein_(fliM)_putative	**6.41**
BCE_G9241_1707	flagellar_motor_switch_protein_fliN_putative	**5.93**
BCE_G9241_1709	flagellar_biosynthetic_protein_fliP	**6.48**
BCE_G9241_1710	flagellar_biosynthesis_protein_fliQ	**3.97**
BCE_G9241_1711	flagellar_biosynthetic_protein_fliR_putative	**7.96**
BCE_G9241_1712	flagellar_biosynthesis_protein_flhB	**7.11**
BCE_G9241_1713	flagellar_biosynthesis_protein_flhA	**5.45**
BCE_G9241_1715	flagellar_basal-body_rod_protein_flgF_putative	**3.14**
BCE_G9241_5113	methyl-accepting_chemotaxis_transducer_putative	**5.64**
BCE_G9241_5185	methyl-accepting_chemotaxis_protein_putative	**3.43**
BCE_G9241_CNI_0304	flagellar_biosynthetic_protein_fliR_putative	**8.58**
***B. anthracis* Sterne ($34F_2$)**		
GBAA0370	methyl-accepting chemotaxis protein	**3.87**

**Table S13: Putative motility genes more highly expressed in O_2 for *B. cereus* G9241 and *B. anthracis* Sterne ($34F_2$)**		
**Locus Tag #**	**Gene Name**	**Fold Difference in O_2**
GBAA0558	methyl-accepting chemotaxis protein	**2.44**
GBAA0684	methyl-accepting chemotaxis protein	**2.44**
GBAA1125	methyl-accepting chemotaxis protein	**3.67**
GBAA1662	flagellar motor switch protein	**2.14**
GBAA3291	methyl-accepting chemotaxis protein	**4.58**
GBAA5256	methyl-accepting chemotaxis protein	**5.15**
GBAA5317	methyl-accepting chemotaxis protein	**2.85**
