# Supplementary material for: Comparative Transcriptional Profiling of Bacillus cereus Sensu Lato Strains during Growth in CO2-Bicarbonate and Aerobic Atmospheres
Source: PLoS One. 2009 Mar 19;4(3):e4904. doi: 10.1371/journal.pone.0004904 (PMC2654142; doi:10.1371/journal.pone.0004904)
Supplement: Table S14 — Putative S-Layer genes differentially expressed in CO2 and O2 in three Bacilli. (0.08 MB PDF) [file pone.0004904.s014.pdf]

Table S14. Putative S-Layer genes differentially expressed in CO_2 and O_2 in three *Bacilli*.

*Locus Tag #	Gene name	Fold Difference in CO_2	Fold Difference in O_2
B. cereus **G9241**			
0890	S-layer protein precursor	x	3.18
0996	S-layer homology domain	x	28.32
1115	S-layer homology domain	2.52	x
4903	S-layer homology domain	x	9.51
pBC218_0012	S-layer protein / peptidoglycan endo-beta-N-acetylglucosaminidase	28.56	x
pBC218_0043	S-layer homology domain protein	41.00	x
pBCXO1_0105	S-layer homology domain protein	12.59	x
B. anthracis **Sterne ($34F_2$)**			
0887	S-layer protein ea1	21.25	x
0981	S-layer protein, putative	x	2.78
1130	S-layer protein, putative	6.54	x
1926	S-layer protein, putative	x	5.04
2315	S-layer protein, putative	x	2.41
pXO1_0124	S-layer protein, (pxo1-90)	35.13	x
B. cereus **10987**			
4952	S-layer protein, putative	x	2.07

*Locus tag numbers are from the *B. cereus* G9241 (BCE_G9241_XXXX), *B. anthracis* Ames Ancestor (GBAAXXXX) and the *B. cereus* 10987 (BCE_XXXX) genomes.
